# A guide to barley mutants

**DOI:** 10.1186/s41065-023-00304-w

**Published:** 2024-03-08

**Authors:** Mats Hansson, Helmy M. Youssef, Shakhira Zakhrabekova, David Stuart, Jan T. Svensson, Christoph Dockter, Nils Stein, Robbie Waugh, Udda Lundqvist, Jerome Franckowiak

**Affiliations:** 1 Department of Biology, Lund University, Sölvegatan 35, 22362 Lund, Sweden; 2 Faculty of Agriculture, Cairo University, Giza, 12613 Egypt; 3 Institute of Agricultural and Nutritional Sciences, Faculty of Natural Sciences III, Martin Luther University Halle-Wittenberg, Halle, 06120 Germany; 4 Nordic Genetic Resource Center (NordGen), Växthusvägen 12, 23456 Alnarp, Sweden; 5 Carlsberg Research Laboratory, J. C. Jacobsens Gade 4, 1799 Copenhagen V, Denmark; 6 Leibniz Institute of Plant Genetics and Crop Plant Research (IPK), OT Gatersleben, Stadt Seeland, E06466 Germany; 7 Center for Integrated Breeding Research (CiBreed), Georg-August-University, Göttingen, Germany; 8 Cell and Molecular Sciences, The James Hutton Institute, Invergowrie, Dundee, DD2 5DA UK; 9 Division of Plant Sciences, University of Dundee, The James Hutton Institute, Invergowrie, Dundee, DD2 5DA UK; 10 School of Agriculture Food and Wine, Waite Campus, The University of Adelaide, Urrbrae, 5064 Australia; 11 Department of Agronomy and Plant Genetics, University of Minnesota Twin Cities, 411 Borlaug Hall, 1991 Upper Buford Circle, St Paul, MN 55108 USA

**Keywords:** Barley, Biodiversity, Cereal, Genebank, *Hordeum vulgare*, Induced mutants, Mutagenesis, Mutation, Triticeae

## Abstract

**Background:**

Mutants have had a fundamental impact upon scientific and applied genetics. They have paved the way for the molecular and genomic era, and most of today’s crop plants are derived from breeding programs involving mutagenic treatments.

**Results:**

Barley (*Hordeum vulgare* L.) is one of the most widely grown cereals in the world and has a long history as a crop plant. Barley breeding started more than 100 years ago and large breeding programs have collected and generated a wide range of natural and induced mutants, which often were deposited in genebanks around the world. In recent years, an increased interest in genetic diversity has brought many historic mutants into focus because the collections are regarded as valuable resources for understanding the genetic control of barley biology and barley breeding. The increased interest has been fueled also by recent advances in genomic research, which provided new tools and possibilities to analyze and reveal the genetic diversity of mutant collections.

**Conclusion:**

Since detailed knowledge about phenotypic characters of the mutants is the key to success of genetic and genomic studies, we here provide a comprehensive description of mostly morphological barley mutants. The review is closely linked to the International Database for Barley Genes and Barley Genetic Stocks (bgs.nordgen.org) where further details and additional images of each mutant described in this review can be found.

## Background

The molecular era started with mutants. In their more drastic forms, mutants display a clear observable character, i.e. they show a phenotype, distinct from the so-called wild type. At the same time, they carry genetic material that contains a modification in the DNA sequence that causes the mutant phenotype. By studying the mutant, it is possible to find the causal mutation underlying the observable character, thus revealing the original function of the gene. Following this approach, major biochemical pathways were revealed from studies of bacterial mutants. More recently, mutants in more complex organisms such as plants have been used to address questions related to diverse aspects of plant biology including differentiation, development and the interaction with the environment. This has been fueled by tremendous progress in genome sequencing of organisms with large genomes. In barley (*Hordeum vulgare* L.), the availability of a reference genome sequence [1] and thousands of mutants is a good match with high potential. The access to a reference genome facilitates all aspects of gene identification in mutants from comparative genomic approaches to map-based cloning and direct genome sequencing [2].

In barley, mutants have been known for over 100 years. The extant chlorophyll mutants *xan-m.3* (from the Xantha mutant group) and *alb-c.7* (Albina mutant group) were used by early geneticists to investigate the basic concepts of Mendelian inheritance [3–7]. The yellow Xantha and white Albina mutants were used because their clear and obvious phenotype could be scored already at the seedling stage. While the chlorophyll mutants were of theoretical rather than applied use, the short-culm mutant *uzu1.a* is an example of a mutant that was identified very early but in contrast to the chlorophyll mutations became widespread in cultivars [8]. In the 1930s, 70% of barley grown in Japan was of the *uzu*-type and seventy years later, the *uzu1.a* mutant allele was found in most Japanese hull-less barley cultivars [9].

Shortly after the discovery that ionizing irradiation could increase mutation frequency in fruit fly [10], barley researchers applied this technique [4, 11, 12]. Soon after, mutations were also induced by chemicals [13, 14]. The efforts resulted in large numbers of mutations of which most had unfavorable effects from a practical point of view. Initially, it was not understood that original mutants should be regarded as raw breeding material, which had to be refined by backcrosses into non-mutated plant material. In the early days, this sometimes resulted in a pessimistic view regarding the usefulness of induced mutagenesis for breeding [15]. However, soon mutants were obtained that could be used in breeding programs. These mutants had changed properties in quantitative traits related to, for example, straw-length, straw-stiffness, seed size, number of tillers and early maturity. One of the most common groups were the Erectoides mutants, characterized by an erect spike, which is compact or dense, as well as a culm that is often short and stiff. The Erectoides mutant *ert-k.32* was induced by X-rays in the cultivar Bonus in 1947 and released as a new cultivar named Pallas in 1958 [16]. Pallas was the first barley cultivar released on the market that originated from an induced mutant. Two years later, Pallas was followed by cultivar Mari, which was developed from the early maturity mutant *mat-a.8* obtained after X-ray treatment of Bonus in 1951 [17].

Many of the thousands of mutants that have been isolated by barley researchers and breeders now resides at various genebanks and seed stores around the world such as the Nordic Genetic Resource Center (Sweden), the Institute of Plant Science and Resources at Okayama University (Japan), the USDA National Small Grains Collection (USA), and the IPK Federal ex situ Gene Bank at the Leibniz Institute of Plant Genetics and Crop Plant Research (IPK) at Gatersleben (Germany). A more comprehensive list of germplasm collections can be found in van Hintum and Menting [18]. However, it is also important to note that large numbers of new mutants are still induced every year by barley researchers and breeders [19, 20]. Mutations in the first generation are usually heterozygous and an additional round of self-pollination is required to reveal the phenotype of recessive mutations, which are more frequent than dominant mutations. Mutagenesis of microspores is an attractive alternative since the method produces double-haploid plants that are fixed homozygotes [21]. In addition to radiation and chemical mutagenesis, New Genomic Techniques (NGTs), including CRISPR technology, are emerging for targeted mutagenesis [22]. CRISPR technology is currently semi-specific in that a mutation can be induced at an approximate position determined by a guide-RNA. Currently, it is difficult to predict the exact location and exact type of mutation, but it is likely that the site-specific gene editing approaches mediated by RNA-guided Cas9 endonucleases will soon overcome these hurdles allowing plant biologists to change any specific codon in a gene and introduce foreign genes into plants. We therefore see an increasing interest for mutants in the barley and cereal research community. We have written this review with the aim of describing the current classification and phenotypes of existing barley mutants, which will remain a valuable reference material for future studies. The review is tightly linked to the International Database for Barley Genes and Barley Genetic Stocks (bgs.nordgen.org) where further details and additional images of each mutant described in this review can be found. Many of the images in the database and this publication show comparisons between cultivar Bowman and near-isogenic lines of the mutants, which were back crossed to Bowman [23]. The database does not include descriptions of root mutants [24]. Therefore, such mutants are not covered in the present work.

### Standard nomenclature of genes, mutations and mutants

The nomenclature of genes, mutations, and mutants tends to be very complex and has evolved differently within different scientific communities. Historic and local traditions increase the complexity. Recommendations for the naming of barley genes, mutations, and mutants have been a recurrent topic at the International Barley Genetics Symposium, which is held on average every fourth year since 1963. At this symposium, an International Committee for Nomenclature and Symbolization of Barley Genes was appointed. Their recommendations have been published in several issues of the Barley Genetics Newsletter; the latest in volume 49 [25].

In barley nomenclature, every mutation / mutant / locus is associated with a name and a symbol. The name should be as descriptive as possible of the phenotype. The name is written in non-italic font and initiated with a capital letter. The symbol should consist of three letters and be written in italic. The symbol can be used to describe the locus, the gene, the mutant, and a particular mutation or allele. The symbol is written with lower case letters if the mutation is recessive. If the mutation is dominant, the first letter is capitalized. A typical example of a symbol of a recessive mutation is *uzu1.a*, which can then be used to also describe the mutant, the locus and the specific allele. This mutation causes a deficiency in the kinase domain of the brassinosteroid receptor [26, 27]. The *uzu1.a* allele has a long history in short-culm cultivars in Japan, the Korean peninsula and China [9, 28]. The word “uzu” describes the character of the mutant and is an abbreviation of the Japanese word “uzutakai”. The term refers to a state in which many things are piled up or stacked, but the stack obtains a rounded, and not sharp and pointed, appearance (Takao Komatsuda, personal communication). The description fits recessive *uzu1* mutants carrying mutations in the *uzu1* locus on chromosome 3H, which are short with almost a cute appearance. A different locus where mutations would lead to an *uzu1*-like phenotype could then be given the symbol *uzu2*. The “*a*” in *uzu1.a* refers to a particular allele at the *uzu1* locus. Another mutant with a short culm phenotype is *ari-256*, isolated by Scandinavian researchers [29]. The symbol *ari* is for the Breviaristatum phenotype, which refers to the short awns of this mutant. It was later found that *ari-256* was also deficient in the brassinosteroid receptor and allelic to the *uzu1.a* mutant. Since the locus of *ari-256* was previously not determined, its name was changed to *uzu1.256* [27]. It should be noted that also the wild type can be described by an allele symbol. For example, the wild-type allele of the locus with the name Six-rowed spike 1 (symbol *vrs1*) is *Vrs1.b*, which is dominant and causes a two-rowed phenotype. One of the recessive alleles causing a six-rowed phenotype is *vrs1.a* [30]. *Vrs1.b*/*vrs1.a* can be used to describe a heterozygous mutant carrying the wild-type allele on one chromosome and the mutant allele on the other.

### Introduction to barley morphology

**Fig. 1:**
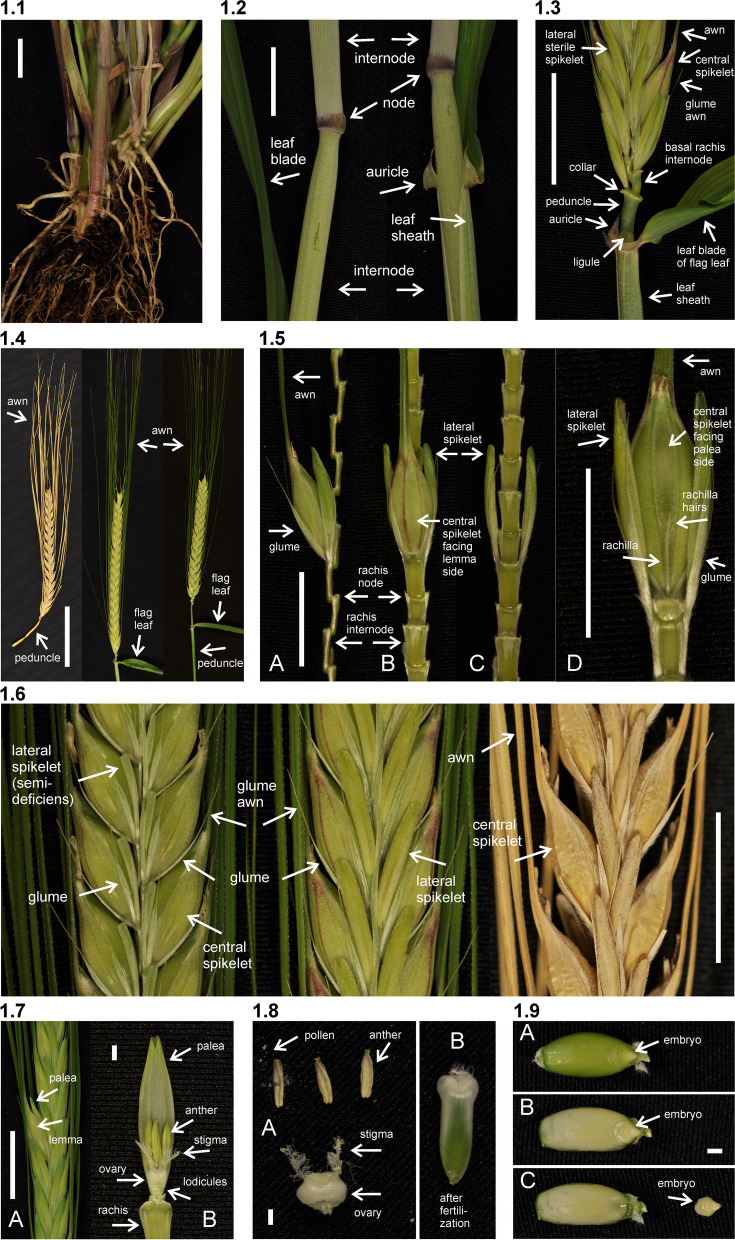
** 1.1** The root system of an adult barley plant is mainly formed by postembryonic nodal roots. The white bar corresponds to 1 cm. **1.2** Two culms showing the node flanked by two internodes. The culm is enclosed by the leaf sheath that is the lower part of the leaf. The auricle is at the junction between the leaf sheath and the leaf blade. The white bar corresponds to 1 cm. **1.3** The top leaf of each tiller is called the flag leaf. Like other leaves, the flag leaf consists of a sheath and a blade as well as a ligule and an auricle at the junction between them. The junction between a barley spike and culm is called collar. The peduncle is the first (top) internode of the culm. The white bar corresponds to 1 cm. **1.4** The spike is the reproductive part of barley. A spike is composed of approximately 20 to 30 successive triplets of spikelets, which consist of one central and two lateral spikelets. In the two-rowed spikes shown in the figure, only the central spikelet has a fertile floret. The awns extend from the floret of the central spikelets and are usually as long as the spike. The top leaf is called flag leaf. The peduncle is the top (first) internode of the culm. The white bar corresponds to 5 cm. **1.5** The stem of the spike is called rachis, which consists of rachis nodes and rachis internodes. The spikelets are attached at the rachis nodes. The figure shows two-rowed barley where the lateral spikelets are sterile. **A and B.** All spikelets except the one triplet of spikelets attached at one rachis node have been removed. The flower is surrounded by the lemma, the palea and two glumes. The awn is an extension of the lemma. The palea is inside the lemma and closest to the rachis. **C.** The central spikelet has been removed. **D.** The rachis has been removed to show the palea side of the central spikelet with the rachilla attached slightly below the palea. The rachilla is a rudimentary branch of the rachis. The white bars correspond to 1 cm. **1.6** Close-up of three barley spikes, which are of the two-rowed type with sterile lateral spikelets. In the left spike, the lateral spikelets are very small. The central spikelet is fertile and has resulted in a mature seed in the right spike. The white bar corresponds to 1 cm. **1.7 A.** The floret is composed of two leaf-like structures, the lemma and the palea. In the shown spike, all fertile flowers, except one, have been pollinated and therefore remain closed. In the open floret, the lemma and palea are separated exposing the cavity where the flower organs are kept. The bar corresponds to 1 cm. **B.** In the dissected floret, the upper rachis and the lemma have been removed to show the ovary with the featherlike stigmata and the three anthers (still not mature in the photo). The lodicules, situated between the palea and the lemma, can swell and thereby push away the lemma to facilitate exposure of the anthers and stigmata. In barley, usually this happens exclusively if self-pollination fails and capture of pollen from other florets is required (like shown in **A**). The bar corresponds to 1 mm. **1.8 A.** Dissected anthers and ovary of a barley floret at the stage of pollination. Pollen can be seen dehiscing out from one anther. The stigma is dusty because it has received pollen. **B.** After a week the fertilized ovary has expanded longitudinally. The bar corresponds to 1 mm. **1.9** A barley grain one month after fertilization. **A.** The naked grain after removal of the lemma and palea. The embryo is facing the lemma side. **B.** The grain after removal of the seed-coat layer displaying the starch-containing endosperm. **C.** The embryo has been detached from the grain. The bar corresponds to 1 mm

Barley is an annual grass belonging to the *Poaceae* plant family. The fibrous root system consists of embryonic (seminal) and postembryonic roots. The seminal roots are formed during embryogenesis in contrast to the postembryonic roots, which are formed after germination [31]. After subcrown internode elongation, the postembryonic roots can emerge from basal parts of the shoot (nodal roots) as seminal roots (lateral roots) [32]. The nodal roots dominate the root system of adult cereal plants [33] (Fig. 1 (1.1)). The culm is cylindrical and hollow except at the nodes, to which the leaves are attached. The sections of the culm between the nodes are called internodes (Fig. 1 (1.2)). The culm typically consists of 5–6 visible internodes above the base of the plant and are numbered from the top to the bottom. The first internode is called the peduncle. The leaf consists of two parts; the basal sheath surrounding the culm and the distal blade [34]. A membrane-like structure named the ligule and two tabs named the auricle are found at the junction between the sheath and the blade (Fig. 1 (1.2 and 1.3)). The top leaf, surrounding the peduncle (culm internode 1), is called the flag leaf (Fig. 1 (1.3)). The inflorescence (the reproductive part) of the barley plant is in the form of a spike where the flowers are arranged in spikelets (Fig. 1 (1.3 and 1.4)). The structures found at the junction between the spike and the culm are the basal rachis internode, the collar and the peduncle (Fig. 1 (1.3)). The stem of the spike is called the rachis and has nodes and internodes. The spikelet is attached to the rachis node (Fig. 1 (1.5)). Each spikelet in barley has one floret (Fig. 1 (1.6 and 1.7)). The central spikelet is always fertile while the flanking lateral spikelets can be infertile (two-rowed barley) or fertile (six-rowed barley). The floret is surrounded by two bracts; the lowermost external lemma and the uppermost internal palea (Fig. 1 (1.5 and 1.7)). Glumes are additional sterile bracts, which are also parts of the spikelet. The awn is a characteristic feature of barley that protrudes from the spike from each floret as an extension of the lemma. Barley is hermaphroditic and the floret contains both three male stamens and a single female pistil. The anther is the part of the stamen that contains the pollen. The barley pistil consists of the ovary and two feather-like stigmata (Fig. 1 (1.8)). After pollination and fertilization, the emerging grain enlarges in size. The grain is composed of the seed coat (tissue of parental origin), the aleurone layer, the starchy endosperm and the embryo (all three filiate tissue), which is sitting as a small disc-like structure at the bottom of the grain (Fig. 1 (1.9)).

## Description of barley mutants

### Row type

**Table:** 

Keywords to find descriptions of mutants in the International Database for Barley Genes and Barley Genetic Stocks (bgs.nordgen.org):
6-rowed/2-rowed: distichon, hex, hexastichon, mul, multiflorus, six-rowed spike, vrs
Small lateral spikelets: deficiens, int, intermedium, intermedium spike, labile, large lateral spikelet, semideficiens, sls, small lateral spikelets, vrs

The barley spike is composed of spikelets in groups of three, with a single floret subtended by two glumes in each spikelet. Spikelet groups are arranged alternately at 20–30 rachis nodes. Each triplet consists of one central and two lateral spikelets. In two-rowed barley, the lateral spikelets are sterile (anthers can occur) and reduced in size, whereas they are fertile in six-rowed barley. Wild barley (*Hordeum vulgare* ssp. *spontaneum*), the progenitor of cultivated barley (*H. vulgare* ssp. *vulgare*), has a two-rowed spike and the triplet of spikelets forms an arrow-like structure that drives the kernel of the central spikelet into the soil after shattering of the spike at the rachis nodes. Six-rowed barley appeared during the process of barley domestication when Neolithic farmers deliberately selected for improved yield and seed recovery [30].

At first glance, the classification of row type into two- and six-rowed barley might seem straightforward. However, there is a complex system of incomplete dominance resulting in several intermediate forms with variation in the fertility, size, and shape of the lateral spikelets [35, 36]. The system for classification of row type follows to a large extent that of Harlan [37], Mansfeld [38, 39] and Hoffmann [40], which focuses on the number of rows with kernels and the fertility of lateral spikelets:

- Hexastichon. Six-rowed barley with all rows similar in fertility and the development of awns or hoods.
- Intermedium including Labile and Irregulare. Partial fertility of lateral spikelets is accompanied by irregular awn formation. Due to considerable variation within the spike further subdivision of this group was done [41], although not frequently used, into Divisa, being many-rowed only in the upper part of the spike; Incomposita, irregular many-rowed; Sola, with occasional fertile lateral spikelets; and Partita, only the upper lateral spikelets fertile, the basal ones sterile.
- Distichon. Two-rowed barley with sterile lateral spikelets.
- Deficiens. Two-rowed barley with rudimentary sterile lateral spikelets. Semi-deficiens with larger but still rudimentary sterile lateral spikelets.

#### Six-rowed and two-rowed barley

**Fig. 2:**
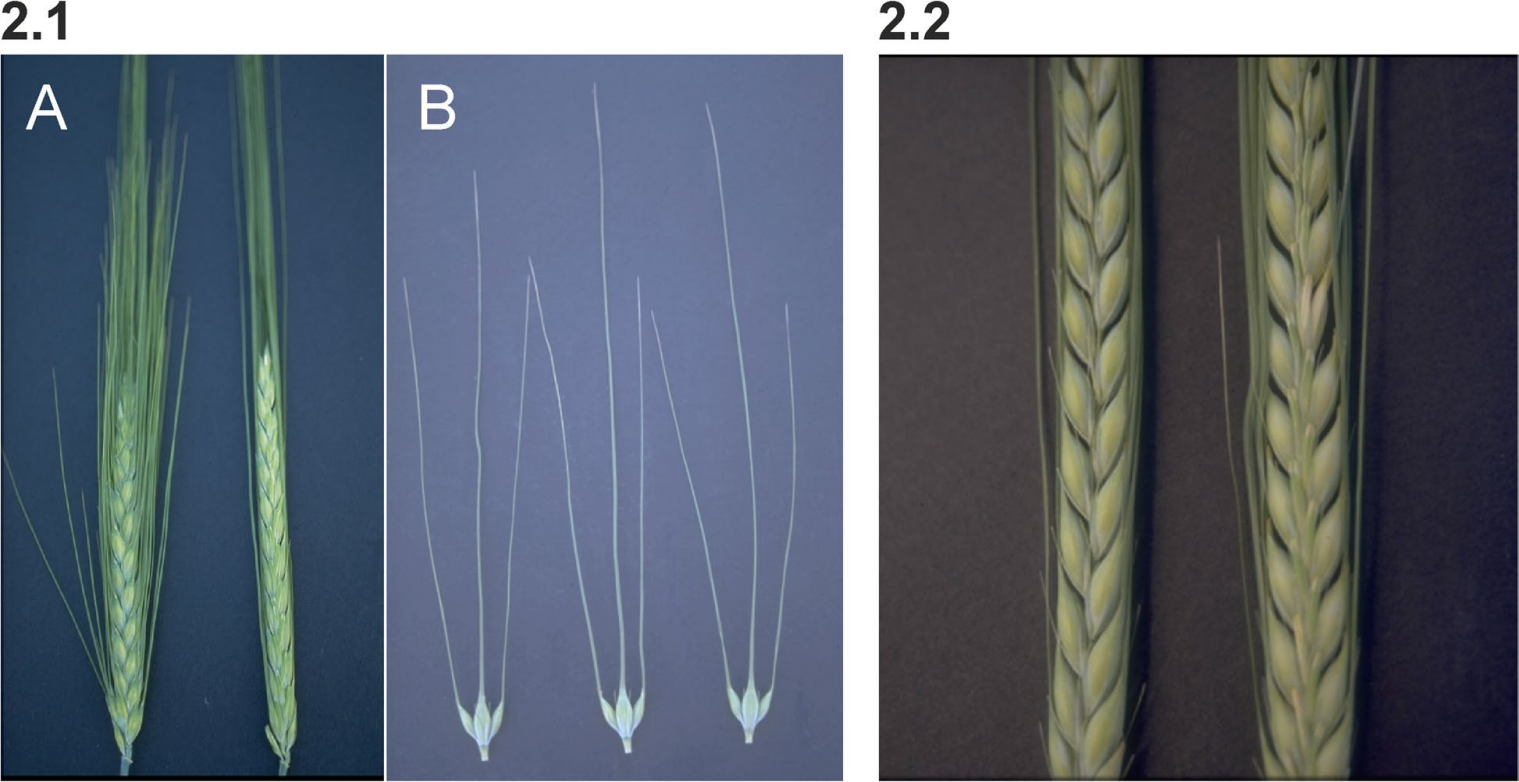
** 2.1 A.** Phenotype of a six-rowed Hexastichon barley *vrs1.a1* mutant (left) compared to cultivar Bowman with a two-rowed Distichion *Vrs1.b* spike (right). **B.** Spikelets of a *vrs1.a1* plant. Each central spikelet is flanked by two lateral spikelets. All spikelets are fertile in six-rowed barley. The awns of the lateral spikelets are shorter than the awn of the central spikelet. **2.2** A Deficiens (*Vrs1.t*) spike to the left compared to Bowman with a spike of Distichion (*Vrs1.b*) to the right. Deficiens barley has rudimentary lateral spikelets

The locus symbol used today for the major six-rowed gene is *vrs1* [42]. The gene encodes a transcription factor comprising a homeodomain and a closely linked leucine zipper motif [30]. Alleles at this complex locus modify the development of lateral spikelets and their associated lemma awn. The *vrs1.a* allele is present in most six-rowed cultivars and produces well-developed and fertile lateral spikelets throughout the spike [43]. The lemma awn of lateral spikelets can vary from 3/4 to nearly as long as that of the central spikelet, depending upon alleles present at other loci (Fig. 2 (2.1)). The *vrs1.c* allele produces six-rowed spikes with long awns on central spikelets and awn-like appendages on the completely fertile lateral spikelets [35]. The *Vrs1.b* allele is responsible for the Distichon phenotype and is present in most two-rowed cultivars. The sterile lateral spikelets show poorly developed lemma and palea with a rounded tip or apex subtended by two glumes. The *Vrs1.b* allele is also the wild-type allele in *Hordeum vulgare* ssp. *spontaneum* [30]. Interestingly, the *Vrs1.t* allele [44] causes the Deficiens phenotype with an extreme reduction in the size of sterile lateral spikelets (Fig. 2 (2.2)). More than 50 induced mutations in the *vrs1* locus have been isolated [42]. In addition, there is an occurrence of several alleles selected from spontaneous mutations in cultivated six-rowed barley. Based on phylogenetic analysis, it was suggested that six-rowed alleles originated independently several times in cultivated barley [30].

#### Small lateral spikelets—intermedium mutants

Two-rowed barley can produce mutants with spike development patterns intermediate between the two- and six-rowed states. Compared to the lateral spikelets of two-rowed barley, these mutants have enlarged lateral spikelets, which vary in fertility, kernel development, and awn length. Some of them can appear Hexastichon-like. Still, the Intermedium mutants form rather natural morphological groups with similar, however, particular traits. Some of these traits are shared. A total of 126 such Intermedium spike mutants were isolated by Scandinavian mutant researchers [45]. Of these mutants, 103 have been located at 11 different *int* loci by means of diallelic crosses. Most mutants are associated with the *int-a*, *-c*, *-d* and *-e* loci (Table 1).

**Table 1:** Number of allelic mutants at eleven different *int* loci

Locus *int*	*-a*	*-b*	*-c*	*-d*	*-e*	*-f*	*-h*	*-i*	*-k*	*-l*	*-m*
Frequency	23	2	18	13	7	1	3	1	1	1	1

Tests for inheritance of the *int* mutations demonstrated, in backcrosses to the mother cultivars, that mutants at eight of the above mentioned *int* loci are recessive, and only monogenic inheritance patterns were observed. The mutants belonging to the locus *int-d* showed different degrees of dominance to the two-rowed phenotype. One of the alleles of this locus seems to be completely dominant. F1 progenies from crosses of the other *int-d* mutants to mother cultivars, showed heterozygous plants that had lateral spikelets with lemmas having a pointed tip [46, 47]. Thus, these alleles are semidominant. It is now known that *int-d* mutants, as well as *hex-v* mutants, are alleles at the *vrs1* locus [30].

**Fig. 3:**
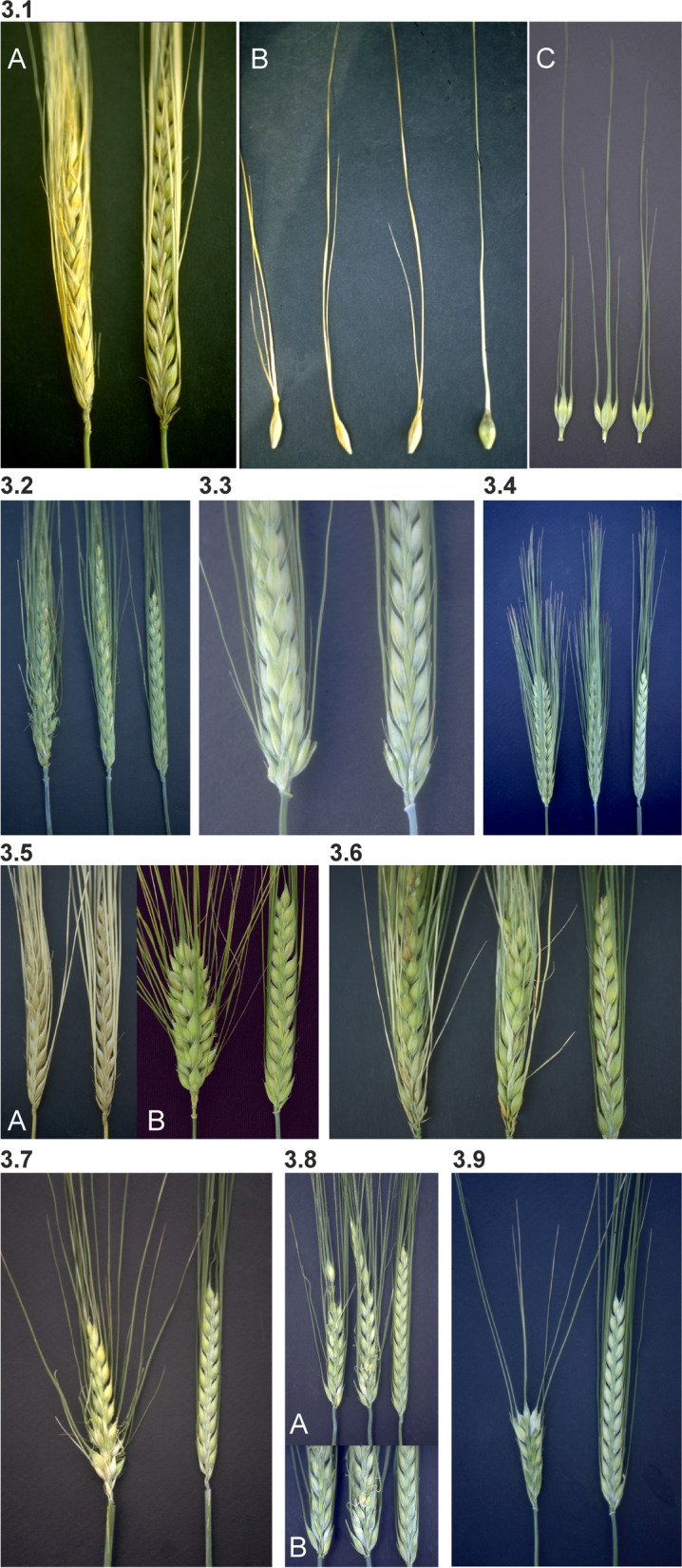
**3.1** Mutant Intermedium spike-a (*int-a.1*). **A.** Mutant to the left, cultivar Bowman to the right. The upper two-thirds of the spike has fertile lateral spikelets. **B.** Three mutant seeds with different double awn phenotypes to the left compared with normal Bowman. **C.** Three triplets of spikelets. The two lateral spikelets are smaller than the central spikelet, but still fertile. **3.2** Mutant Intermedium spike-b (*int-b.3*). The spike appears similar to the six-rowed spike, but developmental irregularities occur commonly in the lower half of the spike. All lateral spikelets are reduced in size, and their lemma awns are short or reduced to a pointed tip. Commonly, only lateral spikelets in the middle of the spike set seed. Cultivar Bowman is to the right. **3.3** Mutant Intermedium spike-c (*int-c.5*) to the left compared to Bowman. The lateral spikelets are fairly large and broad, the lemma is often rounded or weakly pointed, awnless or short-awned at the apex. Lower lateral spikelets may develop poorly in some *int-c* mutants, while seed development may occur in all lateral spikelets of others. Variability in lateral floret development exists among the *int-c* mutants and environmental conditions can alter expressivity. **3.4** *Int-d.12* (middle) compared to *hex-v.3* (left) and Bowman (right). Mutations in *int-d* are semidominant. The awns of lateral spikelets of *int-d* mutants will vary in length from ¾ to nearly as long as those of the central spikelets. Mutants in *int-d* are allelic to *vrs1* and *hex-v* [1]. **3.5** Mutants of the Six-rowed spike 4 (*vrs4*) locus. **A.** *int-e.58*. **B.** *mul1.a*. Mutants to the left compared to Bowman to the right. **3.6** Two spikes of mutant *int-f.19* compared to Bowman to the right. This locus is only represented by the *int-f.19* allele. The spike appears six-rowed, but the lateral spikelets are much smaller (less than half the size of the central spikelets). Lateral spikelets are pointed and often have short awns. Seed set occurs in the lateral spikelets in the upper third of the spike. The base of the spike has shortened rachis internodes and appears Erectoides-like [47]. **3.7** Mutant *int-h.42* to the left compared to Bowman. Lateral spikelets are enlarged and have an inconspicuously pointed apex, but they do not set seed. Induced mutants show early heading and have an elongated basal rachis internode. The spike appears *lax* but with shortened rachis internodes at the base [46]. A Bowman backcross-derived line is slightly shorter (5/6 normal) and produces extra spikelets (up to five fertile ones) at several rachis nodes in the lower half of the spike [50]. **3.8** Mutant Lower number of tillers 1 (*lnt1.a*) compared to Bowman to the right. This mutant is allelic to *int-l* [51]. Various spike malformations occur in most environments. The spike may have irregular rachis internode lengths. The lower portion of the spike appears denser. Lateral spikelets in two-rowed cultivars are enlarged and have a pointed apex. **B** is a close-up of the spikes shown in **A**. **3.9** Mutant *int-m.85* to the left compared to Bowman. The spike of *int-m* mutants is very short due to few rachis internodes and has irregular rachis internode lengths. Lateral spikelets are enlarged and pointed, but they do not set seed. The density of spikelets at the base of the spike is increased. Rachis internodes at the tip of the spike are very short, and the spike appears to have two or three fused terminal spikelets
- *int*-*b*: The spikes have a rather irregular shape; the lateral spikelets are conspicuously enlarged with partial seed set. The plant is tall and tillers poorly (Fig. 3 (3.2)). Mutants in *int-b* are allelic to *vrs2.e* [52].
- *int*-*c*: The lateral spikelets are relatively large and broad. The lemma is often rounded or weakly pointed at the apex. Lateral seed development is variable among mutants, among parts of the spike, and among different years (Fig. 3 (3.3)). Mutants in *int-c* are allelic to *vrs5.n* [53].
- *int*-*d*: This locus is marked by fairly large and distinctly pointed lateral spikelets, with short or long awns of variable length, but rarely reaching the lengths of the central spikelet awns (Fig. 3 (3.4)). The seed set of the lateral spikelets is variable, and in some mutants the laterals are completely sterile, while in other mutants they are partly or completely filled with seeds, although they are never as large as those of six-rowed mutants or cultivars. There is considerable variation in expression over years. The *int-d* mutants are allelic to *vrs1* and *hex-v* [30, 54].
- *int*-*e*: The lateral spikelets are enlarged and may set seeds in the upper two-third of the spike. The lateral spikelets have pointed tips. In the lower part of the spike, the lemma of the lateral spikelets is somewhat rounded at apex (Fig. 3 (3.5)). Mutants in *int-e* were induced in two-rowed cultivars and are allelic to *mul1* and *vrs4* [42]. The *mul1.a* and *vrs4.k* alleles were isolated in six-rowed cultivars and may produce two extra lateral spikelets at the base of each lateral spikelet [51].
- *int*-*f*: This locus has only one single mutant, with a typical dense Erectoides-like base. All the lateral spikelets are pointed, sometimes with short awns. The lateral spikelets of the upper part of the spike have a partial seed set (Fig. 3 (3.6)).
- *int*-*h*: The lateral spikelets are strongly enlarged, inconspicuously pointed at apex, mostly sterile with occasional awns. The spike has a Laxatum phenotype, and all three alleles are associated with early heading (Fig. 3 (3.7)).
- *int*-*i*: This locus is represented by a single mutant. The lateral spikelets are enlarged and partially pointed at the apex. The tip of the spike has shortened rachis internodes. Due to this character, the spike tip is of a very dense Erectoides type.
- *int*-*k*: The lateral spikelets are enlarged, pointed and completely sterile. Plants of the original stock have a dense coating of surface wax.
- *int*-*l*: Lateral spikelets in two-rowed cultivars are enlarged and have a pointed apex. Spike malformations occur in most environments. Spikes have irregular rachis internode lengths and are relatively short. The lower portion of the spike appears dense (Fig. 3 (3.8)). This mutant is allelic to Low number of tillers 1 (*lnt1*) [55]. The tiller number is reduced to 2 to 4 per plant. These tillers are formed soon after seedling emergence. That is, no late-emerging tillers are observed. Culms are thick and stiff, and leaves are dark green.
- *int*-*m*: The spike is very short and has irregular rachis internode lengths. Lateral spikelets are enlarged and pointed, but they do not set seed. Spikelet density at the base of the spike is increased. Rachis internodes at the tip of the spike are very short, and the spike appears to have two or three fused or fasciated terminal spikelets (Fig. 3 (3.9)). Tillering of *int-m* plants is increased and heading is slightly earlier [47].

The *int* mutants can be shortly described as follows:

- *int*-*a*: The lateral spikelets are characteristically enlarged with seed set in the upper two-thirds of the spike. The central spikelets often have double awns (Fig. 3 (3.1)). Mutants in *int-a* are allelic to the *vrs3* mutants [48, 49].

Changes in the size of sterile lateral spikelets have been noted and one variant is identified as a recessive allele at the Small lateral spikelet 1 (*sls1*) locus. It was isolated in two-rowed progeny from crosses between two- and six-rowed barley. The size of sterile lateral spikelets near the tip of the spike is reduced, but in some environments all lateral spikelets are less than half normal size. The phenotype associated with *sls1* is not expressed in six-rowed barley [56]. A modified six-rowed phenotype in which a portion of the spikelets are missing was identified as *Hordeum irregulare* [57]. Expression of the Irregulare or Labile phenotype is controlled by alleles at the *lab1* locus [58].

#### Double and triple mutant combinations of *int* genes

**Fig. 4:**
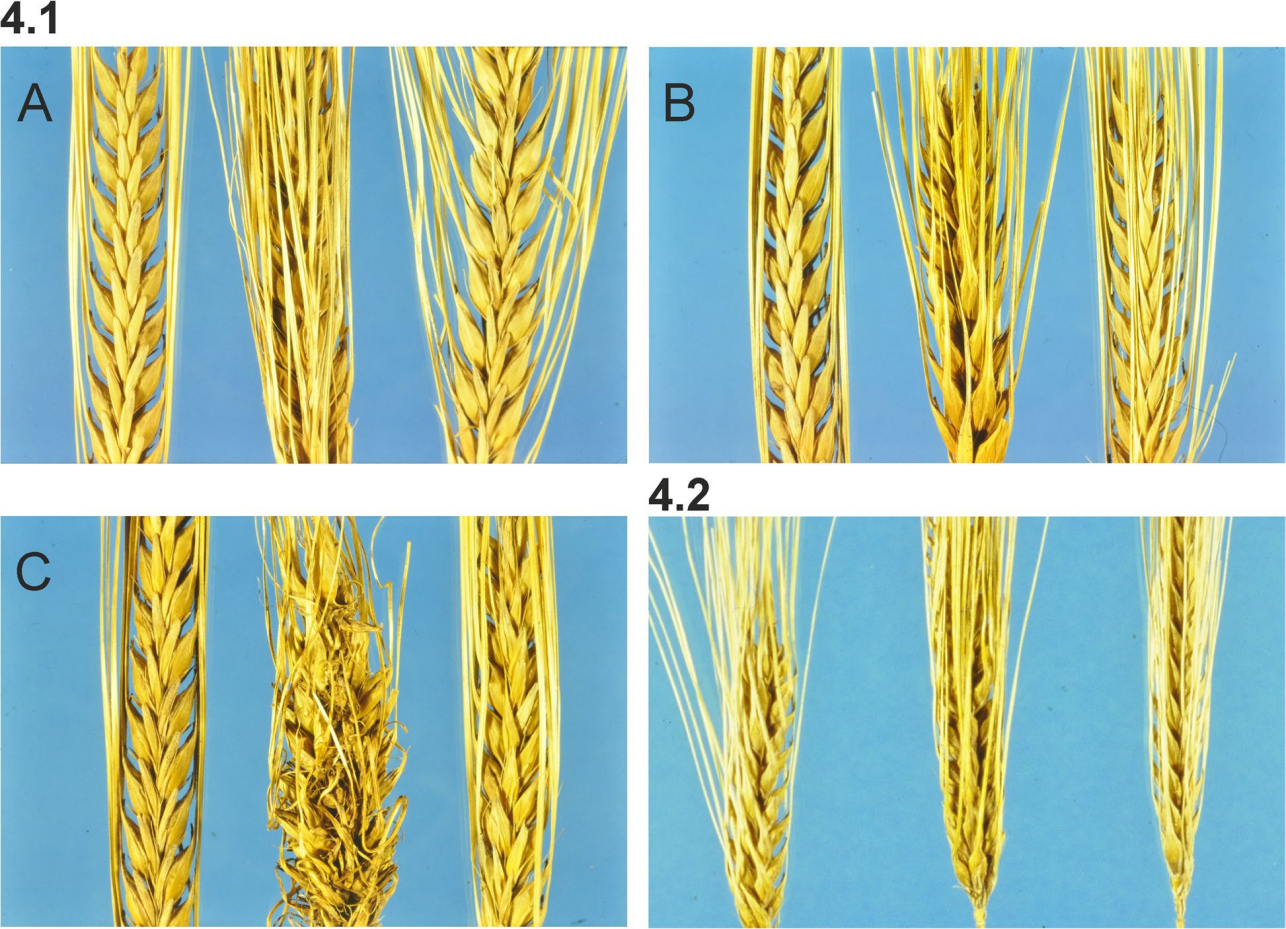
**4.1** Double mutant F_2_-segregants from crosses (middle) flanked by their *int* mutant parents. Most combinations of *int* mutants in the crosses result in double mutants with a typical six-rowed spike. **A.** From left to right: *int-c.15*, double mutant, *int-a.32*. **B.** From left to right: *int-c.16*, double mutant, *Int-d.28*. **C.** Deformed double mutant F_2_-segregant from poor combining partners. From left to right: *int-e.20*, double mutant, *int-a.46*. **4.2** Spikes of triple mutant combination from the six-rowed double mutant *int-c.5* *int-a.34* combined with the six-rowed mutant *hex-v.3*. From the left to right: *int-c.5* *int-a.34* double mutant, “King-size” spike of triple mutant *int-c.5* *int-a.34* *hex-v.3*, six-rowed mutant *hex-v.3*

Interaction between the *int* loci resulting in a further enhanced development of the lateral spikelets was observed at an early stage through crosses between various mutants [46]. Double mutants were identified in the F_2_ generations and frequently resulted in typical six-rowed spikes, whereas other double mutant combinations gave rise to irregular or deformed and even highly deformed spikes. The competence of *int* genes to interact efficiently, and its dependence on the interaction of particular loci and alleles, were investigated on a large set of material consisting of 1384 out of 1879 possible double mutant combinations [54]. There are apparent differences among *int* loci in their ability to co-operate in the formation of six-rowed spikes in double mutants. The most efficient combiners are *int*-*d* and *int*-*c*. It is striking that the two loci, *int*-*a* and *int*-*e* both interact successfully with *int*-*c* and *int*-*d*, and at the same time are quite inefficient partners to one another (Fig. 4 (4.1)).

Triple mutants were also investigated and often found to result in “King-size” spikes – beautiful six-row types with conspicuous large spikes and thick culms [59] (Fig. 4 (4.2)). At that time, the triple mutants could not be verified genetically but were obtained in crosses between *int*/*int* double mutants and *hex-v*. Certain combinations of *int* loci were more competent than others to produce King-size phenotypes in the supposed triple mutants.

### Spike

**Table:** 

Keywords to find descriptions of mutants in the International Database for Barley Genes and Barley Genetic Stocks (bgs.nordgen.org):
Dense spike: compact spike, dense spike, dsp, erectoides, ert, lesser rachis internode number, lin, pyr, pyramidatum, pyramid shaped spike, short spike, zeo, zeocriton
Elongated spike: abr, accordion basal rachis internode, elongated basal rachis internode, lax, laxatum, lax spike, lbi, long basal rachis internodes, long spike, rac, rachisextensum, weak rachisextensum
Wilting spike: accordion rachis, accordionrachis, acr
Irregular spikes: abnormal spikes, aborted spike, absence of lower laterals, als, asp, branched spike, brc, com, compositum, crl, curled lateral spikelet, def, deformed spike, double seeds, double kernel, dub, extra central spikelet, extra floret, fertile rachilla, flo, hanging spike, irregular spikelet development, irregular spikes, lab, labile, nod, nodding spike, opposite spikelets, ops, rattail spike, rtt, snb, subnodal bract, variable rachis internode length, viv, viviparoides
Strength of spike: brittle rachis at maturity, btr, weak spikelet attachment, wsa

In addition to the many row-type mutants, a rich variety of other spike mutants have been isolated. Many of the mutations have pleiotropic effects. For example, dense spike mutants can also exhibit a reduction in coleoptile elongation, plant height and grain length. Irregular spike mutants are a very diverse group where asymmetric developmental mutants have been mixed with mutants in which various components of the spike develop abnormally.

#### Dense spike mutants

**Fig. 5:**
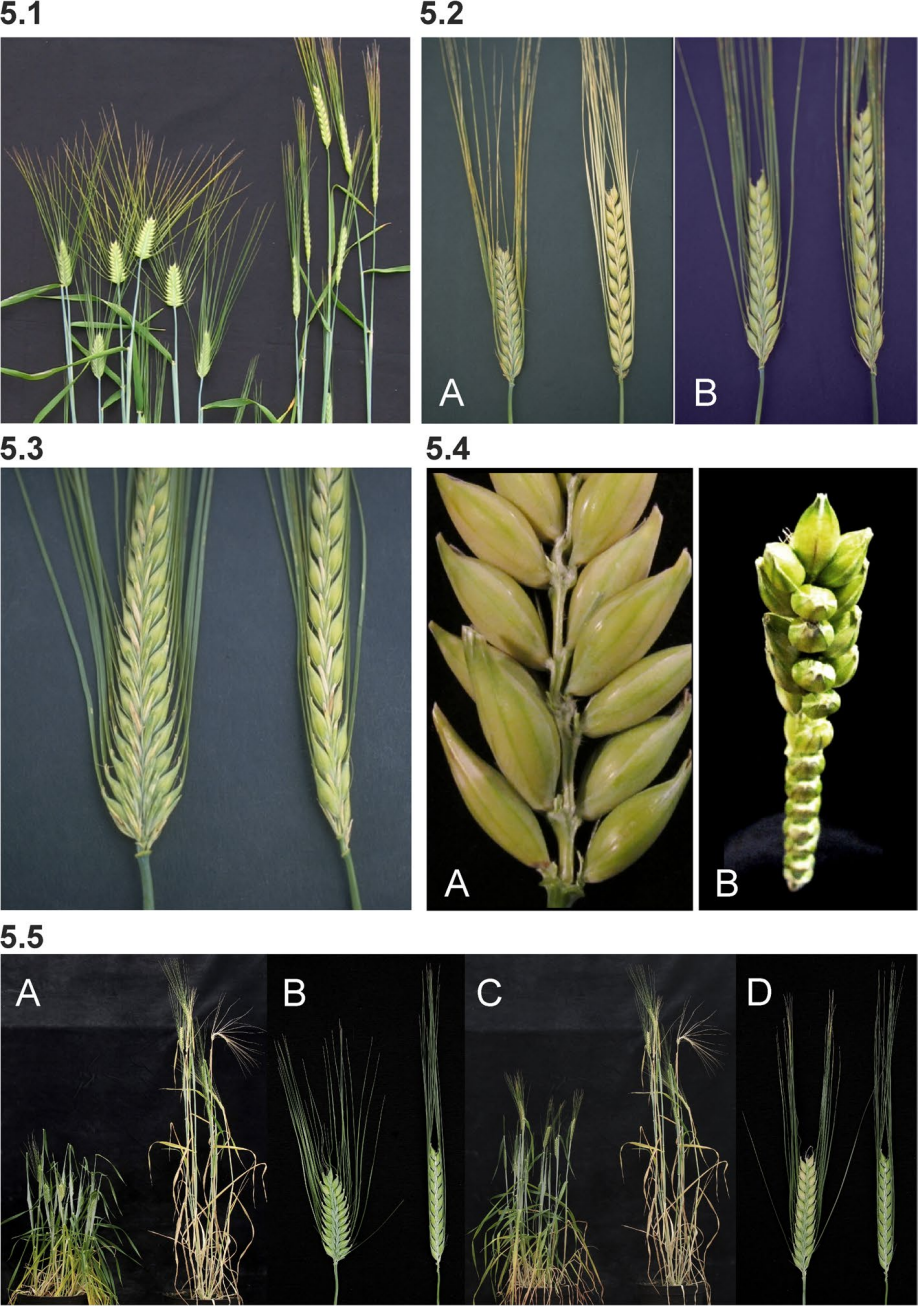
**5.1** Dense spike mutants often show a compact and wide spike with their awns protruding from the longitudinal axis of the plants. Mutant Pyramidatum 1 (*pyr1.i*) to the left compared to Bowman. **5.2** Less drastic dense spike mutants. **A.** Mutant Erectoides-a (*ert-a.6*) to the left compared to Bowman. **B.** Mutant *ert-k.32* to the left compared to Bowman. **5.3** The barley Erectoides-c mutants display a pyramid-shaped spike phenotype due to shorter distance between the rachis internodes at the lower part of the spike. Mutant *ert-c.1* to the left compared to Bowman. **5.4** Spikes of barley mutant Erectoides-m (*ert-m.330*) where awns and the lateral flowers have been removed. **A.** The rachis internode distances are irregular and the rachis nodes can sit more or less opposite to each other. **B.** The irregular rachis internode distance is especially pronounced in the top of the spike, which imposes twists of the spike. **5.5** Zeocriton mutants. **A and B.** *Zeo1.a* to the left compared to Bowman. **C and D.** *Zeo2.c* to the left compared to Bowman

Dense spike mutants have been isolated by several research groups and plant breeders starting in the 1920s, which explains the rich diversity of names (Dense spike, Erectoides, Pyramidatum, Short spike, Zeocriton) even though mutant phenotypes are relatively similar. Dense spikes are caused by a decreased distance between rachis nodes (short rachis internodes), which forces the seeds and their awns to protrude at wider angles from the longitudinal axis of the spike. Thus, spikes of typical dense spike mutants appear to be short and wide compared to normal spikes. The drastic mutants are easily spotted in mutant populations and their strong dense spike phenotypes are often accompanied by shorter culms (Fig. 5 (5.1)). Less drastic mutants, as *ert-a* and *ert-k* (Fig. 5 (5.2)) can be challenging to distinguish from their mother cultivars – especially under greenhouse conditions. The *ert-k.32* mutant was isolated in 1947 following X-ray treatment of Bonus and released as the cultivar Pallas in 1958. This was the first induced barley mutant to be released as a cultivar [16]. Pallas was a high-yielding cultivar known for its resistance to lodging. Pallas was further used in crosses to generate other cultivars such as Hellas (released in 1967), Visir (1970) and Senat (1974) [45, 60]. Therefore, the *ert-k.32* allele may exist in many of today’s elite European cultivars.

Spikelet density can also vary within the spike so that the distance between the rachis nodes is shorter at the bottom of the spike. This is the case in the *ert-c* mutants which have an obvious pyramid-shaped spike architecture (Fig. 5 (5.3)). In some cases, e.g. *ert-m*, the distances between nodes are irregular and some of the nodes can sit nearly opposite to each other (Fig. 5 (5.4)) [61].

Zeocriton mutants (Fig. 5 (5.5)) show an incomplete dominance in the case of *zeo1* and dominance in case of *zeo2* and *zeo3* [51]. Mutants in *zeo1* are allelic to the *ert-r* mutants [62, 63]. The incomplete dominance of *zeo1*/*ert-r* mutants make it possible to distinguish homozygous mutants from heterozygotes in segregating populations. Homozygous mutants are about two third normal height with excellent vigor. The glumes associated with lateral spikelets are three to four times larger than normal. Lodicule size is reduced [64, 65]. Heterozygotes are intermediate in plant height, have slightly more lax spikes, and have normal glumes in lateral spikelets [65]. Mutants in *zeo1* have also been called “Kurz und dicht”, *Knd* [66], and *zeo2* and *zeo3* mutants have both been reported as *Mo1* [67].

In Lesser internode number (*lin1*) mutants, the average number of fertile rachis nodes per spike is reduced by 20 to 40% [68, 69]. The average internode number was 15.3 in Triple Bearded Mariout and 22.9 in Spartan [68]. In some six-rowed cultivars such as Morex, the *lin1.a*-related reduction in rachis internodes is less obvious [42].

#### Mutants with elongated spikes

**Fig. 6:**
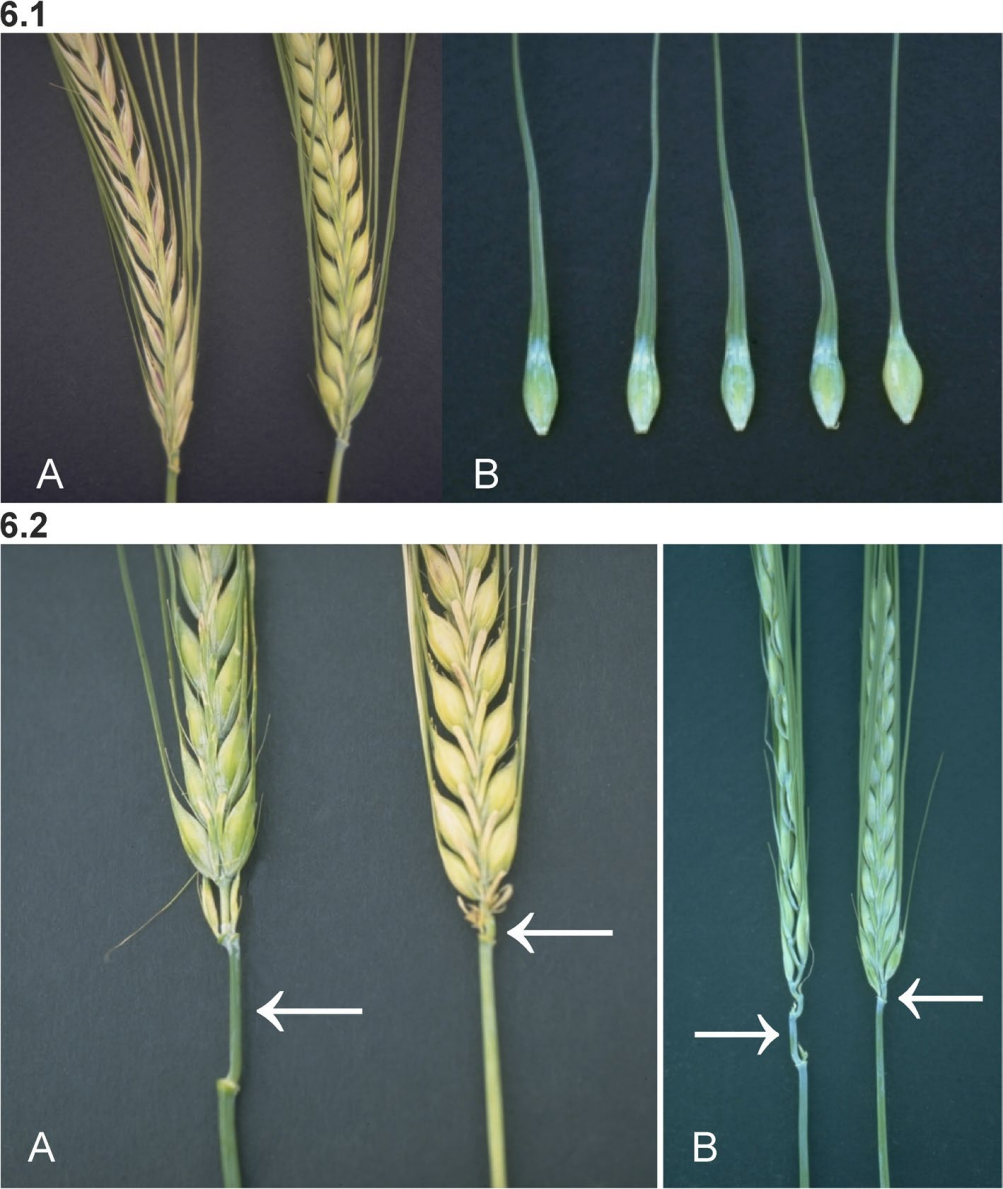
**6.1 A.** Spike of Laxatum-a (*lax-a.8*) at early maturity compared with normal Bowman. In *lax* mutants the rachis internodes are typically 10 to 20% longer than in their corresponding mother cultivars. Combined with thin and small kernels this results in a very sparse spike. The exposed caryopses are shown in the kernels of the *lax-a.8* mutant. **B.** The awns of *lax* mutants have a very wide base, without a distinct notch in the lemma attachment region. Four kernels of *lax-c.21* to the left compared to Bowman. **6.2 A.** Mutant Long basal rachis internode 3 (*lbi3.c*) in a Bowman genetic background to the left with typical elongated basal rachis internode, which is approximately ten times longer than that of Bowman (right). The *lbi3.c* mutation was originally isolated from the six-rowed cultivar Montcalm. In a Montcalm genetic background the basal rachis internode can be more than 10 cm [71]. **B.** The slightly curled or wavy basal rachis internode of Accordion rachis 1 (*acr1.a*) to the left compared to Bowman. The arrows point at the basal rachis internodes

The distance between rachis nodes in the spike can be longer than normal and such mutant plants show an opposite phenotype to dense spike mutants. In Laxatum (*lax*) mutants, the rachis internodes are typically 10 to 20% longer than in their corresponding mother cultivar Bonus (Fig. 6 (6.1)). Dense spike mutants are often accompanied by shorter culms, which therefore could suggest that *lax* mutants would have taller culms. This is not the case since *lax* mutants at many loci are shorter than normal. Kernels are often thin and small, and the yield can be as low as 10% in the case of *lax-b* compared to the corresponding mother cultivar. The caryopses are exposed between the lemma and palea in *lax-a* and *lax-c* mutants (Fig. 6 (6.1)). The awns of some *lax* mutants have a very wide base, without a distinct notch in the lemma attachment region (Fig. 6 (6.1)). The *lax-a* mutants have five anthers; the lodicules are replaced by stamens. In *lax-c* mutants, the awns and the basal rachis internode are slightly shortened [70].

The spike and the culm are joined at the node from which the collar develops. In Long basal rachis internodes (*lbi*) mutants, the basal (first) internode of the rachis is elongated. Mutant *lbi3*, derived from the cultivar Montcalm, has a marked elongation and weakness of the basal rachis node, which can be 10 to 13 cm long in some tillers. The spike hangs vertically downward from the collar as it emerges from the sheath and is often broken off by the wind. When not broken off, spikes have normal fertility, contain well-filled grains, and show normal maturity [71]. Expression of the *lbi3.c* allele in a Bowman-derived line is limited to a slight elongation of the basal rachis internode and a slightly lax spike (Fig. 6 (6.2)). The *ert-i* mutants isolated in cultivar Bonus were found to be allelic to *lbi2* [72]. The *ert-i* mutants in Bonus have an erect, semi-compact spike, an elongated (2 to 4 cm) basal rachis internode, and reduced plant height (3/4 normal) [65]. Expression of the *lbi1.a* phenotype is commonly more pronounced in the genetic background of six-rowed cultivars compared to two-rowed cultivars. In many environments, plants of the Bowman backcross-derived line BW471 (*lbi1.a*) were about 10% taller than Bowman and had longer peduncles. Rachis internodes were slightly longer, and spikes often had one or two more fertile rachis nodes. Kernels of BW471 plants were often slightly longer and heavier than those of Bowman [73]. In Accordion basal rachis internode 1 (*abr1*) mutants, the elongated basal rachis internode is slightly curled or wavy (Fig. 6 (6.2)).

#### Undulating spikes

**Fig. 7:**
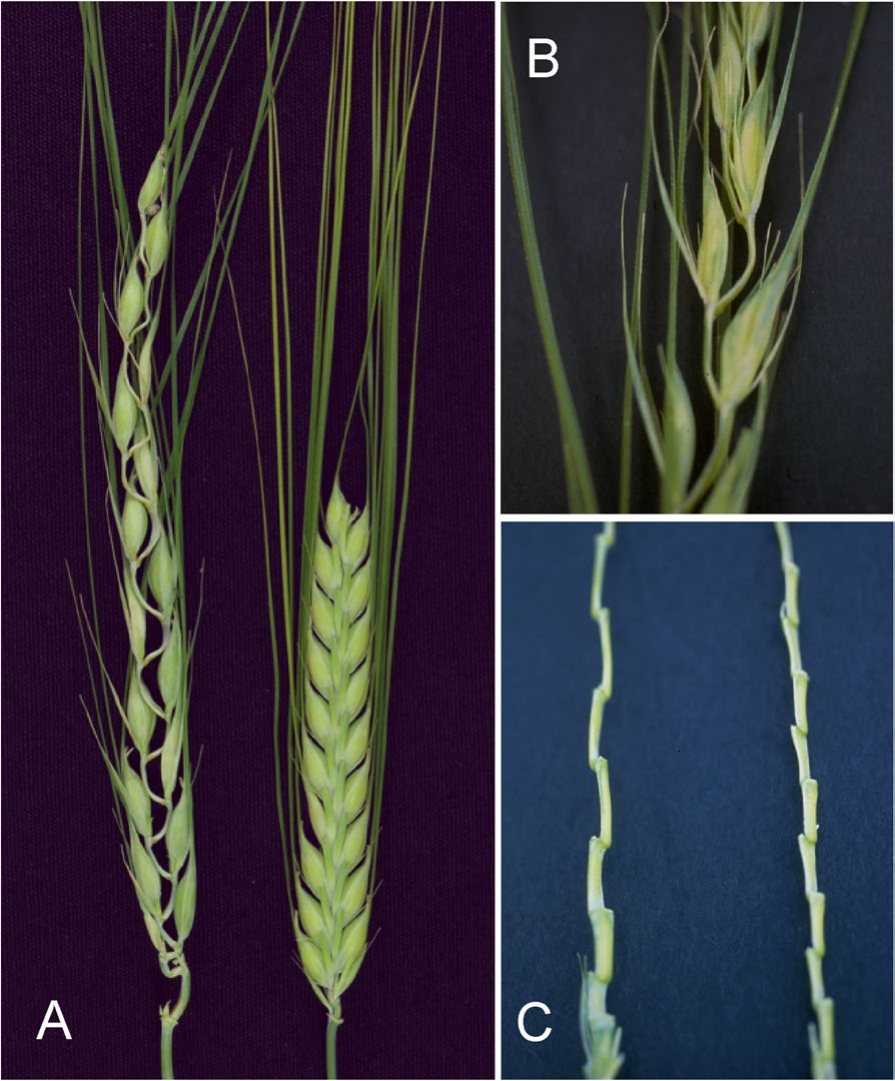
Accordion rachis mutants have greatly elongated rachis internodes causing a wavy or undulating form. All photos show mutant *acr1.a*. **A.** Mutant to the left, Bowman to the right. **B.** Elongation of the rachis internodes is associated with slightly elongated glumes and the deficiens-like spike phenotype. **C.** Spikelets have been removed to show the wavy form of the spike

Spikes with greatly elongated rachis internodes can obtain a curled, wavy or undulating form. These mutants are grouped as Accordion rachis (*acr*) mutants (Fig. 7). Rachis internodes are greatly elongated and often bent or pleated as the spike emerges from the sheath of the flag leaf [42]. The line ACBV89B229, developed by R.I. Wolfe to maximize rachis internode length, exhibits extreme elongation of rachis internodes, rachis internode length values up to 7.7 mm, and occasionally trapping of the spike tip in the sheath of the flag leaf [51]. Elongation of the rachis internodes is associated with slightly elongated outer glumes and the Deficiens (*Vrs1.t*) spike phenotype. Two modifiers, *acr2* and *acr3*, caused variable expression of the accordion trait in different genetic backgrounds. In crosses to Bowman, segregation for *acr1* fits a three gene model based on DNA segments retained in the Bowman backcross-derived lines BW009 (*acr1.a*) and BW439 (*lax.ao*) [23]. Although the *acr1* gene is apparently associated with the Deficiens spike type, the retained centromeric segment of 2H does not overlap the Six-rowed spike 1 (*vrs1*) locus [23]. A pericentric inversion cannot be eliminated as a possibility. Plants of the BW009 and BW439 lines were about 3/4 of normal height and peduncles were about 1/2 of normal length. The number of fertile rachis nodes was reduced by about three and heading was delayed by up to four days. The kernels appeared thinner and weighed about 10% less. Test weights were low and grain yield was about 3/4 of normal [51].

#### Mutants with irregular spikes

**Fig. 8:**
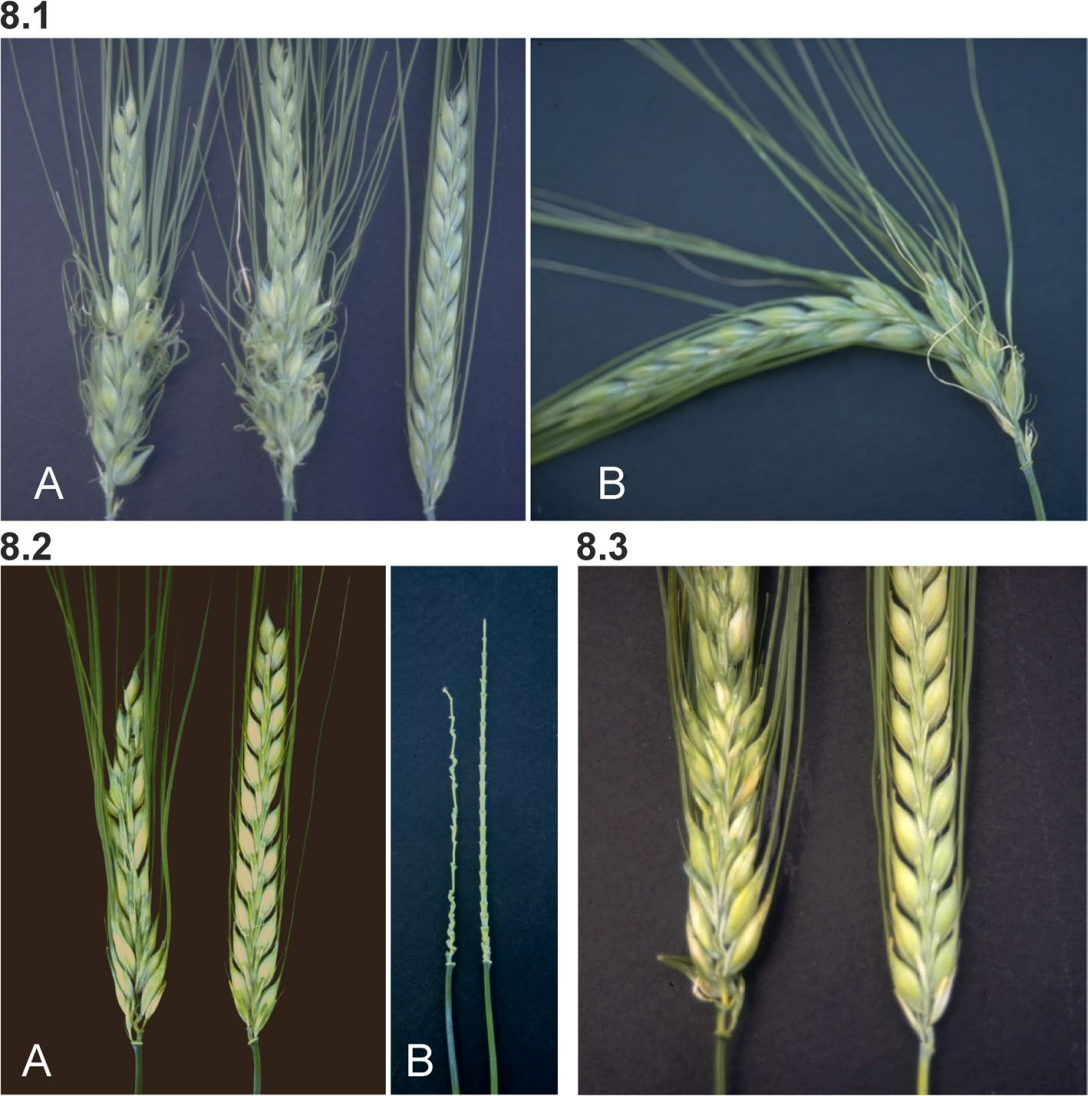
**8.1 A.** Spikes of Compositum 1 (*com1.a*) mutant to the left compared to cultivar Bowman. **B.** A single *com1.a* spike that has been bent to better visualize the branches of a few small spikes from the rachis at the lower part of the spike. Several thread-like awns are protruding from the cluster of small spikelets. **8.2** Mutant Opposite spikelets 1 (*ops1.3*) to the left in each photo, displays variable lengths of rachis internodes, which causes an irregular arrangement of spikelets in the spike. **A.** Spike of *ops1.3* compared to Bowman. **B.** Spikelets have been removed in order to view the rachis nodes and internodes. **8.3** Lateral spikelets at the base of the spike fail to develop or are partially developed in Absent lower laterals 1 (*als1.a*) mutants (left) compared to Bowman (right)

Irregular spike mutants have lost their two-fold symmetry and are thus less esthetic. In Compositum (*com*) and Branched (*brc*) mutants this is caused by branches of additional small spikes protruding from the lower part of the rachis [74, 75]. Awns, which vary from normal to thread-like, and protruding branches are bent in various directions because of "packing" problems in the sheath of the flag leaf (Fig. 8 (8.1)). An asymmetric spike phenotype is also obvious in Opposite spikelets (*ops*) mutants in which a variable length of the rachis internodes causes an irregular arrangement of spikelets in the spike (Fig. 8 (8.2)). A variable rachis internode length is also observed in *ert-m* mutants [61], which could indicate some functional relationship between the *ert-m* and *ops1* gene products. The *ert-m* and *ops1* loci are both located on chromosome 7H but not in the same region, whereas *ops2* and *ops3* are mapped to chromosome 5H [23].

In Absent lower laterals 1 (*als1*) the lateral spikelets at the base of the spike fail to develop or are partially developed (Fig. 8 (8.3)). Tillers are large, coarse, and stiff, and only one or two tillers are produced in the six-rowed stock [71]. This makes the *als1.a* plants resemble those of Uniculm 2 (*cul2*) mutants. Plants of the Bowman backcross-derived *als1.a* line commonly produce 3 to 4 tillers with short, malformed spikes (irregular placement of central and lateral spikelets), and seed yields are very low [76]. The *als1.a* plants produced primary tillers, but secondary tillers were not formed [76]. Other morphological differences between the Bowman backcross-derived line and Bowman included longer awns, 17 vs. 12 cm, and 3 to 5 more kernels per spike.

**Fig. 9:**
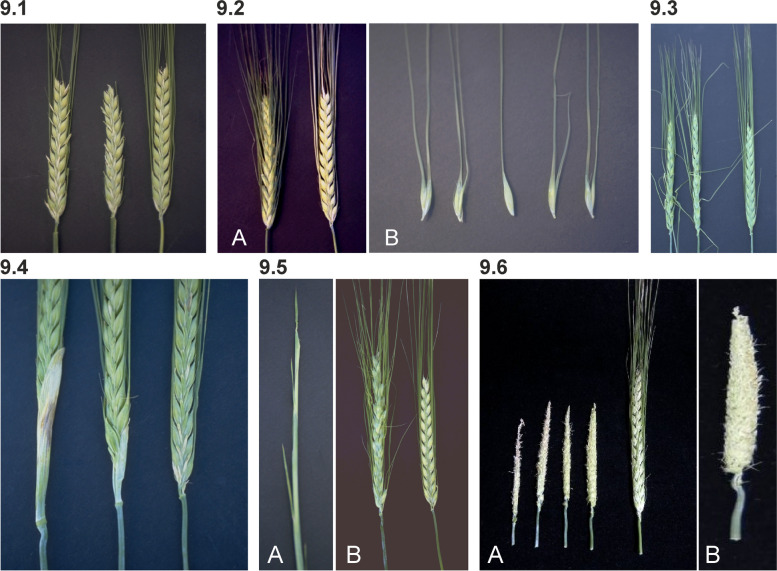
**9.1** Two Extra floret-a (*flo-a.3*) spikes compared to Bowman (right). The awns have been removed from the central spike to better view the extra bracts. **9.2 A.** Mutant Subnodal bract 1 (*snb1.a*) to the left, Bowman to the right. **B.** Four single spikelets of *snb1.a* flanking a spikelet of Bowman in the middle. **9.3** Two spikes of Curly lateral 1 (*crl1.a*) to the left with bent awns compared to Bowman. The awns are approximately 20% shorter than those of Bowman. **9.4** Two spikes of Leafy bract 1 (*Lfb1.a*) with different expressions to the left compared with normal Bowman. **9.5 A.** Top part of a *vir-a.5* mutant tiller with a Viviparoides phenotype remaining vegetative. **B.** In the near-isogenic line BW896 carrying the *viv-a.5* mutation, spikes are formed on most tillers (left). Bowman (right). **9.6 A.** Four spikes of Rattail spike 1 (*rtt1.a*) with numerous immature spikelets compared to cultivar Bowman (right). **B.** A single *rtt1.a* spike

Extra reproductive bracts develop occasionally at the base of the central spikelet on the abaxial side in Extra floret-a (*flo-a*) mutants (Fig. 9 (9.1)). Formation of the extra bracts is most common in the central portion of the spike. The bracts will rarely form another spikelet. In Subnodal bract 1 (*snb1*) mutant, a glume-like or stick-like bract arises immediately under the node-base (below and between the glumes) of the central spikelets (Fig. 9 (9.2)). The bracts are present at only a few nodes and are arranged below random central spikelets. Not all spikes of mutant plants have extra bracts [77].

The phenotype of Curly lateral 1 (*crl1*) is preferably seen in six-rowed barley lines where the awns on lateral spikelets are curly or wavy, and lateral spikelets may be malformed and partially sterile. Central spikelets may occasionally have a twisted awn [77]. The Curly lateral trait is not expressed in all tillers [77]. In the two-rowed Bowman backcross-derived line for *crl1.a*, BW194 [23], awns seemed thinner and the awns of some spikelets were bent at odd angles (Fig. 9 (9.3)).

Leafy bract 1 (*Lfb1.a*) is dominant and causes a leaf-like bract at the collar below the spike (Fig. 9 (9.4)). The size of the leaf-like bract may vary from almost absent to 5 cm or longer [77, 78]. Tiller to tiller variation in bract size occurs and the bract is often larger in cultivars having six-rowed spikes.

In the Double seed 1 (*dub1*) mutant, modification of the top of the spike is distinctive and occurs on all tillers. The tip of the spike is compacted, and a few spikelets form two and three fertile florets adjacent to each other. The double spikelets have fused lemmas, and paleas often enclose the part of two, occasionally more, flowers, which can have six anthers and two ovaries. The tip of the spike appears phenotypically similar to those of *int-m* mutants.

Mutant plants of Aborted spike 1 (*asp1*) appear normal, except the spike is rudimentary or missing. Homozygous *asp1.a* plants occasionally form spikes with a few spikelets, but the spikelets are mostly sterile. The stock must be maintained as a heterozygote [55]. Also in Viviparoides (*viv*) mutants the tillers often remain vegetative and fail to produce reproductive structures [79]. The apex of the tillers remains vegetative as the culm elongates and only occasionally a short, malformed spike is formed. It was observed that only a few tillers exhibit the typical Viviparoides phenotype in the Bowman backcross-derived line BW896 (*viv-a.5*) (Fig. 9 (9.5)).

Spikes of Rattail spike 1 (*rtt1*) are highly spectacular with numerous immature spikelets and complete sterility (Fig. 9 (9.6)). The arrangement of the bracts in the spikelets suggests that they contain numerous florets. Because the homozygous recessive plant is completely sterile, the stock must be maintained in heterozygous condition [80]. A semidominant interaction between *rtt1.a* and one version of the normal allele (*Rtt1.b* or *Rt'*) in Okaiku 3 has been reported [81].

#### Strength of the spike

The domestication process of plants and animals is inevitably accompanied with genetic changes. One of the earliest events during barley domestication was the loss of brittleness or disarticulation at the rachis nodes [82]. In the wild form of barley, *Hordeum vulgare* ssp. *spontaneum*, rachis segments are brittle at maturity due to thin cell walls in the middle of the node. This contrasts with domesticated barley, *Hordeum vulgare* ssp. *vulgare*, where rachis segments are strongly fused and non-brittle, thus preventing disarticulation of the rachis prior harvest. Two mutants have been described – one in the Brittle and tough rachis 1 locus (*btr1.a*) and one in the Brittle and tough rachis 2 locus (*btr2.b*). The two loci are closely linked [83]. Domesticated barley with the *btr1.a* allele have a dominant allele at the *btr2* locus and all barley plants with the *btr2.b* allele have a dominant allele at the *btr1* locus. In a cross between a *btr1.a* plant (genotype *btr1.a*/*btr1.a Btr2/Btr2*) and a *btr2.b* plant (genotype *Btr1*/*Btr1 btr2.b*/*btr2.b*), all F1 progenies have a brittle rachis. The segregation ratio in the F2 generation is 1 brittle:1 tough rachis [83]. The segregation pattern is explained by the close linkage of the *btr1* and *btr2* loci, which are now known to be separated from one another by only 88 kb in the cultivar Morex and 118 kb in cultivar Haruna Nijo [82]. The *btr1.a* and *btr2.b* mutations have been suggested to represent two independent domestication events, which occurred in the southern and northern regions of the Levant [82]. The *btr1.a* allele is today widely distributed in cultivars in Europe and the Middle East, whereas *btr2.b* is most frequent in material from East Asia and North Africa [82]. The detachment of kernels from the spike in two-rowed barley is a dominant trait, which causes rachilla disarticulation between the glumes and the lemma. The recessive allele at Weak spikelet attachment 1 (*wsa1*) is present in hulless barley landraces where detachment does not occur [84].

### Spikelet

**Table:** 

Keywords to find descriptions of mutants in the International Database for Barley Genes and Barley Genetic Stocks (bgs.nordgen.org):
Glume: bra, bracteatum, elongated glume, elongated outer glume, eog, gillette, lep, lga, long glume awn, macrolepis, many glumes on lateral spikelets, semibracteatum, short rachilla hair, srh, third outer glume, trd
Lemma: acute lemma on lateral spikelets, branched awn, gth, hairs on lemma nerves, hln, pointed lateral spikelet, sci, scirpoides spike, sls, small lateral spikelet, toothed lemma, triaristatum, triple awned lemma, trp
Palea: adp, awned palea

The spikelet consists of one or more florets flanked by two small glumes having a hair-like structure. In grasses, the spikelet is attached to the rachis (the culm of the spike) via the pedicel or first internode of the rachilla (secondary rachis). However, in barley subsequent elongation of the rachilla is strongly reduced to a short rod-shaped appendix. The floret contains the lodicules and reproductive organs of the flower (the pistil with a feather-like stigma and an ovary, and three stamens with pollen-containing anthers) surrounded by the lemma and the palea. In barley, the lemma commonly elongates to form a characteristic awn. In contrast to the many structural features forming the spikelet relatively few mutants have been isolated affecting floret morphology. The reason might be the small sizes of these structures, which can be tedious to screen for in mutant populations grown in field.

#### Glume mutants

**Fig. 10:**
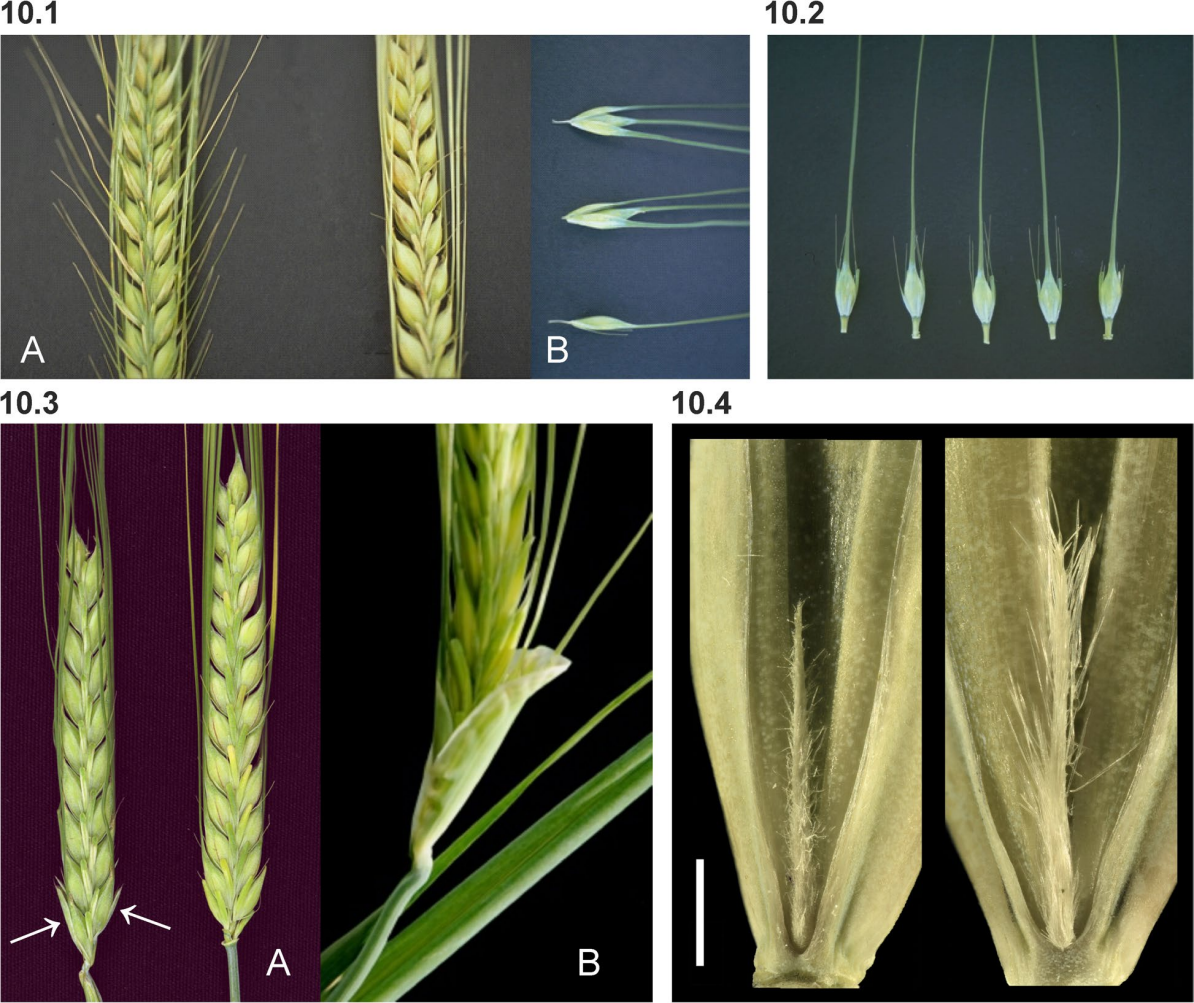
**10.1** In Elongated outer glume 1 (*eog1*) mutants, the glumes have been enlarged and display an awn that can be as long as the awn of the lemma in some mutant alleles. **A.** *eog1.a* to the left, Bowman to the right. **B.** Two spikelets of *eog1.a* (top) and one of Bowman (below). The glumes, subtending the spikelet with the lemma awn, are both wider and taller in the mutant. **10.2** Spikelets of Long glume awn 1 (*Lga1.a*) compared to Bowman (right). The dominant variant causes elongated glume awns, which are much longer than the kernel. **10.3 A.** Spikes of barley Bracteatum-c (*bra-c.1*) compared to Bowman (right). The arrows indicate the third outer glume at the two lowest spikelets. The glume-like structure associated with the lowest spikelets are always the largest and they become progressively smaller toward the top of the spike. **B.** A close-up of the lower part of a spike of Third outer glume 1 (*trd1.b*) showing the pronounced glume-like structure at the base of the spike. **10.4 **Short and long rachilla hairs in cultivar Morex (left) and Barke (right), respectively. Scale bar 1 mm. Image kindly provided by Twan Rutten, IPK Gatersleben

Among the barley spikelet mutants, the only locus with multiple alleles is Elongated outer glume 1 (*eog1*). The number of alleles increased considerably when it was found that *eog1.a* is allelic to Macrolepis mutants *lep-e* [85, 86]. The more than 70 recessive alleles (bgs.nordgen.org) control increased size of the glumes (Fig. 10 (10.1)). Glume width of *eog1* mutants varies from 2.5 to 4.0 mm depending on genetic background and specific allele [86, 87]. Glume awn length varies from awnless to nearly as long as the lemma awn (92 mm) [86–88]. The size of the glume can range from less than twice the normal width in CIho 14955 to lemma-like glumes in Triple Bearded Mariout. Kernels of the Bowman backcross-derived lines for *eog1.a* (BW299) and *eog1.c* (BW300) were slightly larger than those of Bowman.

A single mutant is known at the Long glume awn 1 (*lga1*) locus. This semi-dominant mutation causes elongated glume awns, which are much longer than the kernel (Fig. 10 (10.2)). Heterozygotes have a glume awn of intermediate length. In the short glume awn phenotype (*lga1.b*), the glume plus its awn is about the same length as the kernel [89, 90]. Tsuchiya [87] reported that the glume awn length is 4 to 6 mm for the short type (*lga1.b*) and 11 to 13 mm for the long type (*Lga1.a*). Recessive alleles at the *eog1* locus produce an array of glume sizes, some of which have glumes phenotypically similar to those associated with the dominant *Lga1.a* allele. However, large glumes controlled by *eog1* alleles are wider than normal and show a recessive inheritance pattern.

An additional glume-like structure is associated with recessive mutations in the three loci: Third outer glume 1 (*trd1*), Bracteatum-a (*bra-a*) and Bracteatum-d (*bra-d*). The additional bracts are located outside the two ordinary glumes of the central spikelets and are attached to rachis nodes. The bract subtending the lowest spikelet is always the largest, embracing in some cases about one-half the spike. Bracts become progressively smaller towards the top of the spike (Fig. 10 (10.3)). Development of the bracts is poor in the Bowman backcross-derived line BW067 (*bra-a.001*) (bgs.nordgen.org). Allelism was found between *trd1* and *bra-c.1* [86]. There are four *trd1* mutants and seven *bra-c* (bgs.nordgen.org). Pozzi et al. [91] suggested that *bra-d.7* is allelic to *trd1* or is located near the *trd1* locus. Allelism studies, however, did not support allelism of *bra-d.7* and *trd1* [92].

The rachilla in barley is strongly reduced into a rudimentary short rod-shaped appendix (Fig. 10 (10.4)). The hairs on the rachilla are normally long and unicellular. The recessive *srh1.a* allele results in short rachilla hairs that are multicellular and branched [93]. The *srh1.a* allele is also associated with short pubescent hairs on the glumes and rachis margins [88, 94, 95]. In the case of the Stubble 1 (*stb1*) or Gillett mutant, rachilla hairs are missing [96].

#### Lemma mutants

Different types of lemma mutants are available: Triple awned lemma 1 (*trp1.a*), Hairs on lemma nerves 1 (*Hln1.a*) and Tooth lemma 1 (*Gth1.a*). The latter two are dominant whereas *trp1.a* is recessive. A fourth type of lemma mutant (Leafy lemma 1, *lel1.a*), in which the lemma is similar to that of a miniature grass leaf, was isolated in Italy [97].

**Fig. 11:**
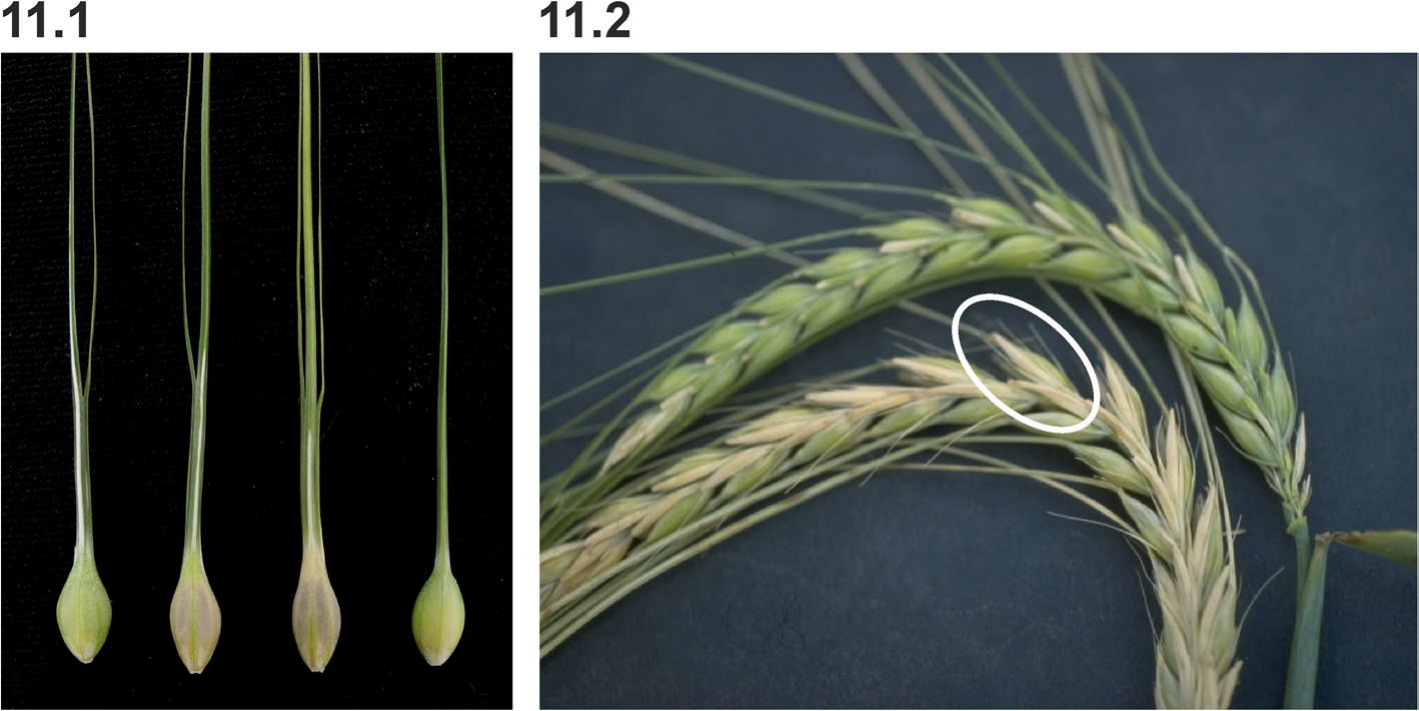
**11.1 **Three Triple awned lemma 1 (*trp1.a*) mutant grains to the left compared to Bowman. In *trp1.a*, the awn extending from the lemma of the central spikelet forks to form one normal central awn and one or two shorter lateral appendages. **11.2 **A spike of Hairs on lemma nerves 1 (*Hln1.a*) (bottom) compared to Bowman (top). *Hln1.a* causes additional hairs of 1 to 2 mm on the lateral veins of the lemma (encircled)

Plants carrying *trp1.a* may have three awns on the lemma instead of one, but expression is variable [98]. The awn or hood extending from the lemma of the central spikelet forks to form one normal central awn and one or two shorter lateral appendages (Fig. 11 (11.1)). Expression of triple awn trait in the Bowman backcross-derived line BW881 is reduced to an occasional branch in the basal part of the awn. However, when plants are grown under heat stress a stronger expression of the *trp1.a* trait was observed (bgs.nordgen.org).

The *Gth1.a* allele causes formation of large teeth or barbs on the upper part of lateral lemma veins. The barbs are easiest to see on green spikelets. This trait may be difficult to study because three sizes of teeth were reported, including one that could be seen only with magnification [99]. It was further reported that two genes control the presence and absence of large teeth, and one or two other genes are responsible for less developed teeth [99]. Segregation for only two genes was reported in another study [100]. *Gth1.a* is present in the cultivar Bowman. A Bowman backcross-line (BW413) carries the *gth1.b* allele, which is the allele that is present in most western world two-rowed cultivars [23]. Besides barbs on the lateral lemma veins and slightly heavier kernels, no other agronomic or morphological differences were found between Bowman and BW413 [51].

In *Hln1.a* a few hairs of 1 to 2 mm are mixed with the ordinary teeth or barbs on the lateral nerves of the lemma (Fig. 11 (11.2)) [101]. Expression of the *Hln1.a* allele may be easier to observe as 1 mm hairs on the tip of sterile lateral flowers in two-rowed barley. The *hln1* gene is associated with a recessive short awn trait (2/3 of normal length). The Bowman backcross-derived line BW415 (*Hln1.a*) displays the hairs but is otherwise similar to Bowman except awns were half normal length and kernels were slightly heavier (bgs.nordgen.org).

#### Palea mutants

**Fig. 12:**
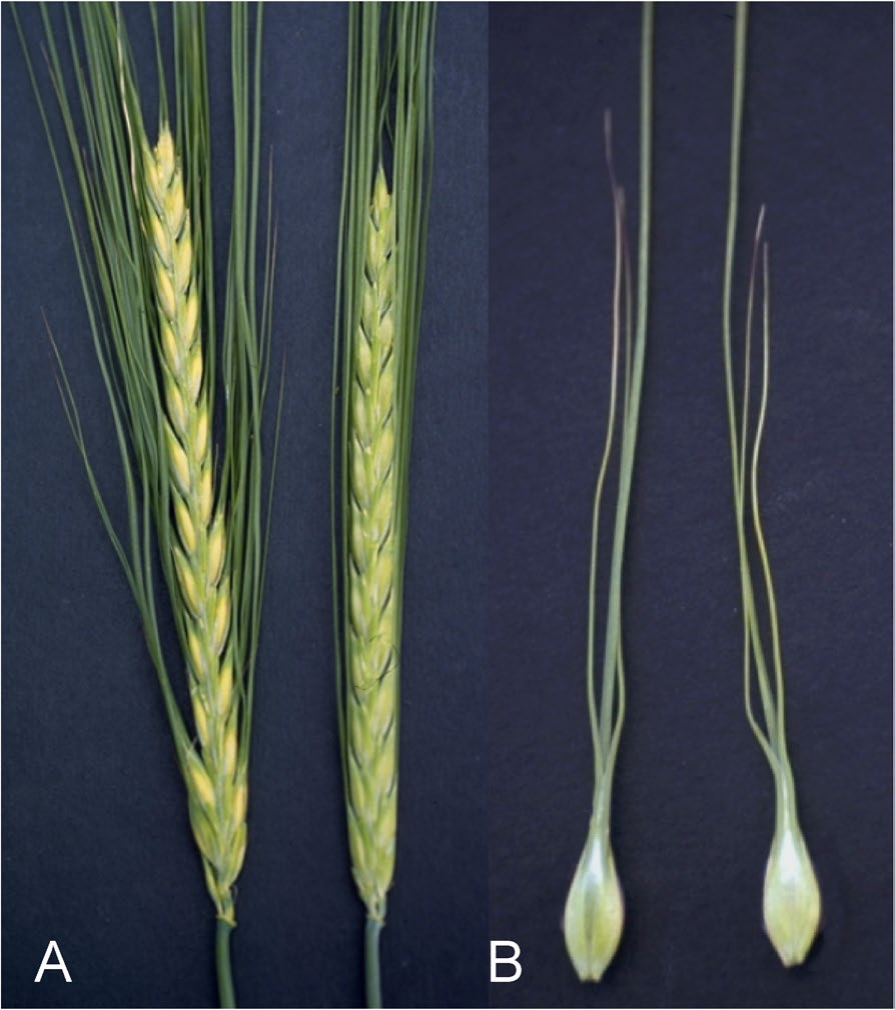
In Awned palea 1 (*adp1.a*) mutants the palea has two awns in addition to the awn protruding from the lemma. **A.** Mutant *adp1.a* to the left compared to Bowman. **B.** Two grains of *adp1.a*. The two awns of the palea are shorter than the awn of the lemma

The Awned palea 1 (*adp1.a*) mutant was isolated as a spontaneous mutant in an inbred line [102]. The mutant is partially female sterile with abnormal spikes. The palea is elongated to form two awns (Fig. 12) [91]. Pistils are often transformed into leafy buds and result in low female fertility and greatly reduced seed set [102]. Two of the anthers appear normal and the third is slightly deformed. Pollen fertility is good. Plants of the Bowman backcross-derived line for *adp1.a*, BW010, compared to Bowman plants produced spikes with slightly longer rachis internodes. Kernels of BW010 were slightly thinner and weighed 30% less. Split or bifurcated palea mutants in which the two bracts forming the palea fail to fuse were identified by Forster et al. [103] and studied by Yoshikawa et al. [104].

### Awn length and formation

**Table:** 

Keywords to find descriptions of mutants in the International Database for Barley Genes and Barley Genetic Stocks (bgs.nordgen.org):
Awn length: ari, breviaristatum, law, lks, long awn, short awn
Curly awn: caw, curly awn
Awn roughness: few barbs, raw, saw, smooth awn, soft awn
No or hooded awns: awnless, cal, calcaroides, hooded lemma, kap, lks, sbk, sca, short crooked awn, subjacent hood

The awn is a characteristic feature of barley. It has been defined as a linear extension of the vascular lemma tissue and may therefore be considered as an integral part of the barley floret [105]. The barley awns are often 10–20 cm in length. The awn extends from the lemma and is covered with barbs. This makes the awn rough when fingers slide down the awn from top to bottom. Probably, the awns and the barbs help spread the seed as well as driving the kernel into the soil. During the maturation process, the plant eventually turns yellow and dry. The awns are normally the part of the barley plant that remains green last and it has been suggested that their photosynthetic activity support the final grain filling in the maturing seed [106].

#### Awn length mutants

**Fig. 13:**
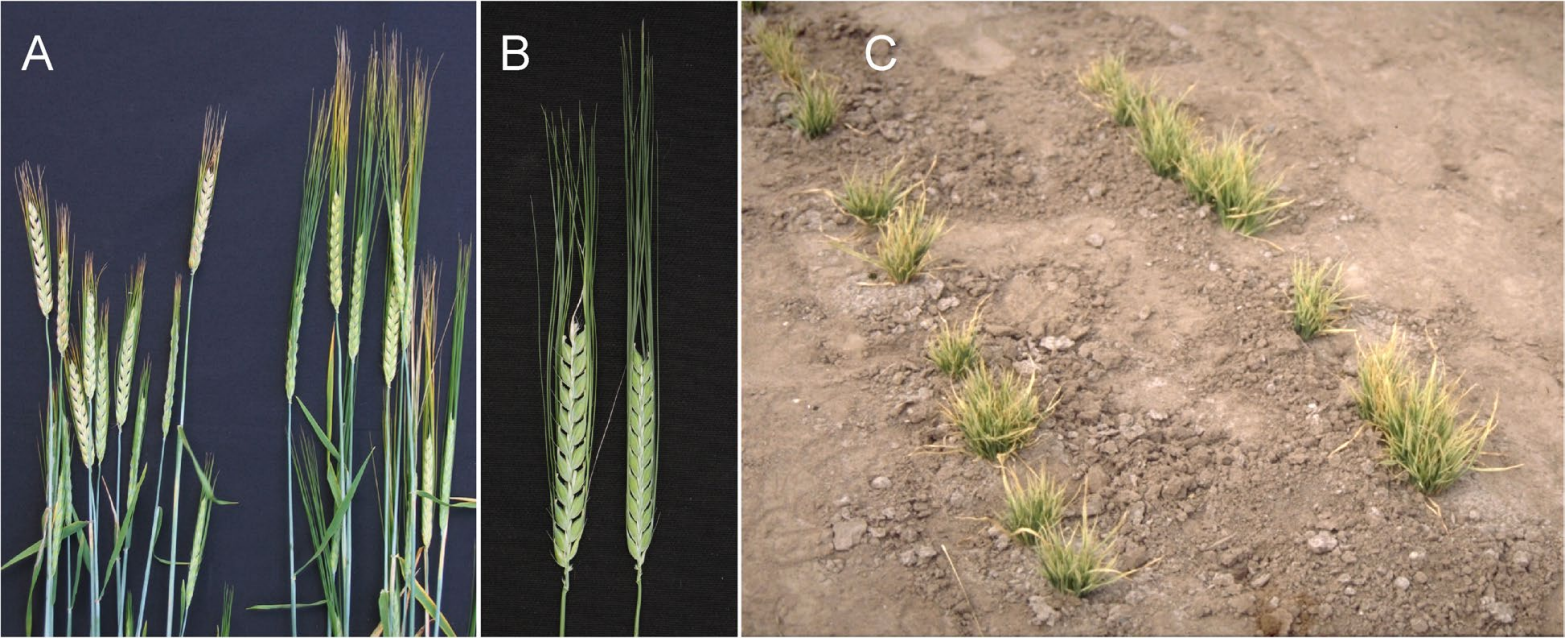
Three different short-awn mutants. **A.** Mutant Breviaristatum-n (*ari-n.45*) to the left compared to Bowman. **B.** Mutant *ari-a.8* to the left compared to Bowman. **C.** Field plot of *ari-g.18* showing its strong dwarf phenotype

Awn length is a phenotypic character easily observed in the field. This could explain why the short-awn mutant group Breviaristatum (*ari*) is one of the largest groups of phenotypic mutants besides dense spike Erectoides (*ert*), and waxless Eceriferum (*cer*) / Glossy sheath (*gsh*) mutants. Approximately 500 short-awn mutants are available (bgs.nordgen.org). Of those tested for allelism, including Awnless and Short awn (*lks*) mutants, more than 200 are distributed in 31 loci represented by 1 to 31 mutants each (Table 2) [29, 42]. Awn lengths in the Breviaristatum group are typically 1/4 to 5/6 of that of wild type (Fig. 13). In most, if not all, short-awn mutants, other phenotypic characters can be observed. Most striking is dwarfism ranging from strong dwarfism in *ari-g* mutants (Fig. 13) to almost none in *ari-k* and *ari-p* mutants. The seed yield is very low in *ari-g* mutants and these mutants must be kept in heterozygous stocks [42]. Other observed pleiotropic characters are reduced stigma hairs and partial female sterility in *lks2*/*ari-d* [107], curled peduncle in *ari-j*, dehiscent (breakage or detachment) awns at maturity in *ari-k*, and smaller, often globe-shaped kernels in many mutant lines [29, 42]. Recent analyses of *ari* and *lks* mutants have shown that they overlap with other mutant groups. The *ari-m* mutants are allelic to Brachytic 1 (*brh1*) mutants and deficient in the α-subunit of a heterotrimeric G-protein signaling complex [108]. Similarly, the *lks2* and *ari-d* mutants are allelic to Unbranched style 4 (*ubs4*) and orthologous to Arabidopsis *SHORT INTERNODES* (*SHI*) encoding a transcription factor [107]. Further, *ari-o* is allelic to the *brh14*, *brh16*, *ert-u* and *ert-zd* mutants and encodes a Δ5-sterol-Δ24-reductase (DIMINUTO) of the brassinosteroid biosynthetic pathway. The *ari-u* mutant is allelic to *brh3* and *ert-t* and encodes a brassinosteroid-6-oxidase in the same pathway [27]. A mutant at the *ari-e* locus, *ari-e.GP*, has been of great economic importance since it was released in the Scottish malt cultivar Golden Promise [109]. Similar to *ari-m*/*brh1*, *ari-e* is associated to a heterotrimeric G-protein since it encodes the γ-subunit of the signaling complex [109]. Interestingly, mutant alleles at the *ari-e* locus are associated with salt tolerance, lower accumulation of Na^+^ [110–112] and show relative insensitivity to gibberellic acid-3 [112]. A gene for slightly increased awn length was identified in Morex [113] and is likely present in Bowman and many other cultivars.

**Table 2:** The number of allelic mutants in 31 groups of short-awn mutants. All mutants are recessive with exception of *Ari-s.265*

Locus	No. of mutants
*ari-a*	27
*ari-b*	7
*lks5/ari-c*	31
*lks2/ari-d/ubs4*	30
*ari-e*	9
*ari-f*	27
*ari-g*	11
*ari-h*	2
*ari-i*	1
*ari-j*	3
*ari-k*	4
*brh2*/*ari-l*	7
*brh1*/*ari-m*	15
*ari-n*	11
*ari-o/brh14/brh16/ert-u/ert-zd*	8
*ari-p*	7
*ari-q*	3
*ari-r*	6
*ari-s*	1
*ari-t*	1
*ert-t/ari-u/brh3*	7
*ari-v*	1
*ari-w*	1
*ari-x*	1
*ari-y*	1
*ari-z*	1
*ari-za*	1
*lks6*	1
*lks7*	1
*lks8*	1
*lks9*	1

#### Curly awn mutants

In Curly awn (*caw*) mutants the lemmas and awns are coiled or strongly twisted. 73 mutants have been isolated by mutagenic treatment of mostly Bonus, Foma and Kristina between 1950 and 1978. These mutants have not been studied and no diallelic analyses have been performed. Therefore, it is not known how many loci these mutants represent.

#### Awn roughness

**Fig. 14:**
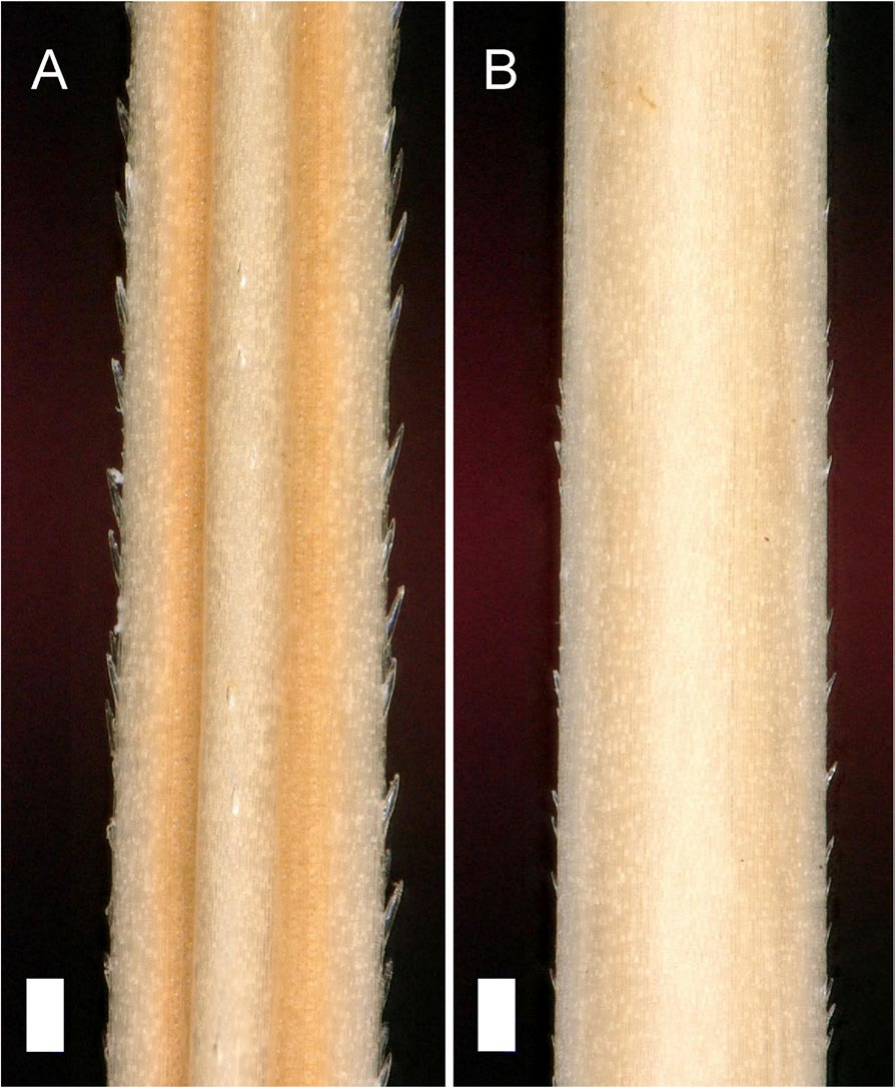
Awns of cultivars Barke (**A**) and Morex (**B**). Barke (*Raw1*/*Raw1*) and Morex (*raw1*/*raw1*) have rough and smooth awns, respectively. The white bars correspond to 100 μm. Image kindly provided by Twan Rutten, IPK Gatersleben

Barley awns commonly have barbs or teeth at the margins and the central vein. In Smooth awn 1 (*raw1*) mutants, the barbs at the margins are almost absent and the number of barbs at the vein are reduced (Fig. 14) [114]. There are at least three other *raw* loci reported in early literature. The *raw2* and *raw6* loci were reported to be slightly linked to *raw1* on the long arm of chromosome 5H [115]. Locus *raw5* is located on the long arm of chromosome 6H [116]. Based on a large genome-wide association study (GWAS) there is solid evidence for two genetic loci segregating in global barley diversity that are explaining the majority of the phenotypic variation in haptic assessment of the trait [117]; one is *raw1*. The second locus, on the short arm of 7H, was not linked to awn roughness before, which is not coinciding with any of the previously reported genetic loci. The study by Milner et al. [117], suggested that smoothness of the barley awn is controlled at least by two genes. In early reports it was mentioned that stigma hair formation may also be affected by mutations at the raw loci. Stigma hair formation is affected by the *raw* mutations. Homozygous *raw1* mutants showed a reduced number of stigma hairs and reduced seed set may occur in some heat and moisture stressed environments [118].

#### Mutants with no or hooded awns

**Fig. 15:**
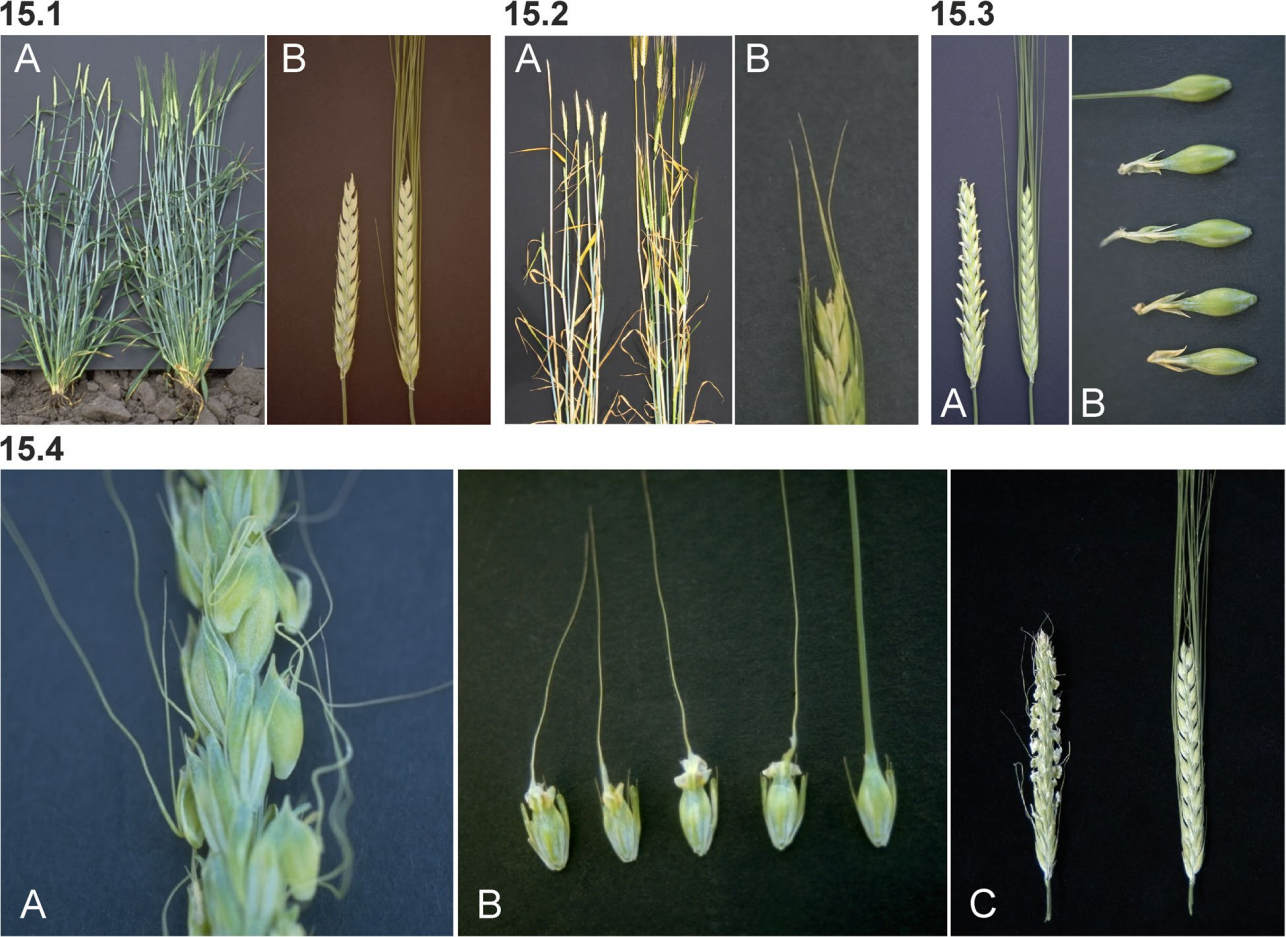
**15.1 A and B.** *Lks1.b* (Awnless 1) to the left compared to Bowman. **15.2 A.** Mutant Short crooked awn 1 (*sca1.a*) to the left compared to Bowman. **B.** Enlarged upper part of a spike of *sca1.a*. **15.3 ***Kap1.a* (Hooded lemma 1) is a dominant mutation that causes the appearance of an extra flower of inverse polarity on the lemma. The trifurcate structure consists of a deformed floret at its center with two triangular leaf-like projections called lemma wings. The supernumerary spikelet often contains stamens with fertile pollen grains and occasionally bears a kernel within it. Bowman is to the left in **A** and top in **B**. **15.4 A.** In mutant Subjacent hood 1(*sbk1.a*) the lemma is modified into a sac-like structure also including a short thin awn. **B.** Calcaroides-c mutants (*Cal-c.15*) bear a sac plus pronounced lemma wings. A normal Bowman spikelet to the right. **C.** Spike of *cal-e.23* to the left compared to Bowman

The two dominant Awnless 1 mutations (*Lks1.a* and *Lks1.b*) are likely to have independent origins since they have different SNP markers adjacent to the *lks1* locus on chromosome 2H [23]. Mutants in *lks1* show little or no development of the awn (Fig. 15 (15.1)). Heterozygotes may be awnless [119] or awnletted [120] depending upon the source stock for the *lks1* gene and the genetic background. The *Lks1.a* allele in Engleawnless will not recombine with alleles at the *vrs1* (Six-rowed spike 1) locus [120, 121] because a short paracentric inversion is present in Engleawnless [122]. The complex *vrs1* locus may include awnless and reduced awn length mutants [121]. However, the *Lks1.b* gene in CIho 13,311 does recombine with the *vrs1* locus and is linked to a dominant instead of a recessive allele at the *gth1* (Toothed lemma 1) locus [42]. The awnless trait reduced kernel weight by about 15% and grain yield by about 10% [123–125]. Both backcross-derived lines, BW490 with *Lks1.a* and BW491 with *Lks1.b* [23], are slightly taller than Bowman. The kernels of BW490 are longer and thinner than those of Bowman and weighed 25% less. The kernels of BW491 are similar in size to those of Bowman and weighed 5 to 10% less [42].

In the original stock of the Short crooked awn 1 (*sca1.a*) mutant, awns are reduced to a length of only 2 cm and are curved outward at the tip [126, 127]. Awn tips have a stigma-like appearance in the original stock. In the Bowman backcross-derived line BW769, awns are short (less than 1/4 normal), but awn tips are not strikingly curved or stigma-like (Fig. 15 (15.2)). BW769 plants were slightly shorter than Bowman plants and headed about two days later. Kernels of BW769 were thin (2.82 vs. 3.05 mm in diameter) and light (34 vs. 56 mg). Grain yields of BW769 were 10 to 50% of those for Bowman at various field locations [50].

Hooded lemma 1 (*kap1*) is the gene of the hooded ("Kapuze") trait characterized by an appendage to the lemma, which develops as a trifurcate structure consisting of a deformed spikelet at its center with two triangular leaf-like projections called lemma wings (Fig. 15 (15.3)). The supernumerary floret often contains stamens with fertile pollen grains and occasionally bears a kernel within it [128–130]. The ectopic expression of the dominant *Kap1.a* allele forms the extra spikelet and is associated with the presence of a 305-base pair duplication in intron 4 of the homeobox *Knox3* gene encoding a homeodomain transcription factor [131].

A *Kap1.a*-like phenotype is found in Calcaroides (*cal*) and Subjacent hood 1 (*sbk1*) mutants. These mutants bear a well-organized ectopic structure, the sac, at the tip of the lemma, in a position corresponding to the transition between lemma and awn [79, 97, 132]. The awn is short, thin and threadlike, and the lemma often has pronounced wings (Fig. 15 (15.4)). In contrast to the *Kap1.a* phenotype, the sac does not develop into an epiphyllous flower. Only a few florets of the spike have malformations in the mutants *cal-a.3*, *-a.6*, *-a.7*, and *-a.17*, and these mutants are associated with the formation of pronounced wings [97]. In homozygous conditions, the *sbk1.a* allele is epistatic to *Kap1.a* and *Lks1.a* [101]. The *cal-d* alleles are also associated with leaf curling [97].

### Changes in culm length and composition

**Table:** 

Keywords to find descriptions of mutants in the International Database for Barley Genes and Barley Genetic Stocks (bgs.nordgen.org):
Culm length: brachytic, brh, cud, curly dwarf, dwarf, dwf, extreme dwarf, giant plant, gig, gigas, sdw, semi-brachytic, semidwarf, short culm mutants, sld, slender dwarf, tall culm, tall plant, uzu
Folding of culm: bent culm, bikini, cur, curly, winding dwarf, wnd
Number of nodes: den, densinodosum, many noded dwarf, mnd, nodeless, single internode dwarf, sid
Number of tillers: absent lower laterals, als, corn stalk, cst, cul, gra, granum, int, low number of tillers, many tillers, one tiller, uniculme
Culm strength: brittle culm at maturity, easily lodged plants, fragile stem, fst, stiff straw, weak culm
Growth habit: elongated plants, erect growth habit, fast growing, irregular tillers, lazy, lazy dwarf, lzd, malformed tillers, mft, prostate growth habit, serpentina, slender, sln, slp, srp, upright

Culm morphology of crop plants became of increased interest when fertilizers were introduced in agriculture. Fertilizers stimulate growth and the crop plants produced heavy spikes, which could not be carried by the culm of the earlier cultivars. Therefore, the plants fell over; a process known as lodging. To develop more lodging resistant cultivars, a large number of culm mutants were isolated and evaluated because many dwarfing mutant alleles provided good resistance to lodging. Thus, culm mutants were part of the so-called Green Revolution recognized by the Nobel Prize in Peace given to Norman Borlaug in 1970.

#### Culm-length mutants

**Fig. 16:**
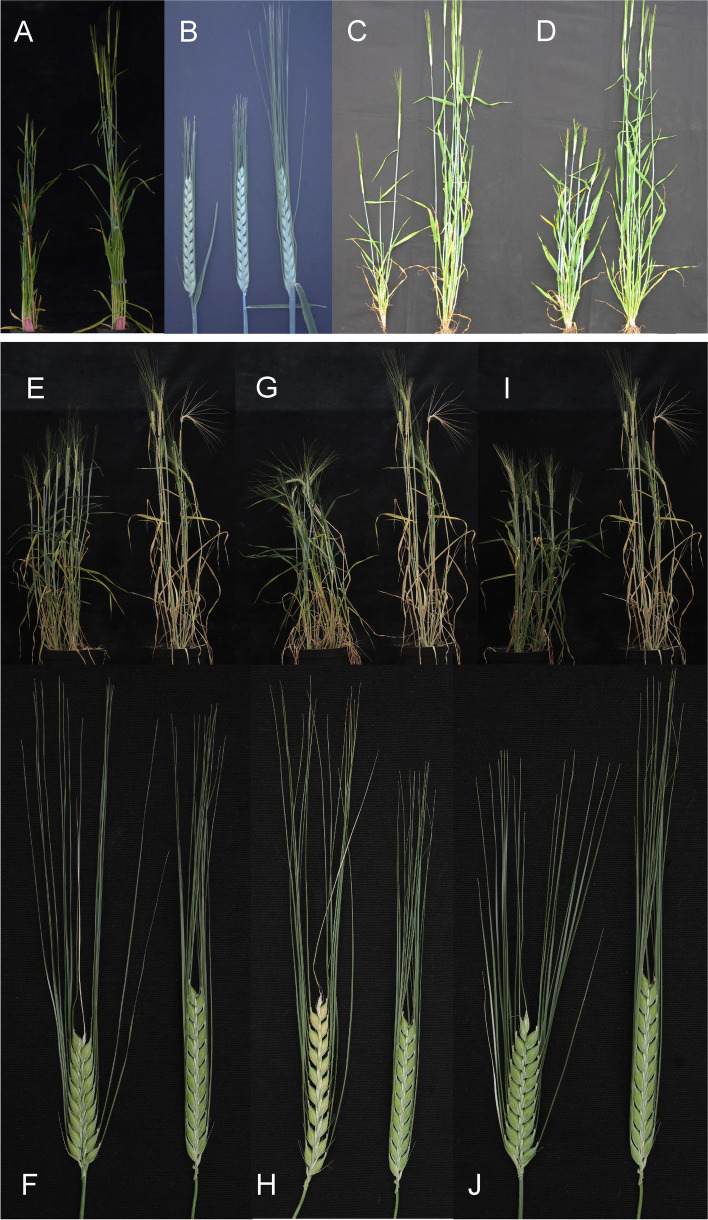
Semi-dwarf mutants. **A.** Mutant Brachytic 1 (*brh1.e*) in the near-isogenic line BW077 to the left compared to Bowman. **B.** Two spikes of BW074 (*brh1.a*) compared to Bowman exemplifies the short awns of many semi-dwarf mutants. **C.** BW515 (*min1.a*, Semi-minute dwarf 1) compared to Bowman. **D.** BW199 (*cud2.b*, Curly dwarf 2) compared to Bowman. **E and F.** *sld2.b*, Slender dwarf 2. **G and H.** *sld3.e.* **I and J.** *sld6.g*. Bowman is shown to the right in **E** to **J**

**Fig. 17:**
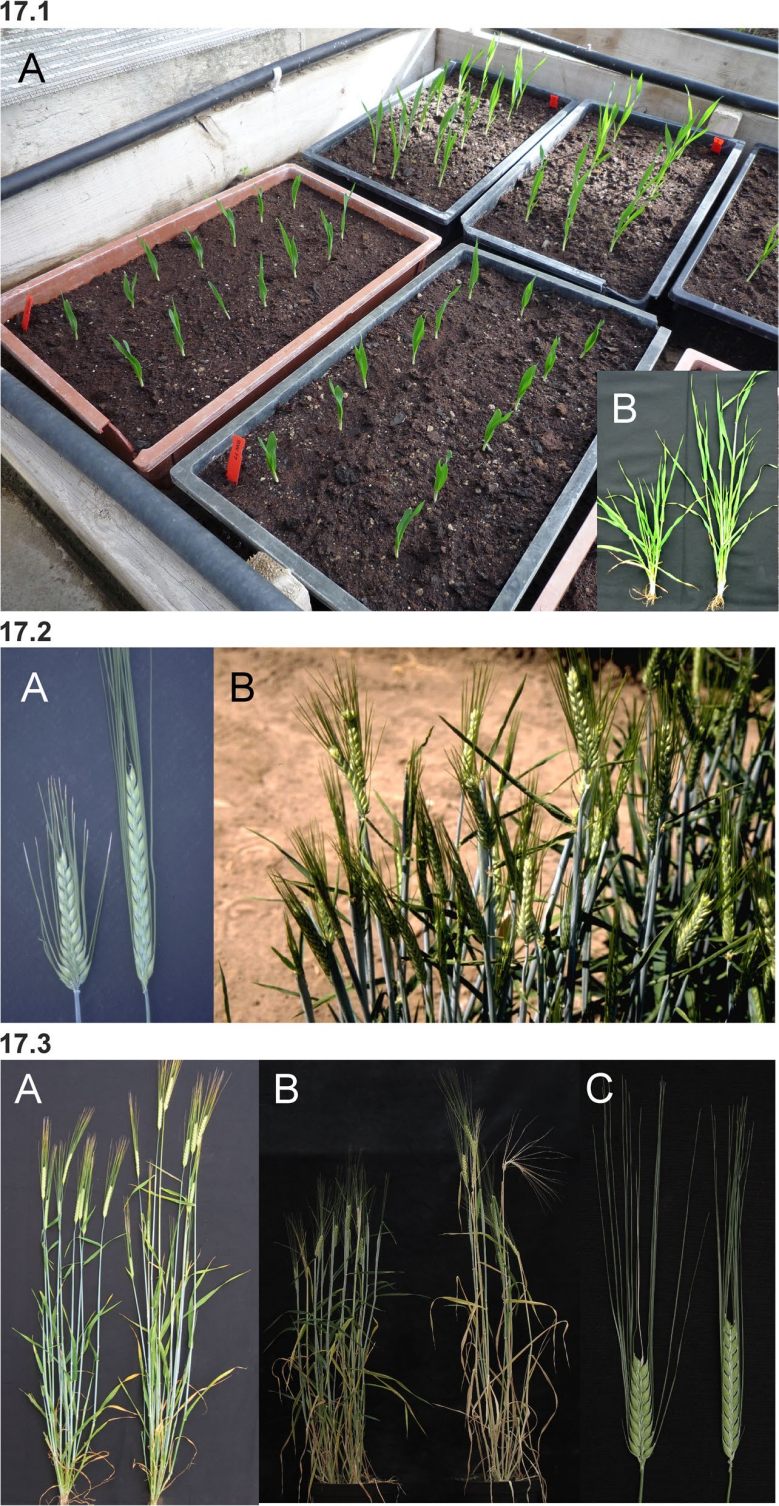
**17.1 A.** Semi-dwarf and dwarf mutants are often easy to identify at the early seedling state as “small and cute” seedlings. The near-isogenic lines BW078 (*brh1.t*, front left) and BW077 (*brh1.e*, front right) compared to more normal seedlings in the back exemplified by BW125 (*cer-w.48*, back left) and BW126 (*cer-x.60*, back right). **B.** Mutant *sdw1.d* (Semidwarf 1, left) can be distinguished from a normal plant also later in the vegetative phase. Most short-culm phenotypes typically appear after transition to the reproductive growth phase. **17.2 A.** Mutant *uzu1.a* (left) compared to cultivar Bowman. The short-awned spike is more compact at the basis. **B.** A row of *uzu1.a*. Opposite spikelets in the tip of the spike can form a crown-like structure and is caused by irregular elongation of the top rachis internodes. **17.3 A.** Mutant *sdw1.d* (Semidwarf 1) is a common allele in short culm barley cultivars due to relatively few pleiotropic effects. The mutation in the near-isogenic line BW828 to the left compared to Bowman. **B and C.** Mutant *sdw2.b* has a slightly stronger phenotype. BW829 (*sdw2.b*) left, Bowman right

Most available culm-length mutants are semi-dwarf plants since these were often found to provide lodging resistance without reducing yield too much. Typically, a semi-dwarf mutant has 50–100% of the culm length of a normal plant. Among the semi-dwarf mutants are Brachytic (*brh*), Semi-brachytic (*uzu*), Semidwarf (*sdw*), Curly dwarf (*cud*), Slender dwarf (*sld*), and Semi-minute dwarf 1 (*min1*) (Fig. 16). Both semi-dwarf and dwarf mutants can often be identified at the seedling stage due to their short seedling leaves with rounded tips (Fig. 17 (17.1)). The *uzu1.a* mutant was one of the first short-culm mutants to be recognized. It was used already a century ago and isolated as a common variant in Japanese landraces [9]. The *uzu1.a* mutant has a semi-dwarf phenotype with 80% of wild-type culm length when grown under standard greenhouse conditions [27]. The elongation of upper-stem internodes is particularly reduced while the stem diameter remains unaltered. Compared to wild-type cultivars, the overall plant architecture is erect, with acute leaf-blade attachment angles. The generally compact spike has short awns and is denser at the base (Fig. 17 (17.2)). The tip of the spike often forms a crown-like structure due to opposite spikelets caused by irregular elongation of rachis internodes [27]. Leaf margins and auricles of *uzu1.a* have a slightly undulating appearance, similar to wild-type cultivars treated with propiconazole, which is a potent inhibitor of brassinosteroid biosynthesis [133]. The *uzu1.a* mutant (BW885) is very sensitive to temperature and the pleiotropic characters described here increase in strength when grown under warm conditions [27]; thus, the mutant is used in few barley production areas other than Northeastern Asia where barley is planted in the fall.

It should be noted that many semi-dwarf mutations have pleiotropic effects and short culms are often found also in other groups of mutants. This can be exemplified by the Erectoides (*ert*) mutants primarily described as dense spike mutants and the Breviaristatum (*ari*) mutants described as short-awn mutants. Similarly, the short-culm *brh* and *uzu* mutants have clearly shortened awns and compact spikes. Further, short-culm mutants often have smaller and globose shaped kernels. In accordance with these observations, it is not surprising that various *brh*, *uzu*, *ert* and *ari* mutants have been shown to be allelic [27]. Thus, the original classification of these mutant groups should not be followed strictly. Instead, they should be regarded as mutants with a generally reduced growth of most plant organs. Many of these mutants have been shown to be deficient in brassinosteroid signaling or metabolism. For example *uzu1.a*, *ari-256* and *ert-ii.79* are all mutated in the gene encoding the barley brassinosteroid receptor [26, 27, 134], and *ari-u.245*, *brh3.g* and *ert-t.437* are deficient in the gene encoding the brassinosteroid biosynthetic enzyme brassinosteroid-6-oxidase [27].

The Semidwarf (*sdw*) mutants appear to have less pleiotropic effects; they are characterized by a reduced culm length but fewer changes in other parts of the plant (Fig. 17 (17.3)). This is probably why the mutant *sdw1.d* is widely spread in many of the spring barley elite malting cultivars presently in use [135]. However, also *sdw1* mutants are associated with negative pleiotropic effects on yield and potentially malting quality [136]. The *sdw1* gene has been shown to encode gibberellin 20-oxidase [137]. Near-isogenic line BW828 (*sdw1.d*) in a cultivar Bowman background was 10–20% shorter than Bowman, heading was delayed 1–3 days, spikes had 1–2 more kernels in some trials, and grain yields were similar [73].

**Fig. 18:**
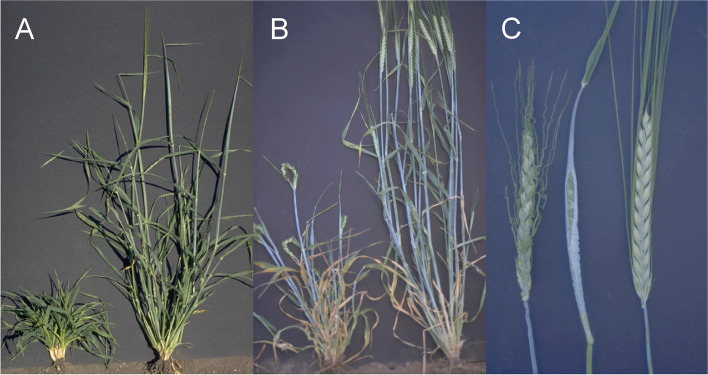
Mutant *cur2.b* (Curly 2). **A and B.** Mutant *cur2.b* in the near-isogenic line BW220 to the left compared to Bowman at different stages of development. At maturity, the mutant is 1/3 to 1/2 of the Bowman height. **C.** The very curly awns of two mutants to the left compared to Bowman

Dwarf mutants have a more severe short-culm phenotype than semi-dwarf mutants. In principle, mutants with 50% or more reduced culm are classified as dwarf mutants. Their strong phenotype makes them less useful in plant breeding. As semi-dwarf mutants, they often exhibit pleiotropic effects. In the Curly mutants *cur1.a*, *cur2.b* and *cur2.g* most parts of the plant are short and twisted, and lemmas and awns are extremely curly (Fig. 18). The rachis is usually bent and tillers and their internodes are curved or wavy [77]. Roots are extensively curled compared to the straight or slightly coiled roots of normal plants [138]. Seed set is poor in most environments.

Also, extreme dwarf mutants have been found. The mutant Enhancer of minute 2 (*min2*) is less than five centimeters tall. It is expressed only in plants homozygous for the *min1.a* allele [139]. The leaf blade and sheath are very short and thick and have a whitish dark green color. Roots are thick and short with C-tumor-like swelling at their tips. No spikes are formed; hence, the stock must be maintained as a heterozygote at the *min1* locus. Among the kernels produced by heterozygous plants, those that will give rise to minute plants have a markedly shrunken endosperm [140]. Plants of the Bowman backcross-derived line for *min1.a*, BW515, are about half as tall as Bowman.

**Fig. 19:**
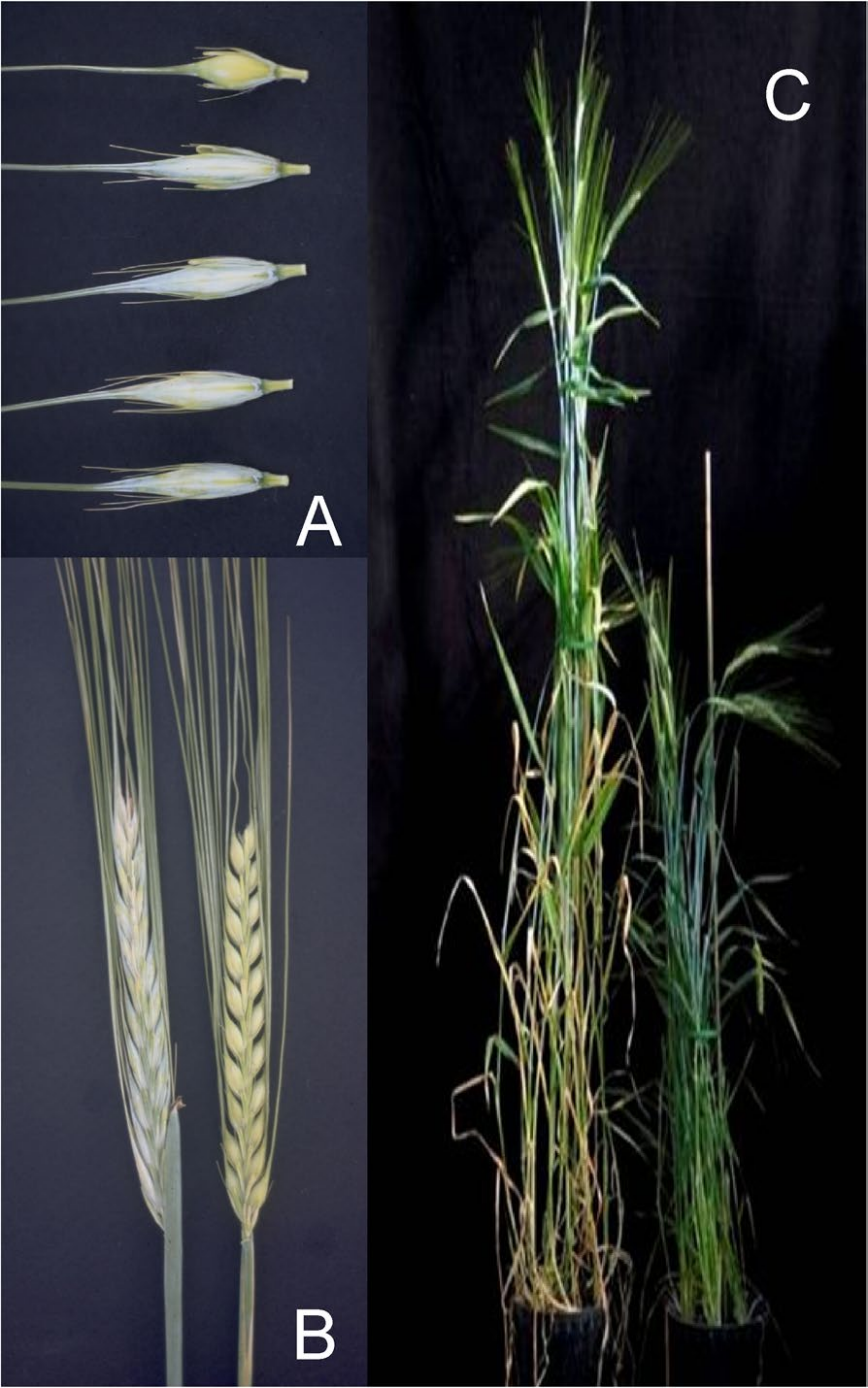
**A.** Four spikelets of BW381 (*gig1.a*, Gigas 1) compared to one spikelet of Bowman (top). The *gig1.a* spikelets have a pronounced wax coating. **B.** Spike of BW381 (left) compared to Bowman (right). **C.** BW382 (*gig2.c*) is almost twice as tall as Bowman

A few tall-culm mutants are available. Most parts of Gigas 1 (*gig1.a*) plants are slightly larger than wild-type cultivars, i.e. culm, leaves, spikes and glumes. In contrast awns are slightly shorter. Spikes are lax with a longer distance between spikelets. Culms, spikes and leaf sheaths have a heavy wax coating (Fig. 19). Anther development in *gig1.a* plants is good, but the stigma has few hairs and seed set is poor. In the Bowman backcross-derived line BW381 *gig1.a*, plant height is normal. The mutant Gigas 2 (*gig2.c*) is considerably larger in most parts and nearly twice as tall compared to the wild-type cultivar (Fig. 19). The Bowman backcross-derived line BW382 *gig2.c* has 2 to 9 more kernels per spike, but kernels are shorter, narrower and lighter resulting in grain yields 25–50% lower than those for Bowman. The transition from vegetative to reproductive growth is much delayed and compared to Bowman heading is often 2–4 weeks later. When planted late in North Dakota, *gig2.c* plants may remain in vegetative phase until the end of the growing season. Under field conditions, plants lodge easily.

#### Folding of culm

**Fig. 20:**
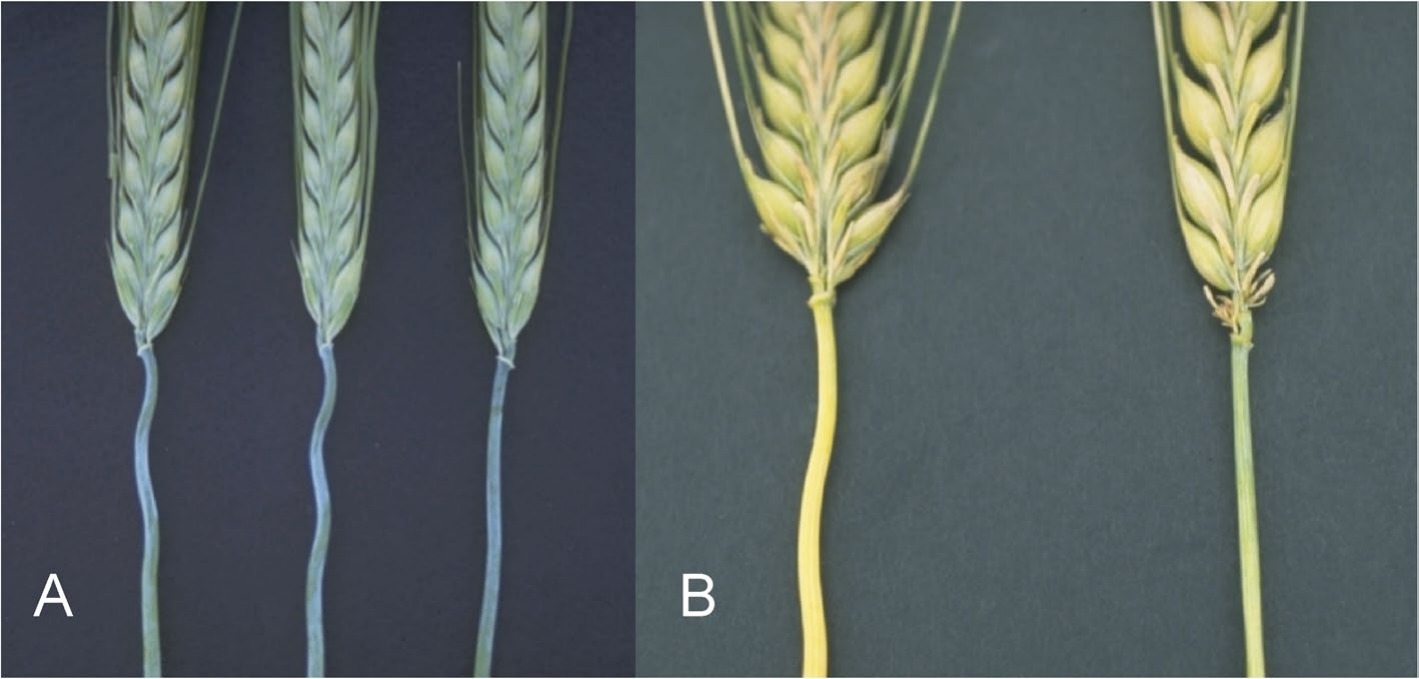
**A.** Two spikes of the near-isogenic line BW906 carrying the *wnd1.a* (Winding dwarf 1) allele to the left compared to Bowman. The mutant is characterized by a coiled upper portion of the first internode. **B.** The original *wnd1.a* mutant to the left carries also the *dense spike 1* (*dsp1.a*) allele which results in a compact spike

The culms of the barley plant are slightly twisted or coiled. In the Curly mutants, previously named Bikini, extreme twisting occurs. Their stem internodes are strongly curved and leaf blades are short and severely twisted [101, 126]. Further, the awn, lemma and palea are extremely curly, and the rachis is slightly twisted in most spikes (Fig. 18). A milder phenotype is found in Winding dwarf 1 (*wnd1.a*). As a near-isogenic line in the Bowman genetic background, *wnd1.a* has a coiled upper portion of the first internode (Fig. 20). The original stock for the *wnd1.a* gene also contains the dense spike (*dsp1.a*) gene and the plant shows a semi-dwarf phenotype with a pronounced coiling or winding of the upper part of the peduncle.

#### Number of nodes

**Fig. 21:**
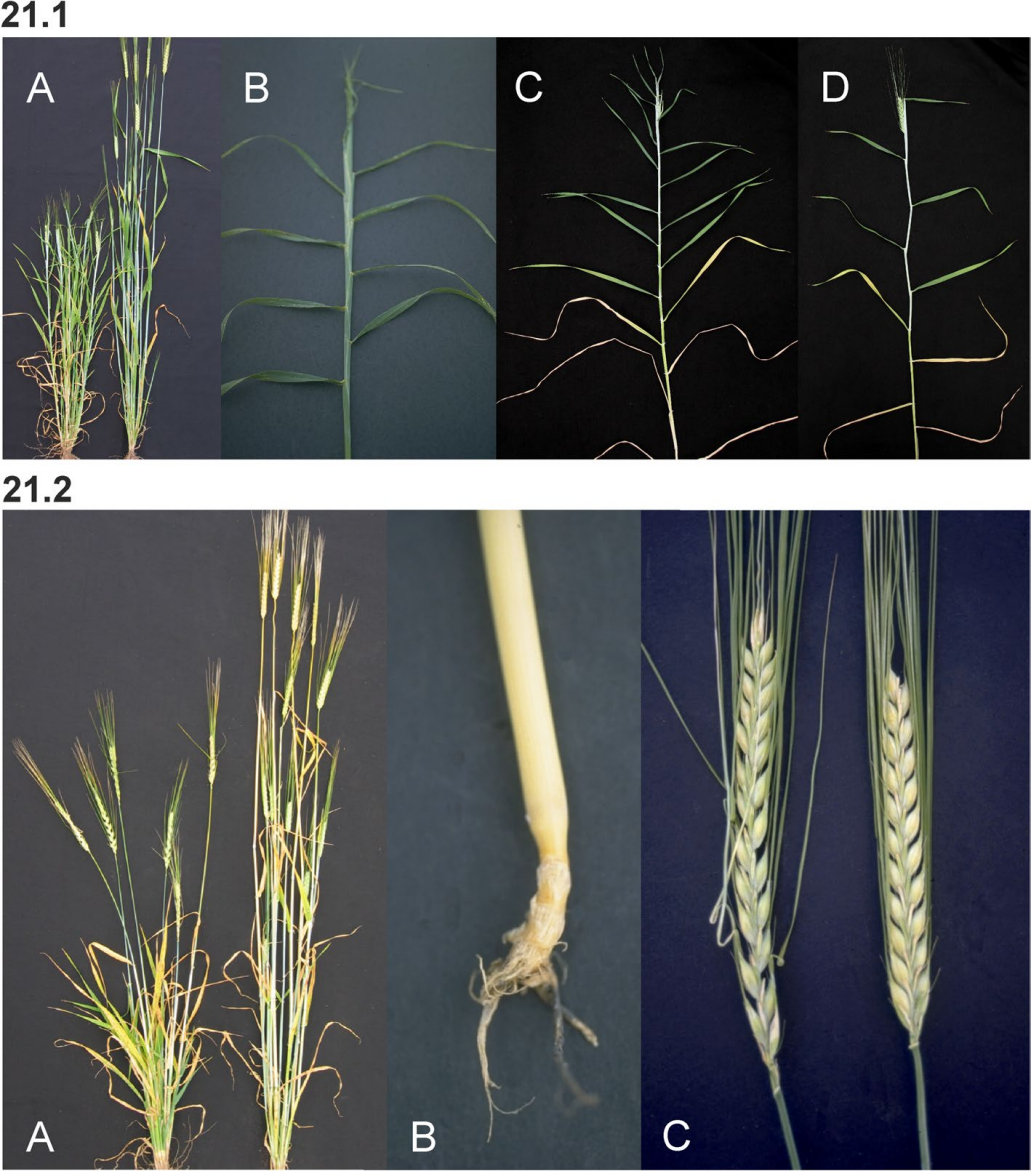
**21.1 A.** Mutant *mnd1.a* (Many-noded dwarf 1) to the left compared to cultivar Bowman. **B, C and D.** A single culm of *mnd1.a*, *mnd5.g* and *mnd6.6*, respectively. **21.2 A.** Mutant *sid1.a* (Single internode dwarf 1) to the left compared to cultivar Bowman. **B.** The lower part of *sid1.b* with only one single elongated internode (the peduncle) and concentration of nodes at the base of the plant. **C.** A spike of *sid1.b* to the left compared to Bowman

The culm of grasses is a series of nodes and internodes, called phytomers [103]. The node is located between two consecutive internodes and can be seen as swollen regions (“knees”) on the culm. The nodes contain intercalary meristematic cells, which for example allow grasses to successfully regrow in response to damage by grazing herbivores or change direction of growth when tilted over. In cultivated barley, the culm typically has 5–6 nodes in which internode elongation occurs. The distance between nodes is shorter at the base of the plant, and it can therefore be problematic to determine the exact number of nodes. For convenience, internodes are numbered from the top to the bottom, i.e. the first internode is the peduncle which connects the spike with the culm. Mutants with fewer or more culm nodes or phytomers are available. The group of Many-noded dwarf (*mnd*) mutants can have up to 20 nodes. The plants are about half of normal height with numerous tillers branching from lateral meristems [141, 142]. From a distance, they give a bushy appearance due to an increased number of short leaf blades associated with the increased number of nodes (Fig. 21 (21.1)). Spikes often develop poorly and heading is late. Milder phenotypes are known such as *mnd3.d* and *mnd4.e*, with only 1–2 and 7–8 extra nodes, respectively [42].

Mutants with fewer elongated internodes are found among the Single internode dwarf (*sid*) mutants (Fig. 21 (21.2)). Mutants in the *sid1* locus have all stem nodes crowded together close to the secondary root system and the stem is formed from a single elongated terminal internode [143, 144]. The mature plant has several culms, each having only one single elongated internode [145]. Plants are relatively weak and partially sterile, and have very lax spikes [42]. The expression of mutant traits is less extreme in *sid1.b* plants, where tillers often have two elongated internodes. Fertility is also better in *sid1.b* and the spike is not as lax. The single elongated terminal internode was approximately 80% of plant height in the Bowman backcross derived lines for *sid1.a* (BW849) and *sid1.b* (BW850). Compared to Bowman, kernels of *sid1* mutants were thinner and 10–15% lighter [50].

#### Number of tillers

**Fig. 22:**
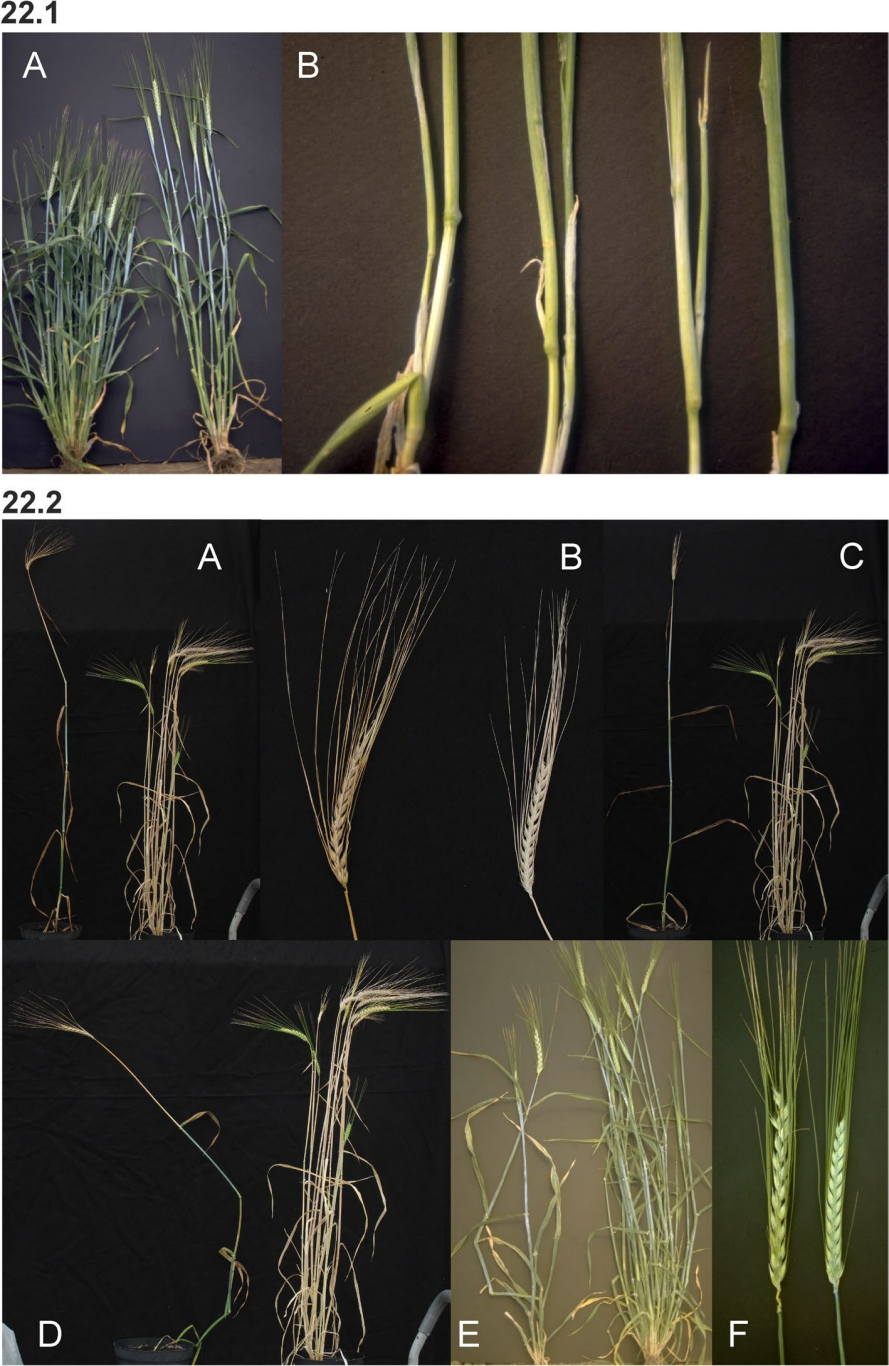
**22.1 **Mutants in the *gra-a *(Granum-a) locus have an increased number of tillers, which are thin with narrow leaves and short internodes. **A.** Mutant *gra-a.1* to the left compared to Bowman. **B.** The tiller formation at the base of three *gra-a.2* mutants compared to Bowman (right). **22.2** Barley Uniculme (*cul*) mutants. Cultivar Bowman to the right in each photo. **A and B.** Near-isogenic line BW206 (*cul2.b* + *rob1.a* (Orange lemma 1). **C.** BW207 (*cul3.c*). **D.** BW211 (*cul4.3*). **E and F.** BW212 (*cul4.5*)

The number of tillers can vary in any barley cultivar or mutant line depending on planting density and timing of planting. A solitary plant in the field or in a large pot in the greenhouse generally develops more tillers than a plant standing in a group of other plants or in a small pot. Similarly, a spring barley cultivar planted early in the season will develop more tillers than the same cultivar sown later. Still, there could also be genetic reasons for the number of tillers. In Granum-a (*gra-a*) mutants the number of tillers can be more than double that of an ordinary cultivar (Fig. 22 (22.1)). The tillers are thin with short internodes and narrow leaf blades. Culms are short in these mutants, which classifies them also as semi-dwarfs. The reduced size also includes the spike, which has short awns, a reduced number of kernels as well as lighter kernels [50].

In Uniculme (*cul*) mutants, the number of culms per plant is reduced. A severe phenotype is seen in the *cul2* and *cul3* mutants, which have a single elongated culm (Fig. 22 (22.2)). The culm is usually straight and has a much greater diameter than normal, and plants usually head earlier than normal [146]. Kernels of the backcross-derived line for *cul2.b*, BW205, were longer and wider than Bowman kernels and on average weighed slightly more [55]. The *cul2.b* plants initiate vegetative axillary meristems, but tillers fail to develop [147]. Irregular placement of some spikelets and partial female sterility of lateral spikelets occurs in the original stock [148] and in the Bowman backcross-derived line [147]. Yield of *cul* plants is not restored when grown under high plant densities [149]. Double mutant combinations with *als1.a*, *lnt1.a*, *cul4.5*, *int-b.3* and *uzu1.a* resulted in a *cul* vegetative phenotype [147]. Stress response genes are upregulated in *cul2.b* mutants [150].

Mutants in the *cul4* locus produce 1 to 4 tillers that are twisted and have slightly bowed culm internodes. All secondary tillers are shorter than the primary tiller and have a curly appearance. Often secondary tillers are trapped at the base of the primary tiller [151]. Compared to Bowman, *cul4* plants of the Bowman backcross-derived lines for *cul4.3* (BW211) and *cul4.5* (BW212) had peduncles that were 50% longer. Rachis internodes were slightly elongated, and kernels were slightly longer. Plant height varied from 2/3 normal to slightly taller than Bowman. BW212 exhibited more variation in height over environments [151]. Under greenhouse conditions, BW212 developed only two axillary tillers, and it was uniculm when combined with the *cul2.b* allele [147]. All *cul4* mutants have a liguleless phenotype [152], and the ability to produce more than one axillary bud [153]. Morphological, histological and in situ RNA expression analyses indicated that the dominant allele at the *cul4* locus (homolog of Arabidopsis genes *BLADE-ON-PETIOLE1* (*BOP1*) and *BOP2*) acts at axil and leaf boundary regions to control axillary bud differentiation, as well as development of the ligule [153]. The barley *cul4* gene and its paralog *lax-a* are primarily involved in regulating tiller number and spike morphology, respectively. Analysis of natural alleles at the *cul4* locus identified 31 haplotype variants [70].

**Fig. 23:**
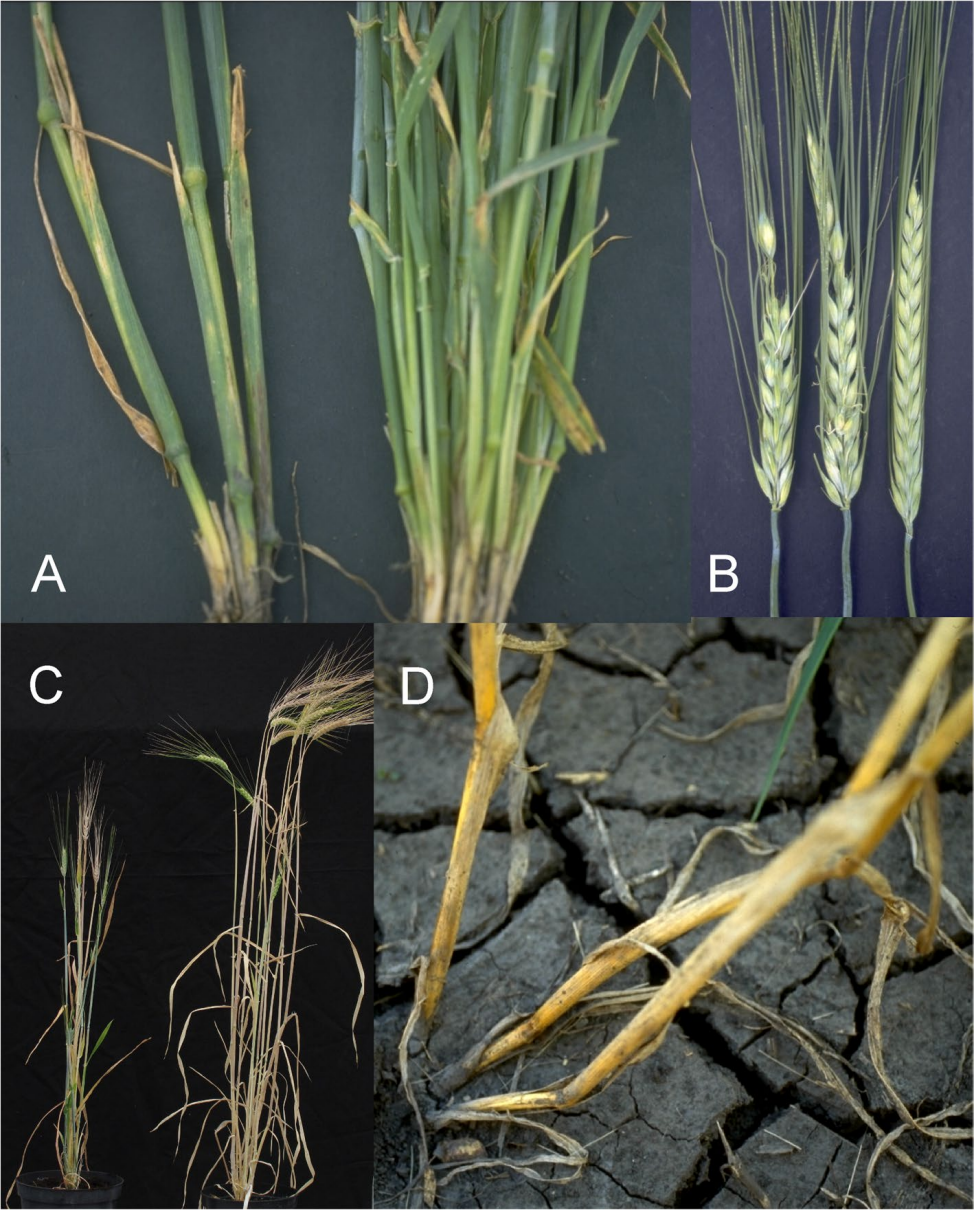
Barley mutants with a reduced number of tillers. **A.** Mutant *lnt1.a* (Low number of tillers 1) to the left, Bowman to the right. **B.** Two spikes of *lnt1.a* compared to Bowman. The *lnt1.a* mutation causes asymmetry and irregularity. **C.** Corn stalk 1 (*cst1.a*) compared to Bowman (right). **D.** Mutant Absent lower laterals 1 (*als1.a*) forms only a few tillers which are coarse and stiff

A reduced number of tillers are also seen in Corn stalk 1 (*cst1*), Low number of tillers 1 (*lnt1*), and Absent lower laterals 1 (*als1*) mutants (Fig. 23). The *cst1.a* mutant produces semi-dwarf uniculm plants with thick culms in six-rowed barley, and seed set is moderate to very low. When *cst1.a* allele was backcrossed into the two-rowed barley Bowman, increased tillering and improved seed set were observed [42]. Plants of the Bowman backcross-derived line BW197 were about 3/4 normal height and spikes were compact. Awns, peduncles, and leaf blades of BW197 were about 3/4 the length of those of Bowman. The kernels of BW197 were slightly shorter than those of Bowman and the seed weight was lower. Grain yields of BW197 were very low [42].

The few tillers produced in the mutant *als1.a* are coarse and stiff (Fig. 23). In six-rowed cultivars, the number of tillers is typically 1–2, while 3–4 are normally found in two-rowed lines [71, 76]. The spike in *als1.a* is malformed due to irregular placement of central and lateral spikelets (see Chapter 2.2).

Two alleles of *lnt1* (*lnt1.a* and *int-l.81*) are known [47, 154]. The tiller number is reduced to 2–4 per plant in mutants at the *lnt1* locus. These tillers are formed soon after seedling emergence; hence, no late-emerging tillers are observed [155]. Culms are thick and stiff and leaves are dark green [154]. Spike malformations occur in most environments. Spikes may have irregular rachis internode lengths and are relatively short (Fig. 23). The lower portion of the spike appears more compact than the upper portion [155]. Grain yields of the backcross-derived lines for *lnt1.a* (BW494) and *int-l.81* (BW428) were about 10% of those of Bowman. Kernels were longer, wider and heavier by up to 20% [51]. Double mutant plants with the *lnt1.a* and *int-b.3* genes produced uniculm plants [155]. The *lnt1.a* gene showed an epistatic interaction with high tillering mutants *gra-a.1*, *int-m.85*, *mnd1.a* and *mnd6.6*, producing double mutant plants with 2 to 3 tillers [155].

#### Culm strength

**Fig. 24:**
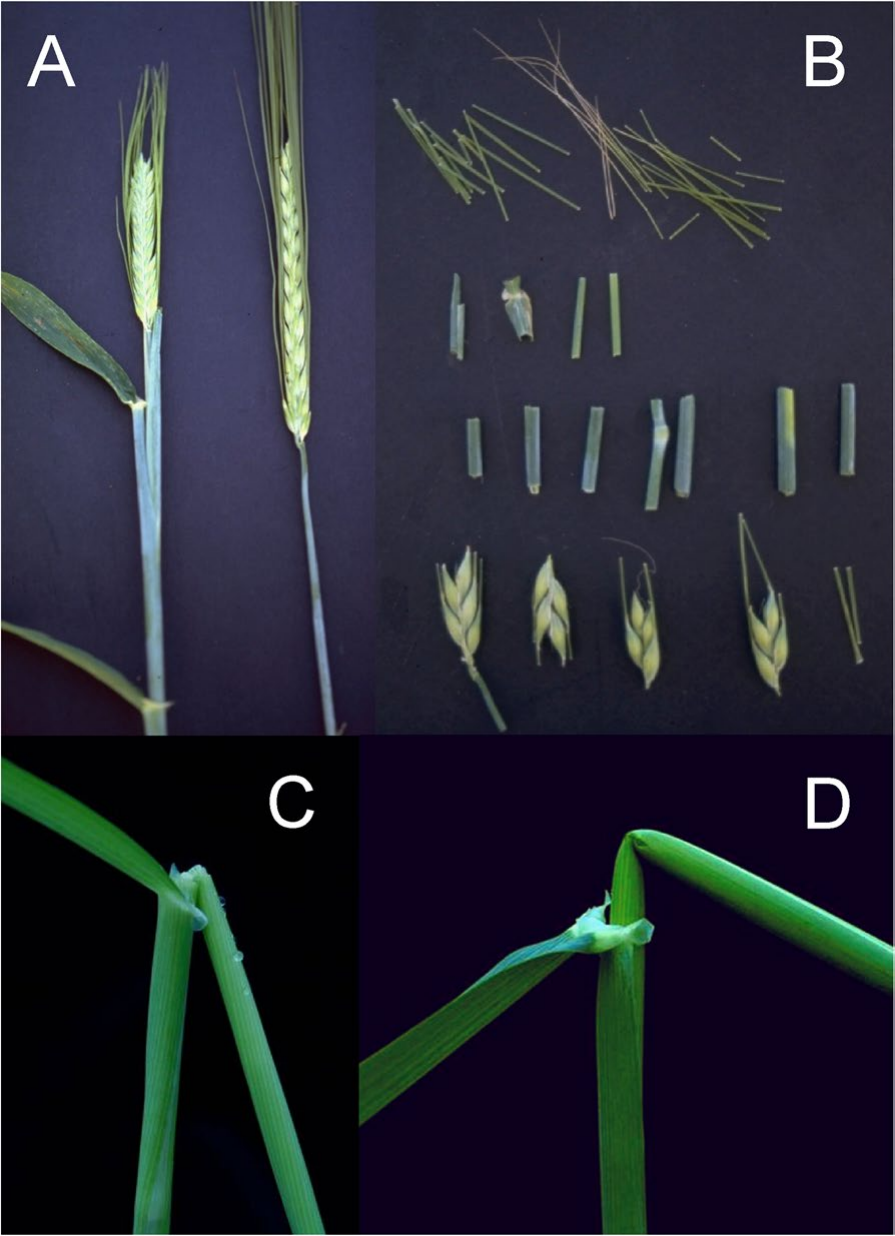
Three loci are associated with fragility. In these mutants leaves and stems are easily broken when physically bent. **A.** Mutant Fragile stem 3 (*fst3.c*, left) demonstrates the dwarfish of the *fst* mutants compared to Bowman. **B.** Small broken pieces of *fst2.b*. **C.** Mutant *fst1.a* obtain an open wound when physical bent, which does not occur in Bowman **D**

Brittle stems and leaves are characteristic of Fragile stem (*fst*) mutants (Fig. 24). The *fst2* and *fst3* mutants have a short-culm phenotype that can be observed when grown in the greenhouse but under field conditions they are severely damaged by the wind. The maximum flexural load (Newtons) required to bend the midpoint of each internode was 2–3 times lower than the load causing bending in their parents [156–158]. The *fst2.b* mutant has a reduced level of crystalline cellulose in the culm compared to their parental lines [158, 159], which fits with a reduced level of mRNA for the *HvCesA4* cellulose synthase gene [156].

#### Growth habit

**Fig. 25:**
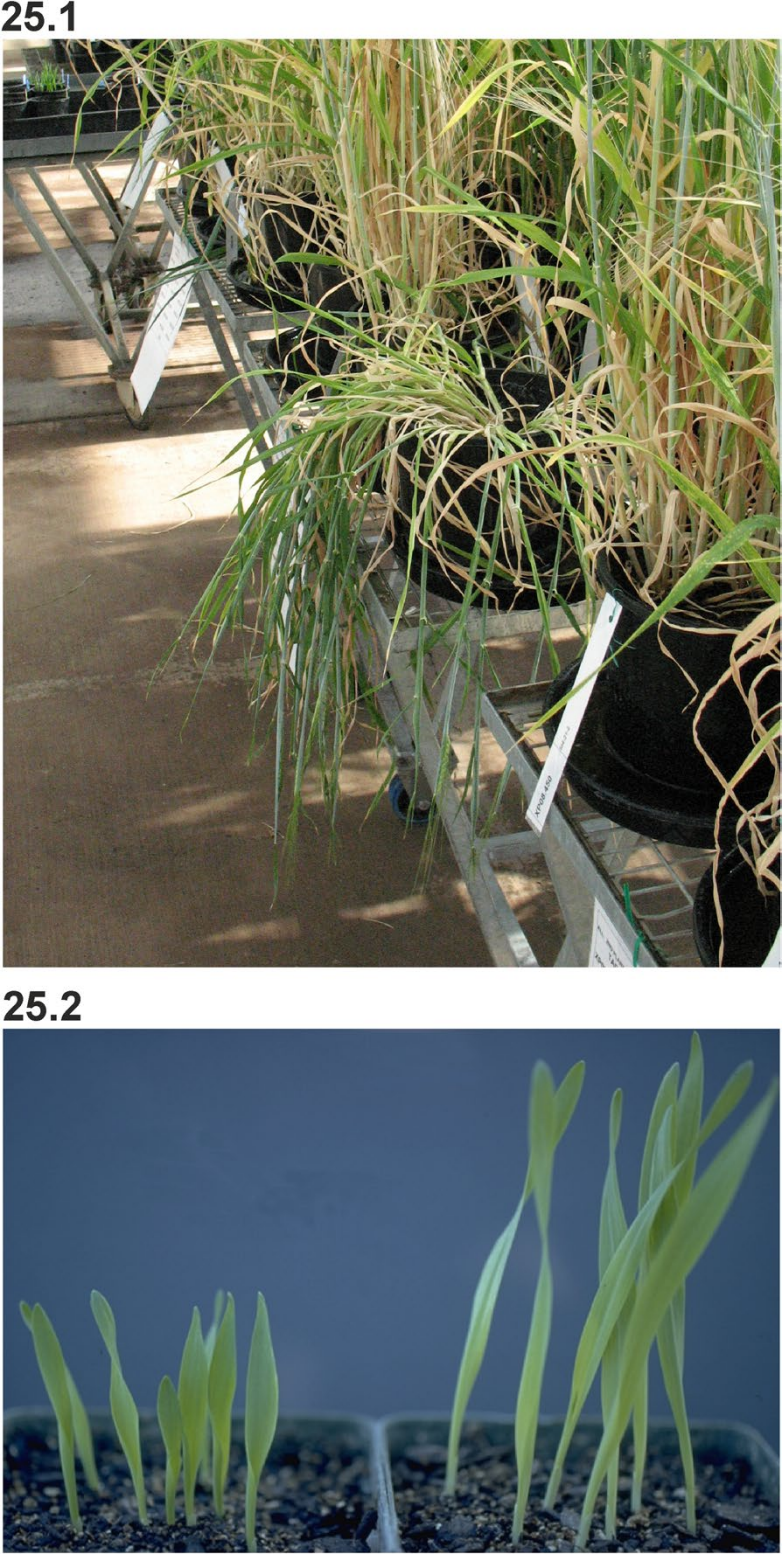
**25.1 **The near-isogenic line BW875 expressing the *srp1 *(Serpentina 1) gene has lost its ability to grow upward. **25.2 **Seedlings of *lzd1.a* (Lazy dwarf 1) and Bowman (right)

This group consists of very divergent mutants, often described by their way of growing. The Serpentina 1 (*srp1*) mutant has lost its ability to grow upright and instead grow more or less parallel to the ground (Fig. 25 (25.1)). When grown in a pot, they fail to respond to gravity and grow downward. Probably they have lost their ability to sense gravity [160, 161]. The Lazy dwarf 1 (*lzd1.a*) mutant has a normal gravitropism response, but tillers arise at rather wide angles before becoming partially erect. Seedlings have a dwarf phenotype (Fig. 25 (25.2)) and are very responsive to gibberellic acid. Plants are 3/4 normal height and maturity is delayed [162, 163]. The *lzd2.b* mutant is more reduced in size but has a similar growth habit.

### Changes in leaf blades

**Table:** 

Keywords to find descriptions of mutants in the International Database for Barley Genes and Barley Genetic Stocks (bgs.nordgen.org):
Size of leaf: angustifolium, blf, brachytic leaf blade, broad leaf blade, fol, latifolium, leafless, lfl, narrow leaf, narrow leaf blade, narrow leafed dwarf, nld, nlf, small leaf blade
Changed ligule and auricule: auricleless, eli, eligulum, exauriculum, exligulum, lig, liguleless, no ligule, small ligule
Leaf folding: clh, coiled leaf blade, curled leaf dwarf, curly leaf blade, folded leaf, olp, onion-like leaf blades, reverse folding, revoluted leaf blade, rolled leaf blade, rvl, scirpoides, scirpoides leaf, sci, scl, tlf, tube leaf
Hairy leaf sheath: hairy leaf sheath, hsh, pub, pubescent leaf blade, pubescent lower leaf sheaths

The leaf is the main photosynthetic organ of plants. The light-capture efficiency undoubtedly differs depending on the leaf morphology and is essential for the survival of plant species [164]. The leaf is composed of the sheath and blade with the auricles and ligules between these two structures. Variation in architecture and positioning of leaves affects crop yield, which in turn is an important breeding trait [165–167].

#### Size of leaf

Different factors affecting leaf size and shape in grasses have been found [168–173].

**Fig. 26:**
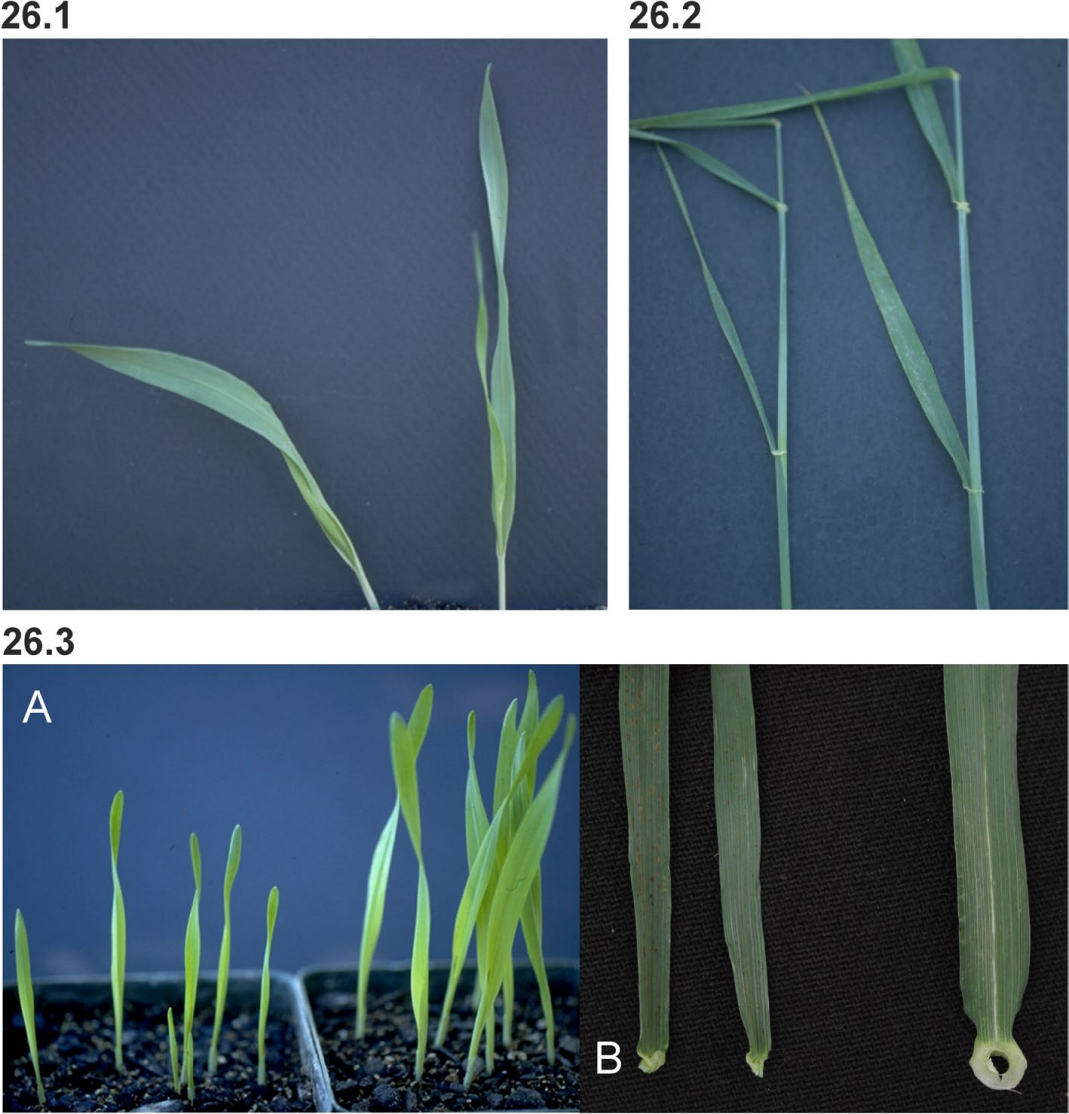
**26.1** Broad leaf 1 (*blf1.a*) leaf blade to the left compared with Bowman. **26.2** Narrow leaf blades in Angustifolium-a (*fol-a.1*) to the left compared to Bowman. **26.3 A.** Narrow leafed dwarf 1 (*nld1.a*) seedlings to the left compared with Bowman. **B.** Two leaves of Narrow leafed dwarf 2 (*nld2.b*) compared with Bowman

Barley mutants characterized by changes in the size of the leaf can be divided into two classes: broad leafed and narrow leafed mutants. Broad leaf 1 (*blf1*) mutants are characterized by wider leaf blades. The *blf1.a* mutant was induced by X-rays in the cultivar Bonus [77]. Plants have leaf blades that are approximately two times wider. It was noted that the width of the lemma, palea, and kernel were increased as well in the mutant [174]. Plants are lighter green and leaves are notably crinkled, specifically at the margins. Leaf blades of the Bowman backcross-derived line BW058 (*blf1.a*) are two times wider than those of Bowman [165] (Fig. 26 (26.1)). Allelic mutants have been isolated from screening of a TILLING population made in the cultivar Barke and by sequencing of the identified *blf1* gene in mutants with broad leaves [165]. It was shown that increase in blade width is due to increase in cell number across the leaf blade, but not due to increase of cell size [165]. The *blf1* gene controls barley leaf size by reducing cell proliferation along the leaf-width. The Maculosus-3 (*blf3*) mutant has a broad leaf, which is retained in the Bowman backcrossed derived line BW503.

The second class with changes in leaf size is characterized by narrow leaves and includes mutants Angustifolium-a *(fol-a),* Angustifolium-b *(fol-b),* Narrow leafed dwarf 1 *(nld1)*, Narrow leafed dwarf 2 *(nld2)*, and Broad leaf 2 (*blf2*). Plants with mutations in *fol-a* are characterized by narrow and dark green leaves, and the majority of organs are decreased in size [175] (Fig. 26 (26.2)). In a Bowman genetic background, *fol-a.1* (BW370) is a little shorter than Bowman and leaf blades are relatively short. Rachis internodes of BW370 were shorter (approximately 4.4 versus 4.9 mm), the size and weight of the kernels were decreased, but grain yields were somewhat increased. BW370 plants lodged easier than Bowman [55]. Homozygous *fol-b* plants are very weak and often cannot survive beyond the 3- to 4-leaf stage. On the contrary, heterozygous plants are not lethal and have a good vigor. Mutants in *fol-b* need to be kept in heterozygous stocks. Heterozygous *fol-b* plants have narrow but not thread-like leaves as homozygous mutants do [176]. *Fol-b.2* was backcrossed to Bowman and the resulting BW371 line showed reduced height, delay in heading for a few days, and low grain yield.

Mutations in *nld1* and *nld2* show pleiotropic effects. The mutants have not only a narrow leave phenotype but are also semi-dwarfs with a generally short phenotype (Fig. 26 (26.3)). The gene responsible for the *nld1* phenotype is an ortholog of the maize *NARROW SHEATH* gene. The gene plays a central role in the expansion of organ width and in the development of marginal tissues in lateral organs in barley [164]. The *nld2.b* plants are characterized by narrow and dark green leaves. Leaf blades of *nld1.a*, *nld1.d* and *nld2.b* have well-developed midribs and are erect. Culm internodes of these mutants are short, and the upper internodes are twisted. The ligules of *nld2.b* plants are normal, but auricles degrade to tiny projections. Kernels of the BW636 (*nld2.b*) line were thinner and lighter than those of Bowman. Vigor of BW636 (*nld2.b*) is stronger than those of BW635 (*nld1.a*) when grown in New Zealand and in North Dakota greenhouses, but BW635 (*nld1.a*) had more vigor in the field trial at Dundee, Scotland [55].

#### Changed ligule and auricle

**Fig. 27:**
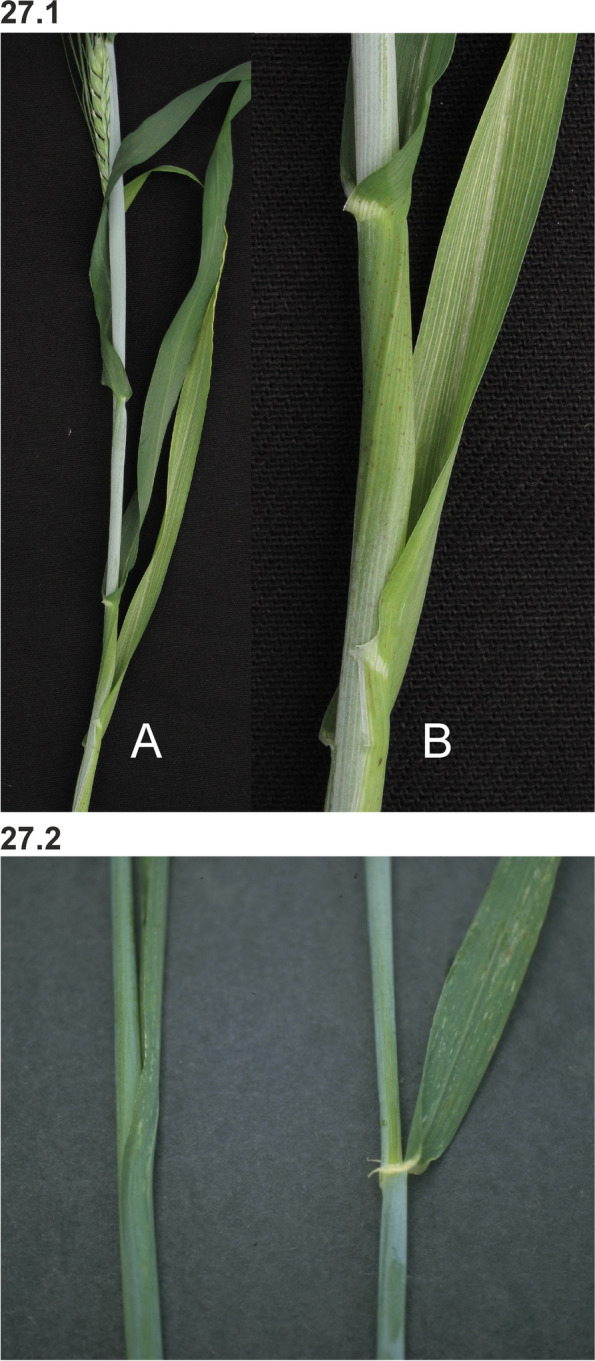
**27.1 A.** Single culm of Eligulum-a (*eli-a.3*) with typical eligulum formation. **B.** A close-up of the *eli-a.3* leaf sheath. **27.2 **Liguleless 1 (*lig1.my*) tillers are missing both ligule and auricle of all leaf blades. Bowman is to the right

The ligule is a protecting outgrowth located between leaf blade and leaf sheath. Ligules hinder water getting inside the leaf sheath and in this way protect the internodes from pathogen infection and rotting. Auricles are small ear-like projections, which are located at the lower part of the leaf where the leaf blade and sheath join. Several mutants have been isolated that have defects in ligule and auricle formation. Those include Eligulum and Liguleless mutants. Eligulum-a (*eli-a*) mutants (Fig. 27 (27.1)) do not have a ligule. The auricles are rudimentary and asymmetrically displaced. The peduncle is short, and the emerging spike is often trapped in the leaf sheath. Spikes have a compact arrangement of spikelets, and the culm is very fragile and breaks easily between the upper and lower halves of the nodes. There are more than ten *eli-a* allelic mutants, which were generated in different backgrounds. Liguleless 1 (*lig1*) mutants are missing both ligule and auricle of all leaf blades. Liguleless 1 mutants can be identified at all growth stages since they have very erect leaf blades (Fig. 27 (27.2)). Two allelic Liguleless 1 mutants, *lig.1.2* and *lig1.my*, were backcrossed to Bowman generating lines BW 482 and BW483, respectively [23].

#### Leaf folding

**Fig. 28:**
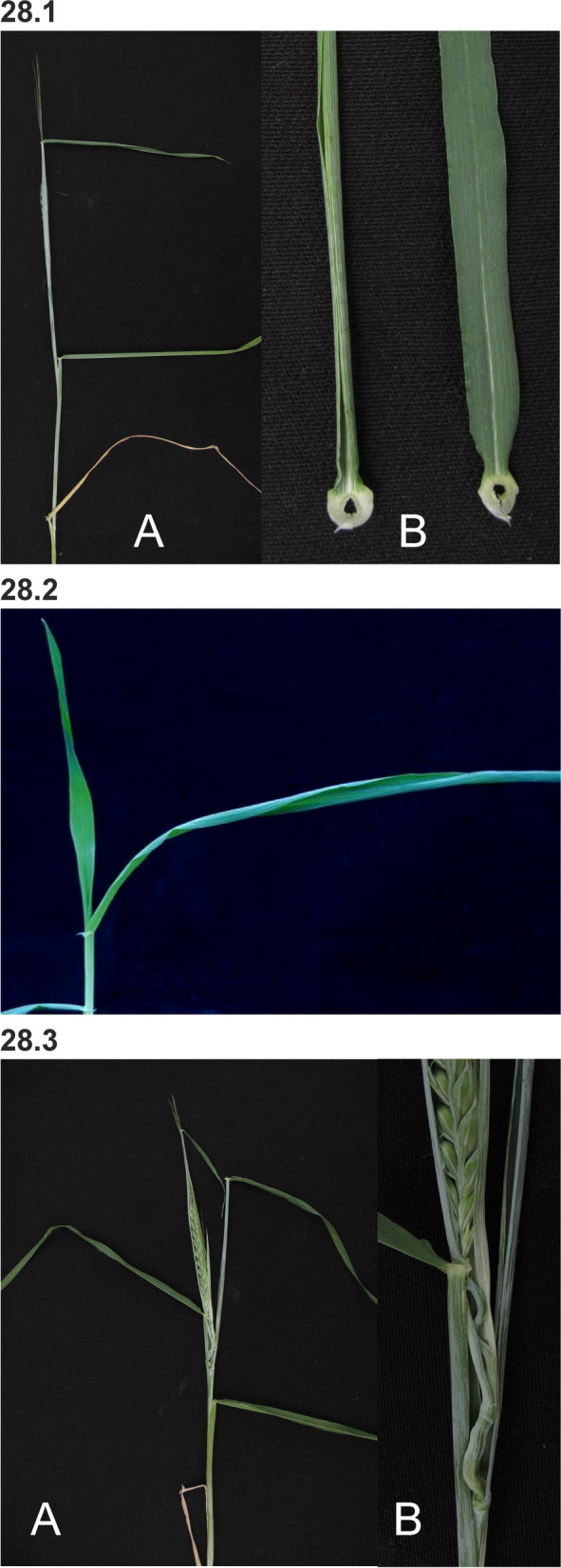
**28.1** The inward U-shaped folding of leaf blades of Scirpoides-b (*sci-b.4*). Bowman is to the right in **B**. **28.2 **Revoluted leaf blade 1 (*rvl1.a*) rolled into a tube through a counterclockwise spiral. **28.3 A.** Scirpoides leaf-b (*scl-b.5*) single culm with spike. **B.** Close-up of the base of a spike with curled upper basal rachis internode and typical rolled leaf blades

The group of leaf folding mutants are dominated by mutants having narrow leaf blades with an inward fold that results in U-shaped leaf blades (Fig. 28 (28.1)). The mutants include Scirpoides-a (*sci-a*), Scirpoides-b (*sci-b*), Scirpoides leaf-a (*scl-a*), Scirpoides leaf-b (*scl-b*), and Curled leaf dwarf 1 (*clh1*). A single mutant, *rvl1.a* (Revoluted leaf 1), has a phenotype where the tips of young leaf blades can roll into a tube through a counterclockwise spiral (Fig. 28 (28.2)). Leaf blades are slightly folded until maturity. Compared to Bowman, plant height of Bowman backcross-derived line BW778 (*scl-a.6*) were slightly reduced and grain yields were about 20% lower [50].

Scirpoides leaf-b is represented by one mutant, *scl-b.5* (Fig. 28 (28.3)) [92]. When grown in the greenhouse, *scl-b.5* plants had very narrow leaf blades that showed more inward folding than *scl-a* mutants. Also, the awns were often trapped by the collar of the flag leaf during spike emergence [151].

Plants expressing a *sci-a* allele appear to have very narrow, inward folded leaf blades. The tip of an emerging leaf blade is sometimes trapped inside the previous one. Folding of the leaf blade persists until maturity. Premature yellowing of leaf blade tips may occur shortly after heading. Spikes are 1/2 to 2/3 normal length and plant height is reduced slightly [50]. Compared to Bowman, plants of the Bowman backcross-derived lines for *sci-a.1*, BW772, and *sci-a.3*, BW773, were slightly shorter than Bowman, with slightly shorter rachis internodes, and had 1 to 3 fewer kernels per spike. Kernel weight for BW772 and BW773 were slightly lower in some trials. Grain yields ranged from 40 to 70% of those for Bowman. However, BW772 often yielded slightly less than BW773 [50].

Plants of mutant *sci-b.4* have narrow leaves and lower leaf blades are folded inward (Fig. 28 (28.1)) [151]. In the Bowman backcross-derived line for *sci-b.4*, BW771, rachis internodes were slightly elongated. BW771 plants were 2/3 to almost normal height, kernels were slightly smaller, and lighter, and seed yields were about 3/4 of normal [151].

Leaf blades of *clh1.a* (Curled leaf dwarf 1) are erect, narrow, and folded inward and appear thicker than normal. Plants show reduced vigor, fertility and height (3/4 of normal) when grown in the greenhouse. They show further reduction in vigor and fertility when grown in the field. Spikes emerge poorly from the boot [56]. Plants of the Bowman backcross-derived line for *clh1.a* (BW182) were 30 to 40% shorter than Bowman. The kernels were slightly thinner and were about 2/3 normal weight. BW182 plants produced very little grain.

#### Hairy leaf and leaf sheath

The surface of a plant can be covered by a hair layer of trichomes. In leaves of *Arctotheca populifolia*, the hair layer is known to increase leaf temperatures and reduce transpiration with minimal reductions in photosynthetic rates [177]. Plant hairs are also known to act as a physical barrier protecting leaf surface from smaller insects [178].

**Fig. 29:**
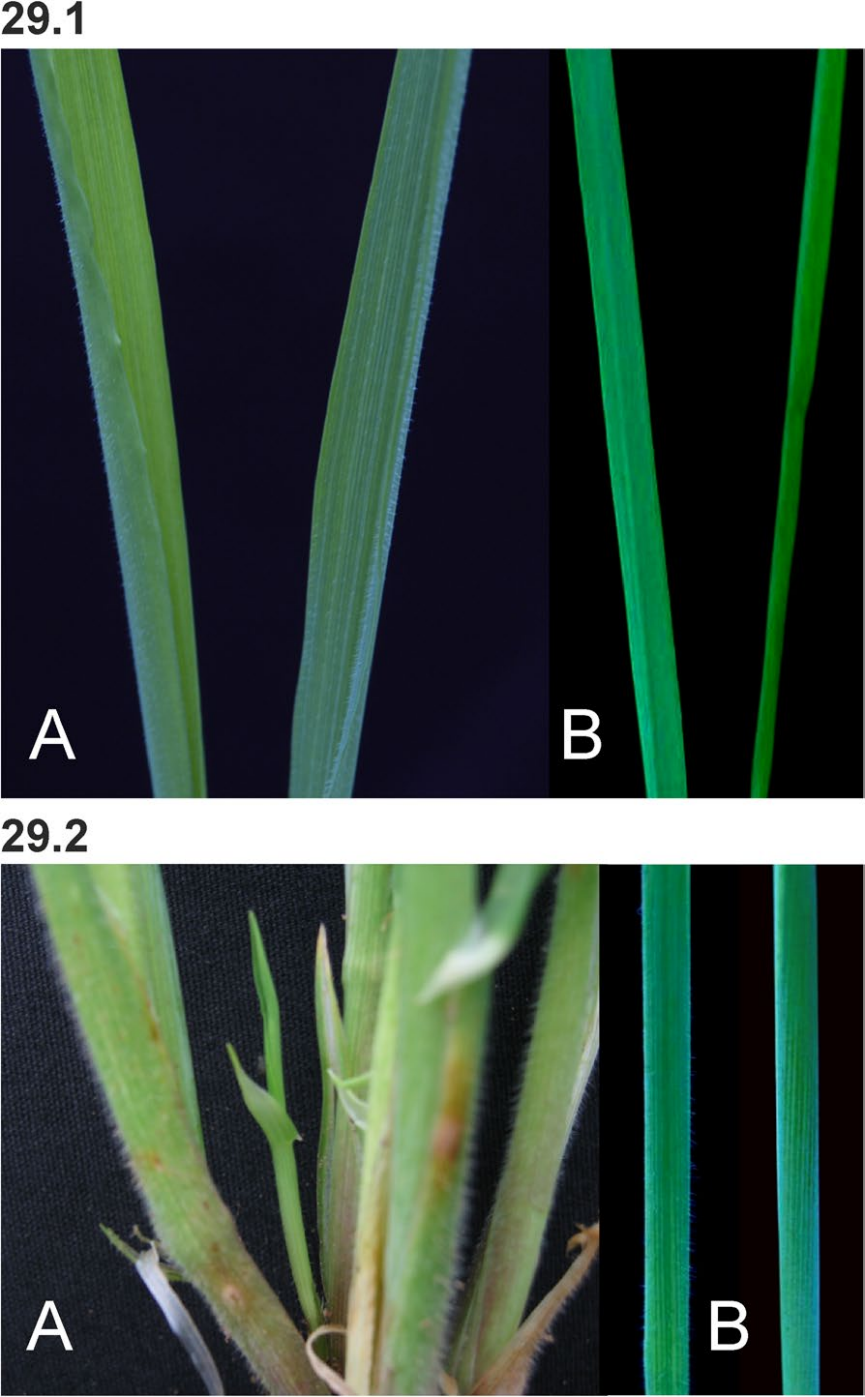
**29.1 A.** Pubescent leaf blade 1 (*Pub1.a*) with scattered small hairs on the leaf blade surface. **B.** A Bowman leaf blade without hairs. **29.2 A.** Basal part of a Hairy leaf sheath 1 (*Hsh1.a*) plant. **B.** Single basal culm of *Hsh1.a *to the left compared to Bowman

*Pub1.a* (Pubescent leaf blade 1) is a dominant variant found in most wild barley. Leaf blades have short hairs spread on both the upper and lower surface (Fig. 29 (29.1)). The hairs align along the smaller leaf veins, and hairs are easier to observe on younger leaves of plants grown in the field. Seeds of the Bowman backcross-derived line, BW650 (*Pub1.a*), weigh about 10% more than those of Bowman and grain yield is approximately the same [50]. Another dominant mutation causing hairiness is *Hsh1.a* (Hairy leaf sheath 1). *Hsh1.a* has short hairs on leaf sheaths of the basal part of the plant (Fig. 29 (29.2)). The density of hairs varies greatly between cultivars and depends on growing conditions. Generally, no hairs are observed on the sheath of upper leaves [101, 179]. Smooth awned cultivars appear to have fewer hairs [50]. *Pub1.a* and *Hsh1.a* are located on chromosome 3H and 4H, respectively, which demonstrates that there are at least two loci that regulate hairiness in barley.

### Kernel morphology and sterility

**Table:** 

Keywords to find descriptions of mutants in the International Database for Barley Genes and Barley Genetic Stocks (bgs.nordgen.org):
Shape of kernels: glo, globe shaped grain, globosum, lgk, long kernel, long shaped grain, seg, short kernel, shrunken endosperm
Hulless kernels: naked caryopsis, nud, semi-naked caryopsis, seminudoides, smn
Sterility: adjacent stigma, ajs, des, desynaptic, male sterile genetic, mov, msg, multiovary, ovaryless, ovl, partial male and female sterile, tfm, thick filament, tip sterile, tst, upper half spike sterile

The morphology of the barley kernel has intrigued barley breeders because the seed is the ultimate end product and thus directly connected to economic value. Barley grain is consumed as feed, food or beverages. For direct human consumption, hulless barley is preferred in which the hull, lemma and palea, do not adhere tightly to the caryopsis. In brewing, uniform size of the kernels is important in order to have an even and synchronized germination during malting. The goal is to obtain uniform and optimal conversion of starch to sugars.

#### Shape of kernels

**Fig. 30:**
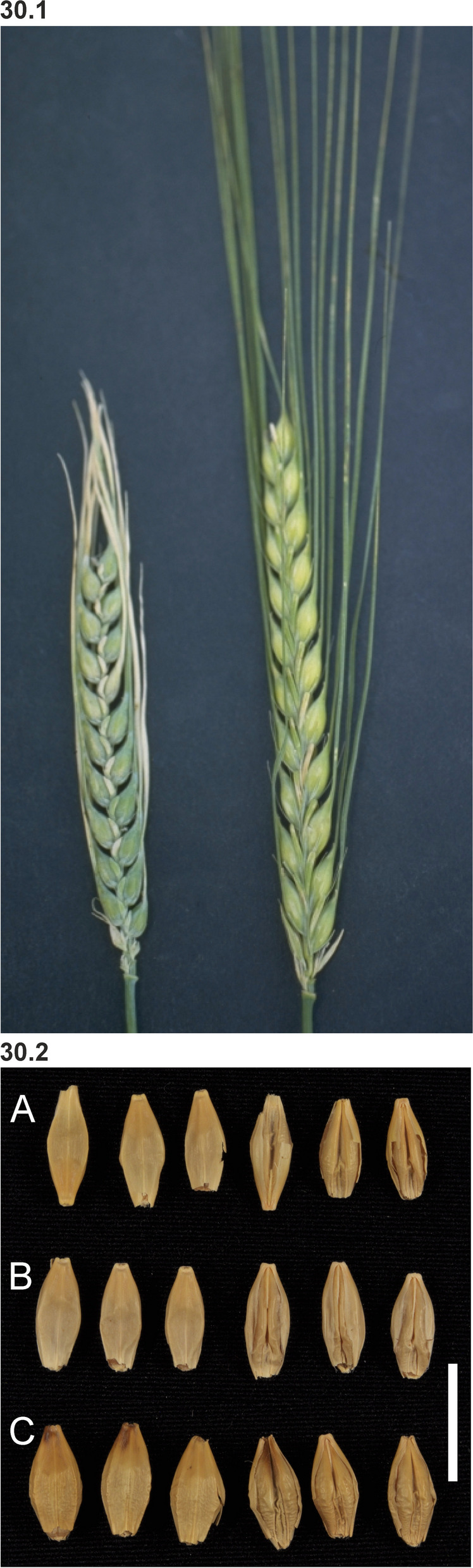
**30.1** Globosum-a (*glo-a.1003*) in a Bowman genetic background shows short and rounded kernels. Bowman to the right. **30.2 **Kernels of Shrunken endosperm genetic and Shrunken endosperm xenia mutants. The three plus three seeds in each group display the lemma side and palea side, respectively. **A.** Near-isogenic line BW836 *seg3.c*. **B.** Near-isogenic line BW844 *sex1.a*. **C.** Bowman. The bar corresponds to 1 cm

The barley kernel can show large morphological variation. In Globosum (*glo*) mutants, kernels are almost round or globe-shaped and kernel weight is lowered (Fig. 30 (30.1)). While there are single mutants at the *glo-a*, *glo-c*, *glo-e*, *glo-g*, *glo-h*, *glo-i* and *glo-j* loci, there are six *glo-b* and two *glo-f* mutants. The chromosomal locations are: *glo-a* (4H), *glo-b* (5HL), *glo-c* (2H), *glo-e* (3HL or 1HS), *glo-f* (5HL), *glo-g* (likely 2H), *glo-h* (7HS), *glo-i* (likely 7HS), *glo-j* (3HL or 5HL) [23, 175, 180, 181]. All mutations are associated with pleiotropic effects, which differ among mutant groups. Many mutants show a weak to strong reduction in plant height. In *glo-a.1003*, other spike tissues are reduced in length [182]. In contrast, *glo-c.1004* has a lax spike with 10–15% longer rachis internodes [175]. Mutant *glo-c.1004*, in a Bowman genetic background, is susceptible to lodging but it has normal grain yield [56].

In contrast to the round kernels of Globosum mutants, there are mutants with an inward curvature of the lemma, which is associated with reduced starch content in endosperm. Such mutants are Shrunken endosperm genetic (*seg1* to *seg9*) [183–186], high lysine (*lys1* to *lys6*) [187], and Shrunken endosperm xenia (*sex1, sex6* to *sex10*) [188]. Mostly there is just one allele known from each *seg* locus [184, 185, 188, 189]. The exceptions are two available alleles of *seg6* (*seg6.f* and *seg6.g*) and potentially many alleles of *seg3* since it was found that *seg3.c* is allelic to *ant17.148* [56]. There are more than 170 alleles of *ant17* [190]. Four of the *seg* mutants (*seg1, seg3, seg6* and *seg7*) showed premature termination of grain filling, leading to thin and wrinkled seeds (Fig. 30 (30.2)). The other four (*seg2, seg4, seg6,* and *seg8*) showed normal development of the seeds [191]. The central lobe of the endosperm fails to develop normally in the *seg8.k* mutant [192]. Regarding kernel size and weight, notably, *seg1* and *seg2* showed much reduced kernel size. The thousand-kernel weight was 33% and 15% of that of the cultivar Betzes, respectively. All *seg* mutants are of spontaneous origin from a large set of barley cultivars [183–186]. The *seg1, 2, 3, 4, 5, 6* and *8* loci are located on chromosomes 5H, 7HS (about 2.8 cM from *sex6*), 3HS, 7HL, 7HS (probably in the centromeric region), 3HL and 7H, respectively, while the location of *seg7* is unknown [23, 183, 184, 193–195].

Also, the Shrunken endosperm xenia (*sex*, Fig. 30 (30.2)) mutants are of spontaneous origin in many cultivars [183, 184, 196]. All mutant alleles are recessive. The loci *sex1, sex6, sex7, sex8* and *sex9* are located on chromosome 6HL, 7HS (2.8 cM from *seg2.b*), 5HL, 3HS and 4HL, respectively [23, 115, 197–199]. As with the *seg* mutants, *sex* mutant kernels are small and about 10–15% lighter than Bowman kernels. In addition, the kernels appear harder than normal. Notably, *sex6.h* kernels develop a depression in the center of the lemma which becomes progressively more distinct as maturity progresses. The mutant expresses xenia and kernels from heterozygous plants can be classified as normal or shrunken with an expected 3:1 ratio [182]. Defective endosperm xenia 1 (*dex1.a*, previously *sex2*, located on chromosome 5HS) is a recessive mutation that is expressed in the endosperm as a xenia effect [200]. The mutation causes a thin seed phenotype in heterozygous plants. In homozygous mutants, the seed stops growing a few days after fertilization, and it begins to shrivel resulting in a small seed that is barely visible within the lemma and palea.

#### Hulless kernels

**Fig. 31:**
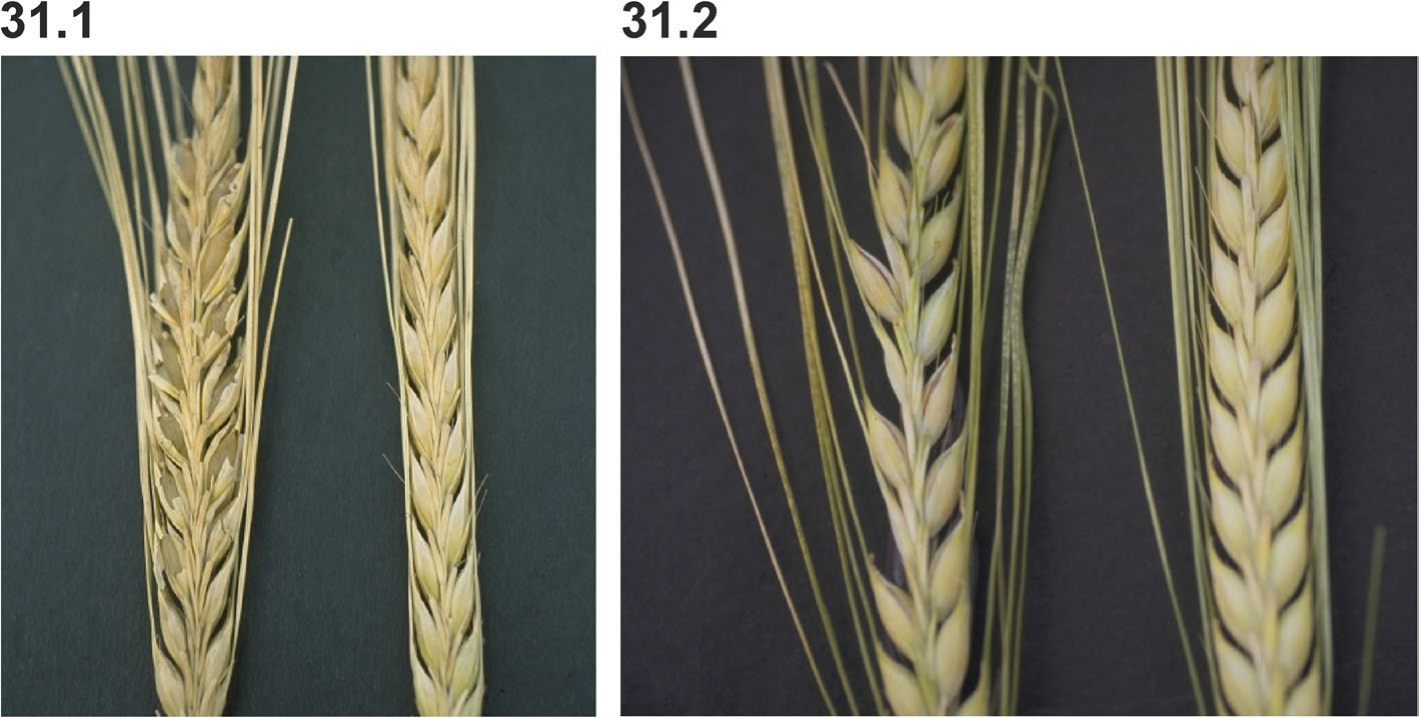
**31.1** In the barley Naked caryopsis 1 (*nud1.a*) mutant (left) the lemma and palea do not adhere to the caryopsis and the grain will thresh free of the hull at maturity. **31.2** The barley Seminudoides 1 (*smn1.a*) to the left compared with Bowman. There are gaps between the lemma and palea at maturity but the grain does not thresh free from the hull at maturity

Hulless kernels, so-called naked seeds, arose naturally during the barley domestication process and appeared as early as 9,000 years ago [201, 202]. A single recessive locus, Naked caryopsis 1 (*nud1*), controls the covered / naked caryopsis in barley (Fig. 31 (31.1)). The locus is found on the long arm of chromosome 7HL and encodes a transcription factor (ethylene response factor, *ERF*) involved in lipid biosynthesis [203]. The transcription factor is removed by a 17-kb deletion in mutant *nud1.a*. The *nud1.a* mutant phenotype is not affected by environment and *nud1.a* is often associated with Dense spike 1 (*dsp1*) in Japanese barley cultivars [204]. The kernel weights of the Bowman backcross-derived line for *nud1.a*, BW638, varied from 25% lower to almost equal, and grain yields ranged from 50 to 85% of those recorded for Bowman [50]. Wabila et al. [205] performed a genome-wide association study (GWAS) on a panel of 222 two-rowed and 303 six-rowed spring barley landrace accessions. It was concluded that the hulless phenotype is based on the 17-kb deletion in all accessions with one possible exception. In addition, to the well-described *nud1* locus three novel loci showed strong associations with the naked caryopsis trait. These loci were suggested to represent footprints of selection for naked caryopsis in different geographic areas rather than novel Naked caryopsis genes.

A partially naked seed is produced by Seminudoides (*smn1*, *smn2* and *smn3*) mutants. The caryopsis of these plants is not completely covered because gaps develop between the margins of the lemma and palea (Fig. 31 (31.2)). Adherence of the lemma and the palea to the pericarp is poor, but the grain does not thresh free from the hull at maturity. Tiller number and grain yield are often reduced [66]. The chromosomal location of *smn1* is 3H or 5H, *smn2* 3H and *smn3* 6HL [23]. Also in *lax-a* mutants the caryopses are exposed between the lemma and palea [206] and some waxless Eceriferum mutants as *cer-zv.268* are hulless [51].

#### Sterility

Male sterility mutants representing 49 loci have been isolated. They were named Male sterile genetic (*msg*) and are recessive mutations affecting the male genitals. Typically, the anthers are smaller than normal and their stomium can be absent. Often the filament does not fully elongate and the pollen displays a reduced size, are absent or are clumped. In contrast, the female fertility is not affected. Roath and Hockett [207] classified the mutants available at that time into four groups. Members of the first group, only represented by *msg9.ci*, have a low but reproducible degree of selfing ability in contrast to the other mutants which are not able to self. The second group, including *msg6.cf*, *msg8.ch*, *msg8.au*, *msg16.bi* and *msg16.co*, has almost normal anthers, stomium, filament and pollen. The third group is represented by *msg3.cc*, *msg7.ah*, *msg7.cg* and *msg7.fx*, and the fourth group by *msg1*, *msg2*, *msg4*, *msg5*, *msg10*, *msg11*, *msg11*, *msg13*, *msg14*, *msg17*, *msg18*, *msg19*. The stomium is absent in both the third and fourth groups and they do not show any elongation of the filament. In the third group, the pollen are very reduced in size or totally absent, and the anthers are very small and shrunken. In the fourth group, the mutants are characterized by pollen appearance from normal pollen to no pollen present, and anther sizes from slightly smaller than normal to very reduced and shrunken.

**Fig. 32:**
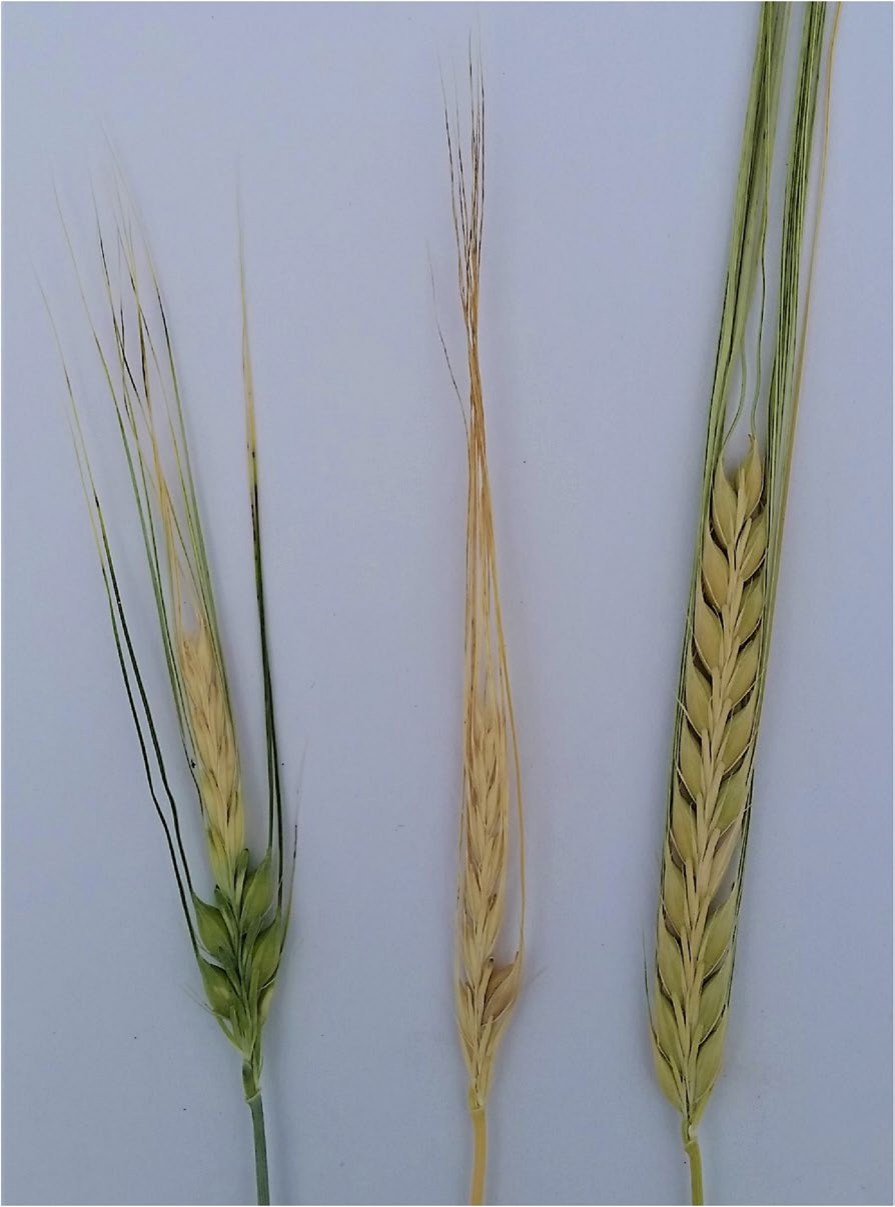
Two Tip sterile 2 (*tst2.b*) spikes to the left compared with Bowman

Another type of sterility mutants is Tip sterile 1 and Tip sterile 2. Only one mutant has been described from each locus, *tst1.c* and *tst2.b*, which were isolated after fast neutron treatment of Steptoe and x-ray irradiation of Donaria, respectively [55, 66, 208]. As the name implies, the mutants set seed at the lower part of the spike but are sterile in the upper part (Fig. 32). Obviously, grain yield is lower in the mutants although kernels of *tst1.c* in a Bowman genetic background were 10 to 15% wider than those of Bowman and weighed 10 to 15% more [55].

In the group of Desynapsis (*des*) mutants, the chromosome pairing (synapsis) during meiosis is disturbed [209]. The pairing of homologous chromosomes during the synapsis is crucial for enabling chromosomal crossover between them. In Desynapsis barley mutants, the pair of homologous chromosomes fails to maintain their physical association and thus separate prematurely. The chromosomes are paired in pachytene and undergo desynapsis in diplotene or early diakinesis [210]. The mutants differ in the amount of pairing observed at metaphase I. In barley, mutants have been described for 15 *des* loci. One or two alleles are available of each locus except *des4* where ten alleles are available. Plants of Desynapsis mutants are similar to normal plants, but grain yield is considerably reduced and kernels are often lighter [50].

Additional mutants, also affecting fertility are Multiovary (*mov*), Ovaryless (*ovl*), Eceriferum (*cer-yd.139* and *cer-yh.116*) and Thick filament 1 (*tfm1*). While carpels are absent or rudimentary in *ovl* mutants causing female sterility, stamens are converted into pistils in *mov* mutants [55, 211]. Selfed seed set is poor in the Bowman backcrossed-derived lines for *cer-yd.139* (BW135) and *cer-yh.116* (BW139) because stigmata have few hairs. In *tfm1.a*, the filaments supporting the anthers are much thicker than normal, having a stalk-like appearance and a light green color. In the Adjacent stigma 1 (*ajs1*) mutant, the stigma has an altered position, but does not reduce seed set [56]. Many of these mutants are sterile and the mutations must therefore be maintained in heterozygous seed stocks.

### Early and late flowering

**Table:** 

Keywords to find descriptions of mutants in the International Database for Barley Genes and Barley Genetic Stocks (bgs.nordgen.org):
Early flowering: eam, early maturity, mat, praematurum
Late flowering: lam, late maturity

The timing of the transition from vegetative to reproductive growth stages is not only a very important breeding trait but also of great importance for the fitness of plants. The transition should be optimized so the vegetative phase promotes an optimal number of plump seeds within the growing season. Thus, the plant should balance the possibility to stay in the vegetative phase and thereby being able to produce many plump seeds during the reproductive phase, without the risk of being unable to produce mature seeds before the growing season ceases.

#### Early maturity and late maturity mutants

**Fig. 33:**
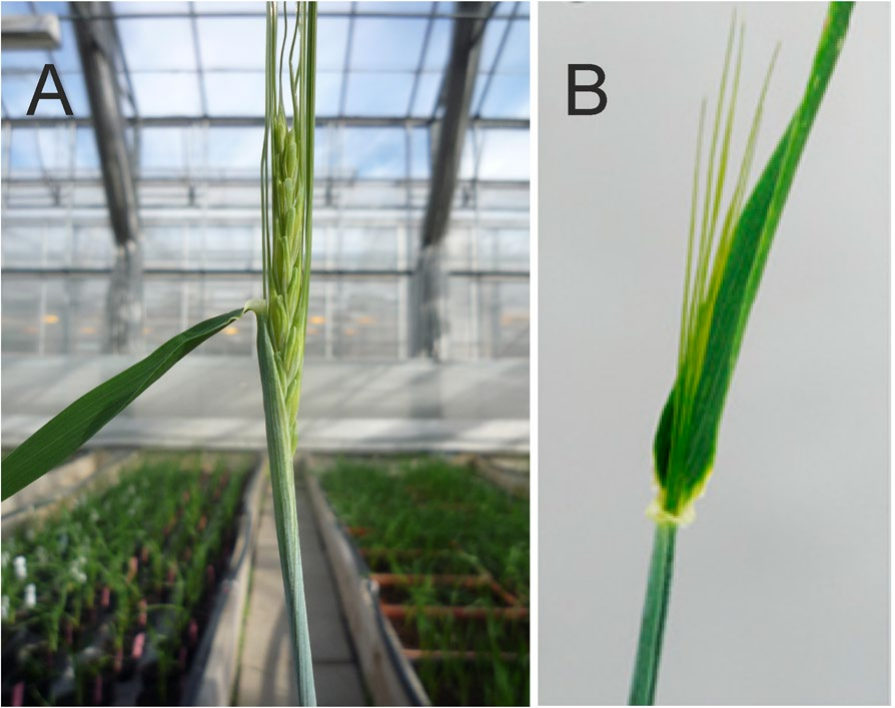
Day-of-heading and day-of-awn-appearance are often used to approximate the start of flowering in barley since they do not require dissection of the closed barley flowers. **A.** The day-of-heading is typically said to be the day when at least 50% of the first spike of a barley plant emerge above the flag leaf. **B.** Similarly, the day-of-awn-appearance is the day when approximately one cm of the awns is visible

It is a true challenge to determine the onset of flowering or anthesis in grass species. Therefore, flowering in barley has normally been approximated by day-of-heading or day-of-awn-appearance instead of examining flowers for their actual development. The day-of-heading has typically been the day when half of the spike protrudes halfway above the flag leaf. The day-of-awn-appearance is typically when one centimeter of awns is visible (Fig. 33).

In the 1940s, it was established that the timing of flowering in barley can be modified towards either late or early maturity by chemical or physical mutagenesis. While late maturity occurred more frequently in mutant populations, screening for early mutants was much easier [45]. Some 17 late maturity mutant accessions and more than 1200 early maturity mutant accessions have been collected by Scandinavian researchers. The early maturity mutants were named Praematurum (*mat*). Allelism tests using 195 of the early mutants distinguished nine complementation groups – *mat-a* to *mat-i*, which are all recessive [45]. The *mat-a*, *mat-b* and *mat-c* mutant groups are the largest, each nominally containing 30–40 different alleles [212–214]. These mutants are also the earliest flowering mutants closely followed by mutants *mat-e*, *mat-h* and *mat-i*. Mutants *mat-d*, *mat-f* and *mat-g* are only slightly earlier than their mother cultivars in Sweden [45], but the BW511 with *mat-f.23* was 7 days earlier than Bowman under short-day conditions. Drastically early mutants are easier to discover in a mutant population compared to slightly early mutants. This probably explains the large number of isolated *mat-a*, *mat-b* and *mat-c* mutant alleles.

Loci regulating timing of flowering have also been isolated by other researchers in Europe, North America and Asia. Also, in these cases early maturity was in focus and very few, if any, publications on late maturity mutants are available. Many of the early maturity alleles occur naturally in cultivars and landraces, but a few might also have been induced. These early maturity loci are Early maturity 1 (*eam1* or *Ppd-H1*), Early maturity 5 (*eam5* or *HvPhyC*), Early maturity 6 (*eam6* or *HvCen*), Early maturity 7 (*eam7*), Early maturity 8 (*eam8* or *HvELF3*), Early maturity 9 (*eam9*) and Early maturity 10 (*eam10*). *eam1*, *eam5* and *eam6* are dominant and *eam7*, *eam8*, *eam9* and *eam10* are recessive [50].

Through diallelic crosses it was established that *mat-a* mutants are allelic to *erectoides-o.16* (*ert-o.16*), initially sorted in a group of dense spike mutants [215], and to a series of *eam8* mutants originally characterized in Japan [216, 217]. As the *mat-a* mutants, *ert-o.16*, *eam8.q*, *eam8.r*, *eam8.s*, *eam8.u* and *eam8.v* are induced mutants, whereas *eam8.k* occurs naturally in the cultivars ‘Kinai 5’ and ‘Kagoshima Gold’, and *eam8*.w occurs naturally in ‘Early Russian’ [151].

The drastic early maturity mutant *mat-a.8* was isolated in 1951 after X-ray treatment of the cultivar Bonus [45]. Ten years later, it was released as a commercial cultivar under the name Mari [17]. In field trials in Sweden and under long day conditions in phytotron experiments, it is 8–10 days earlier than the mother cultivar Bonus (Dormling et al. 1966). In moderate day length, heading was found to be as much as three or more weeks earlier. Due to its photoperiod insensitivity, Mari can also be grown near the equator as a day-length neutral plant. At the same time Mari was used in Northern Scandinavia and on Iceland [218]. Indeed, breeders servicing a wide geographic range have frequently used Mari or its derivatives in their programs [214]. Environmental stability is now prioritized by global organizations who seek to maintain crop yields under increasingly variable climatic conditions, decreased inputs and expansion into marginal lands. Consequently, early maturity mutant alleles have once again emerged as a potentially valuable breeding trait.

### Epicuticular waxes

**Table:** 

Keywords to find descriptions of mutants in the International Database for Barley Genes and Barley Genetic Stocks (bgs.nordgen.org):
Increased amount of wax: rich wax coating, waxy spike, wxs
Reduced amount of wax: cer, eceriferum, gle, glf, glossy leaf, glossy node, glossy sheath, gsh, yellow node, ynd

**Fig. 34:**
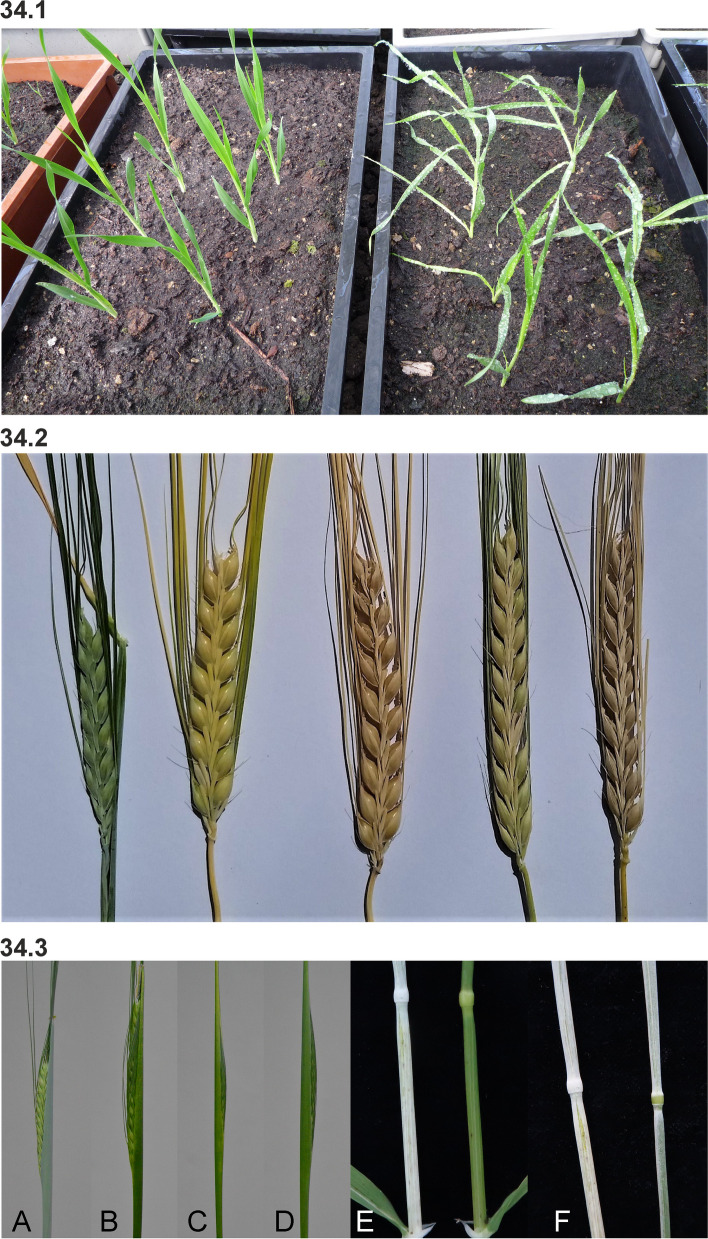
**34.1** Barley plants 30 minutes after being sprayed with water. The plants in the right box retain water droplets on their surface due to lack of epicuticular waxes. They are Glossy leaf 1 (*glf1.a*) leaf blade wax mutants (wax code ++ ++ -). (- = absent, + = reduced, and ++ = normal wax coating). **34.2** Three spikes of Waxy spikes 1 (*wxs1.a*) to the left compared with two spikes of normal Bowman at different stages of maturity. **34.3** Mutants with reduced epicuticular wax layers. **A.** Bowman with epicuticular waxes (wax code ++ ++ ++). **B.** Mutant *cer-c.36* (Eceriferum-c, - - ++). **C.** *cer-q.42* (- - ++). **D.** *cer-u.21* (+ + ++). **E.** Bowman to the left, *cer-b.2* to the right (- - ++). **F.** Bowman left, *cer-yr.492* right (-/+ + ++)

Most plant species produce a layer of wax on their organ surfaces. These epicuticular wax layers consist of complex mixtures of different substances such as acids, hydrocarbons, aldehydes, alcohols, esters, ketones, or combinations of them [219]. The wax layer plays a vital role in the protection of the plant against biotic and abiotic stress factors. In this way, the wax layer is a first defense barrier against insects and other pests and pathogens [220–222]. It also functions like a raincoat to repel excess water droplets from the leaf surface (Fig. 34 (34.1)). At the same time, the wax layer contributes to heat tolerance as it helps with water retention and prevents water evaporation from plant tissues [223–226]. Barley has a rich layer of epicuticular waxes on the spike, the leaf sheath, the leaf blade and the culm. Mutants can have an increased or decreased amount of wax. Mutations can affect one, several or all parts of the plant. The number of isolated mutants with a decreased amount of wax exceeds by far the number of mutants with increased amounts.

#### Increased amount of wax

Barley Waxy Spike 1 (*wxs1*) accumulates wax coating on the spike surface. Spikes appear normal at heading, but gradually they accumulate more surface waxes [55]. Spikes have a distinctive white hue prior to ripening (Fig. 34 (34.2)). Due to poor adherence of the lemma to the caryopsis, seeds have an irregular surface. The Bowman backcross-derived line BW917 (*wxs1.a*) is shorter and lodges easier than Bowman. It also yields about 30% less [55]. The four available mutants (*wxs1.a*, *wxs1.b*, *wxs1.c* and *wxs1.d*) have been assumed to be allelic based on phenotypic similarities, which has to be confirmed by diallelic crosses. It should be noted that mutants in the Gigas 1 (*gig1*) locus described above have a pronounced wax coating.

#### Reduced amount of wax

A total of 1,580 barley Eceriferum (*cer*) mutants with a reduced wax coating have been reported in the Scandinavian barley mutant collection [45, 227]. Due to the large number, a systematic approach was taken to group them into five different categories based on their wax coating on three different organs, i.e. the spike, the leaf sheath, and the leaf blade [228]. Special scoring symbols were used to classify the type of wax coating; “- “, wax coating is absent; “ + ”, wax coating is reduced; “ + + ”, normal wax coating (Table 3). When the wax coating is absent, the plant organs have a bright green (non-glaucous) color in contrast to the bluish (glaucous) color of a normal plant (Fig. 34 (34.3)). An intermediate color is found in plants with a reduced wax coating. The bluish color is due to β-diketone aliphatics and their hydroxyl- and oxo-β-diketone derivatives, which are the dominating waxes in barley [229]. Thanks to extensive allelism tests, 79 loci were identified in the collection [227]. Five or more alleles are present at 43 of the loci. The loci with most alleles are *cer-c*, *cer-q* and *cer-u* with 204, 156 and 147 alleles, respectively. Interestingly, these three genes form a closely connected gene cluster with 49.2 kb distance between *cer-q* and *cer-u*, and 46.5 kb between *cer-u* and *cer-c* [230]. Thus, it is not surprising that double and triple mutants with large deletions have been isolated from the *cer-cqu* cluster [231]. One Eceriferum locus, *cer-yy*, was represented exclusively by dominant mutations (19 alleles). Three additional dominant mutations have been reported from loci otherwise represented by many recessive mutations; *Cer-n.969*, *Cer-q.1440* and *Cer-t.977*.

**Table 3:** The five phenotypic categories of waxless mutants in the Scandinavian mutant collection with the numbers of loci and mutants. Mutant groups with five or more alleles are shown. (- = absent, + = reduced, and + + = normal wax coating)

Category of mutant	Spike	Leaf sheath	Leafe blade	No. of loci	No. of mutants	Mutant groups with 5 or more alleles (no. of mutants)
Spike and leaf sheath	-	-	+ +	8	533	*cer-a *(62)*, cer-b *(39)*, cer-c *(204)*, cer-q *(156)*, cer-x *(34)*, cer-z *(9)
Partial spike and leaf sheath	+	+	+ +	19	339	*cer-f* (5), *cer-g* (41), *cer-n* (56), *cer-r* (9), *cer-s* (19), *cer-u* (147), *cer-zi* (18), *cer-zu* (11)
Spike	-	+ +	+ +	23	294	*cer-d* (14), *cer-e* (45), *cer-h* (6), *cer-i* (68), *cer-o* (5), *cer-t* (50), *cer-v* (6), *cer-w* (19), *cer-yc* (5), *cer-yt* (11), *cer-yy* (19), *cer-zb* (6), *cer-zc* (16), *cer-zn* (6), *cer-zo* (5)
Leaf blade	+ +	+ +	-	25	390	*cer-j* (69), *cer-p* (37), *cer-yb* (5), *cer-ye* (5), *cer-yj* (5), *cer-ys* (5), *cer-yu* (7), *cer-za* (79), *cer-zd* (7), *cer-ze* (72), *cer-zh* (10), *cer-zj* (58), *cer-zp* (7)
Spike, leaf sheath and leaf blade	-	-	-	4	24	*cer-zk* (17)

Additional waxless mutants have been isolated in other laboratories and diallelic crosses have shown, which are allelic to the Scandinavian Eceriferum mutants (Table 4). Glossy sheath (*gsh*) mutants are allelic to mutants in the groups of spike and leaf sheath mutants (-—+ +) and partial spike and leaf sheath mutants (+ + + +). Similarly, Glossy leaf (*glf*) mutants are allelic to leaf blade mutants (+ + + + -). The Yellow node 1 mutant, *Ynd1.a*, carries a dominant mutation, but a few *cer* mutants also reduce the amount surface wax on nodes. In homozygous *Ynd1.a*, nodes of the culm appear to lack a coating of surface waxes. In heterozygotes, surface waxes may be observed on the upper half of the node. This trait is easier to observe in greenhouse grown plants where surface waxes are not rubbed off by wind agitated leaf or other parts of the plant. The *Ynd1.a* allele is present in many six-rowed cultivars of Oriental origin, as explained by a close linkage of *Ynd1.a* to the dominant Intermedium spike-c allele *Int-c.a*.

**Table 4:** Described waxless mutants outside the Scandinavian mutant collection. Crosses revealed allelism to Eceriferum (*cer*) mutants of the Scandinavian mutant collection

Category of mutant	Number of mutants	Corresponding *cer* loci	Reference
*gsh1*	4	*cer-q*	[232]
*gsh2*	5	*cer-b*	[232]
*gsh3*	3	*cer-a*	[232, 233]
*gsh4*	1	*cer-x*	[232, 233]
*gsh5*	1	*cer-s*	[233]
*gsh6*	14	*cer-c*	[234]
*gsh7*	1	-	[234]
*gsh8*	2	*cer-u*	[234]
*glf1*	4	*cer-zh*	[235]
*glf3*	2	*cer-j*	[77, 236]
*ynd1*	1	-	[50]

### Pigmentation

**Table:** 

Keywords to find descriptions of mutants in the International Database for Barley Genes and Barley Genetic Stocks (bgs.nordgen.org):
Chlorophyll mutants: abo, alb, albina, albino seedling, alboviridis, alboxantha, avi, axa, chlorina, chlorina seedling, clo, dark green leaf blade, fch, gpa, grandpa, leaf blade changing into yellow, lgn, light green foliage, lsc, lutescens, maculata, mcl, mid-season stripe, mottled leaf blade, mss, mtt, pgn, str, striata, tig, tigrina, val, var, variegated, vir, virescens, viridis, virido-albina, vsc, white spotted leaf blade, white streak, wls, wst, xal, xan, xantha, xantha seedling, xanth-alba, xnt, yellow leaf, yellow leaf blade, yellow streak, ylf, yst, zeb, zebra stripe, zon, zonata
Anthocyanin and proanthocyanidin mutants: ant, anthocyanin-less, anthocyanin-rich, proanthocyanidin-less, purple veined lemma, Pvc, Red stem, Rst
Necrotic spot and blotch mutants: mac, maculosus, ncd, nec, necroticans, pmr, premature ripe
Changed pigmentation in spikes: albino lemma, alm, black lemma and pericarp, blp, blue aleurone xenia, blx, bnk, brown kernels, dark grain, dsk, dusky, ebu, eburatum, fla, flavum, ibl, intense blue aleurone, orange lemma, pink aleurone, pre, purple lemma and pericarp, rob, robiginosum, yaw, yellow awn, yellow head, yhd

Chlorophyll is the dominating pigment in plants and deficiency in chlorophyll biosynthesis is a common cause of many of the pigmentation mutants isolated in barley. Some mutations have been traced to the chlorophyll biosynthetic pathway itself and those that affect entire plants are often lethal. Others have an impact on a specific part of the plant and are seen at a certain developmental stage. Those mutations are likely to be in genes regulating chlorophyll biosynthesis directly or indirectly. It should also be noted that chlorophyll biosynthesis and chloroplast development are mutually connected and many of the internal structures of the chloroplast are absent, repressed or underdeveloped in chlorophyll biosynthetic mutants and vice versa [237, 238].

Flavonoids are the most numerous secondary metabolites in plants, fulfilling a wide range of functions from flower coloration to UV filtration [239–241]. Anthocyanins and proanthocyanidins are examples of flavonoids and a large number of barley mutants affected in these metabolites have been isolated. There are also additional mutant classes with a considerably smaller number of available mutant accessions. Those cause necrotic spots mainly on leaves or changed pigmentation of spikes.

#### Chlorophyll mutants

Chlorophyll mutants were probably among the first barley mutants to be studied. They are the most common mutants that appear after mutagenesis. Due to their distinct color changes at an early seedling stage, they were used as indicators for the success of the mutagenic treatment [45]. Based on their different colors and whether the mutations were lethal or not, the chlorophyll mutants were classified into different categories (Table 5) [4]. Early experiments with induced mutagenesis revealed that the frequency of the various chlorophyll mutant classes varied with different treatments such as irradiation doses and water content of the treated seeds. For example, the frequency of *albina* mutants is high at low irradiation dosages of water-soaked seeds, while *xantha* mutants are most easily produced at relatively high dosages. The transversely striped *tigrina* mutants were rare and only obtained by irradiation of dry seeds [4, 11, 242].

**Table 5:** The different types of chlorophyll mutants are based on their coloration. Lethal mutations are maintained in heterozygous stocks

Type	Gene symbol	Characteristic	Viability
Albina	*alb* (Scandinavia), *abo* (USA)	White seedlings without chlorophyll or carotenoids	Lethal
Xantha	*xan* (Scandinavia), *xnt* (USA)	Yellow seedlings with carotenoids but without or with very reduced levels of chlorophyll	Lethal
Viridis	*vir*	Yellowish green or light green at seedling stage	Lethal
Chlorina, Chlorina seedling, Light green foliage	*clo* (Scandinavia), *fch*, *lgn* (USA)	Yellowish green or light green	Viable
Virescens	*vsc*	Yellowish green or light green at seedling stage. Turn darker green when older	Viable
Lutescens	*lsc*	Darker green at seedling stage. Turn yellowish green or light green when older	Viable
Alboxantha	*axa*	Tip white, base yellow	Lethal
Xanth-alba	*xal*	Tip yellow, base white or faintly colored	Lethal
Alboviridis	*avi*	Tip white, base green or yellowish green	Lethal
Virido-albina	*val*	Tip more or less greenish or yellowish green, base white	Lethal
Tigrina	*tig*	Transverse stripes with destructed tissues	Lethal
Zonata	*zon*	Transverse stripes with white or yellow colors	Lethal
White spotted leaf blade, Mottled leaf blade, Zebra stripe	*mtt, zeb*	Transverse stripes with white or yellow colors	Viable
Striata	*str*	Longitudinal stripes of white or yellow colors	Viable
Mid-season stripe, Yellow streak, White streak, Variegated	*mss, yst, wst, var*	Longitudinal streaks of white or yellow color	Viable
Maculata	*mcl*	Chlorophyll and carotenoid destruction in the form of white dots on leaves	Lethal

**Fig. 35:**
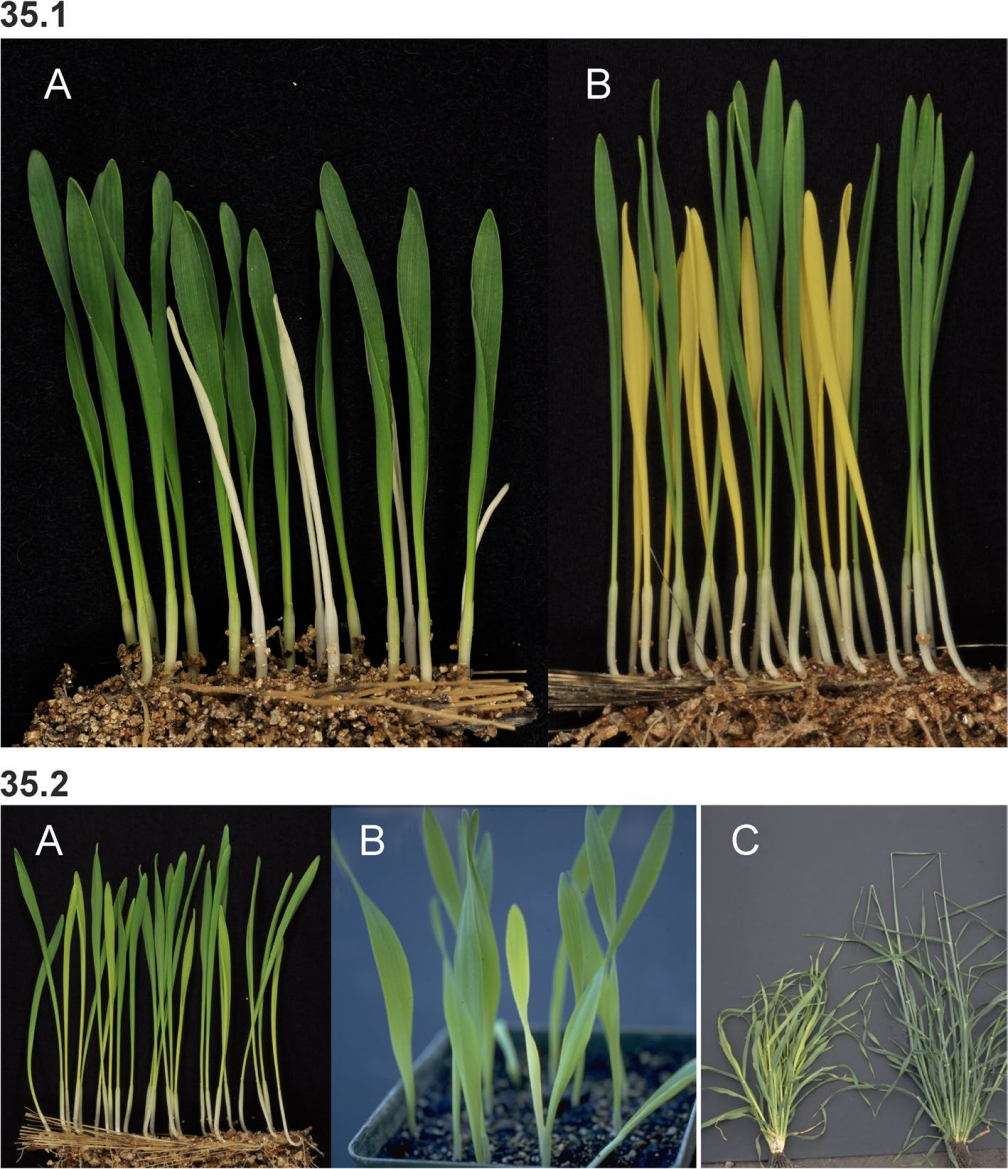
**35.1** Seedling phenotype of segregating white Albina (**A**, *alb-d.15*) and yellow Xantha (**B**, *xan-g.65*) mutants. Homozygous Albina and Xantha mutants are white and yellow, respectively. **35.2** Examples of barley mutants with a light green color due to a low but visible amount of chlorophyll. **A.** Segregating Viridis mutants (*vir-s.44*). The light green homozygous *vir-s.44* seedlings are not able to set seeds since Viridis mutations are lethal in contrast to vital Chlorina mutations. **B.** A mutant seedling of Chlorina seedling 12 (*fch12.b*) surrounded by darker green wild-type seedlings. **C.** Mutant *fch12.b* is also possible to distinguish from wild-type plants at later stages of development, which is often more complicated with other light green chlorophyll mutants

The Albina and Xantha mutants are white and yellow, respectively (Fig. 35 (35.1). The Albina mutants are white since they do not produce chlorophyll nor carotenoids or contain very low amounts of these compounds. The Xantha mutants can still synthesize the yellow carotenoids, but they are low in chlorophylls. Mutants were generally classified after visual inspection of plants grown in fields and greenhouses and only later analyzed by spectrophotometric methods where trace amounts of the pigments could be detected [237, 243]. Other mutants, named Viridis, Chlorina, Chlorina seedling and Light green, display a visible amount of chlorophyll and show a yellowish-green or light-green phenotype (Fig. 35 (35.2)). The Albina, Xantha and Viridis mutations are lethal, and the homozygous mutants die at the seedling stage when the energy resources from the kernels have been depleted. Still, the possibility to obtain a lot of plant material from barley seedling leaves also carrying lethal chlorophyll mutations is an advantageous possibility for molecular and biochemical experiments which cannot be provided in for example Arabidopsis, which has seeds with very small energy reserves. Due to the lethal nature of the mutations, the Albina, Xantha and Viridis mutations must be kept in heterozygous stocks and each experiment has to be preceded with phenotypic sorting of segregating populations. The Chlorina mutants are yellowish green or light green like the Viridis mutants but are viable and thus can be kept as homozygous stocks. It should however be noted that their yellowish-green or light-green phenotype is best observed at the seedling stage and can often be hard to distinguish from wild type in more mature plants.

Plants with Virescens and Lutescens mutations change color over time. Young Virescens mutants are yellowish green or light green at the seedling stage but turn darker green at later stages. On the other hand, Lutescens mutants obtain a lighter green color when aging.

Other variants of chlorophyll mutants are the Alboxantha, Xanth-alba, Alboviridis and Virido-albina mutants. As their names indicate, they are two-colored with the first part of the name referring to the tip of the seedling leaf and the second half referring to the basal part. For example, an Alboxantha mutant has a white tip and a yellow base (Table 5).

**Fig. 36:**
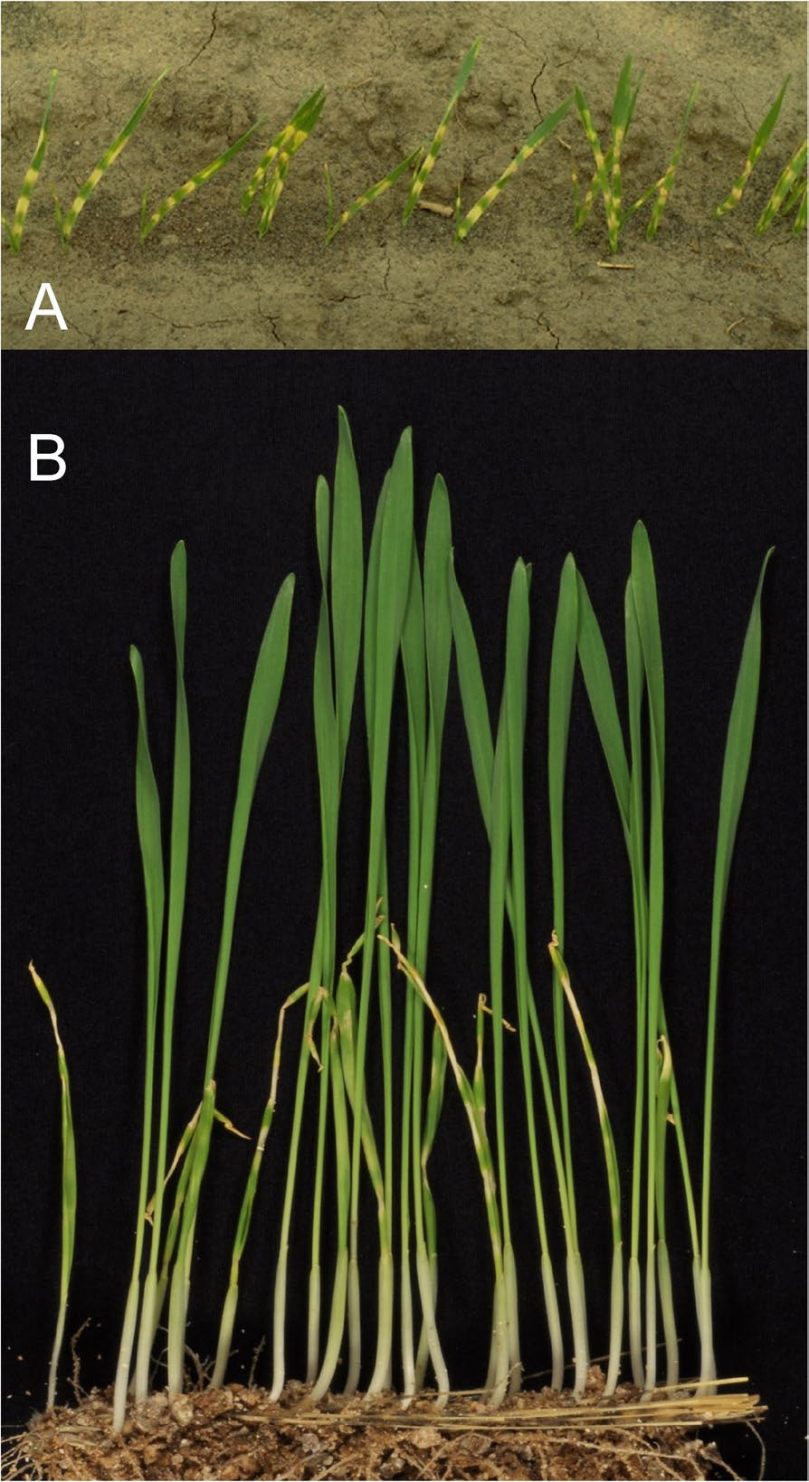
Transversally striped barley mutants. The stripes correspond to tissues elongated during the light/dark cycles of the day. **A.** Mutant Zebra stripe 1 (*zeb1.a*) grown in the field. **B.** Segregating seedlings of a Tigrina mutant (*tig-a.6*) grown under light/dark cycles. The green parts correspond to leaf segments elongated in the light, while pale necrotic bands correspond to segments elongated during the dark. When segments developed during the dark phase are illuminated during the light phase they degrade

Chlorophyll mutants can also be striped as seen in Tigrina, Zonata, White spotted leaf blade, Mottled leaf blade, Zebra stripe, Striata, Mid-season stripe, Yellow streak, White streak, and Variegated mutants. The variegated phenotype of these plants, with areas of normal chlorophyll biosynthesis, suggests these mutations to be in regulatory genes rather than biosynthetic genes. Transverse stripes are displayed in Tigrina, Zonata, White spotted leaf blade, Mottled leaf blade and Zebra stripe mutants where green bands alternate with necrotic bands in the case of Tigrina and whitish or yellowish bands in the case of Zonata, White spotted leaf blade, Mottled leaf blade, and Zebra stripe mutants. The bands have a circadian appearance and correlate with dark/light cycles (Fig. 36). Mutant *tig-d.12* accumulates an excess of the chlorophyll biosynthetic intermediate protochlorophyllide when grown in the dark [244–246]. Protochlorophyllide causes the formation of reactive oxygen species upon illumination, which causes necrosis. In constant light, the *tig-d.12* plants are uniformly green and can be grown to maturity.

**Fig. 37:**
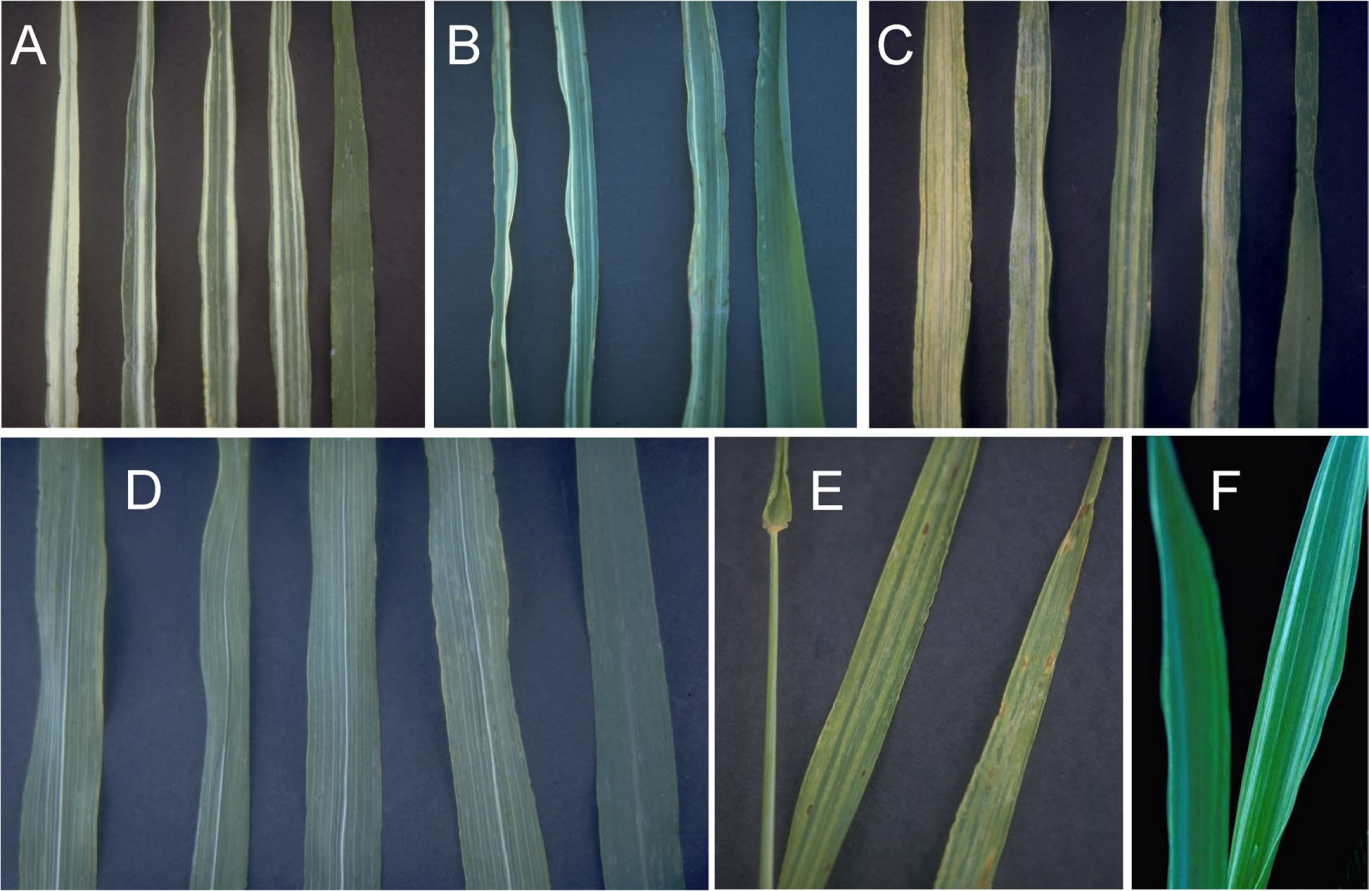
Example of longitudinally striped barley mutants. **A-D.** Four mutant leaves to the left, one wild-type leaf to the right. **A.** Midseason stripe 2 (*mss2.b*). **B.** White streak 7 (*wst7.k*). **C.** Yellow streak 3 (*yst3.c*). **D.** Yellow streak 5 (*yst5.e*). **E.** Yellow streak 2 (*yst2.b*). **F.** Varigated 2 (*var2.b*)

Longitudinal striped mutants have been named Striata, Mid-season stripe, Yellow streak, White streak, Grandpa, and Variegated (Fig. 37). The distribution and emergence of the stripes varies between mutants. For example, the phenotypic expressions of several mutations are temperature sensitive. While *mss2.b* (Midseason stripe 2) displays few or no stripes in cool environments, numerous white stripes develop in warm environments. In contrast, *wst7.k* (White streak 7) and *yst2.b* (Yellow streak 2) have fewer streaks and fewer tillers with streaks as environmental conditions become warm. The streaks can also arise at different developmental stages of the plant. In mutant *yst5.e*, beginning with the second leaf, emerging leaf blades are very pale yellow green in color. As the leaf blade matures, fine vertical green streaks develop. More greening of the leaf blade occurs along the midrib than near the margins. This pattern of greenish streaks in a yellow background persists until heading. Then leaf blades gradually develop a normal green color.

#### Anthocyanin and proanthocyanidin mutants

**Fig. 38:**
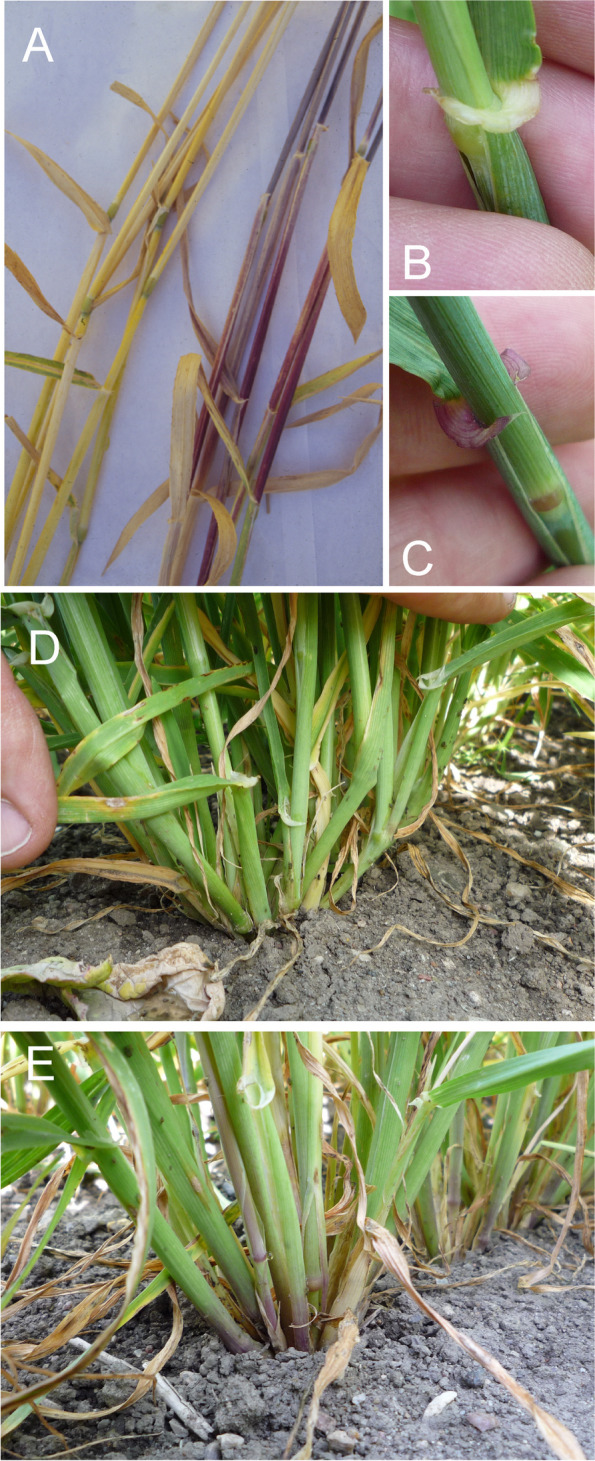
Barley anthocyanin-less mutants. **A.** Bowman near-isogenic lines carrying the mutant allele *ant1.b* (left) and the wild-type allele *Rst1.a* (right) of the *Ant1* locus. The recessive *ant1.b* (also called *rst1.b*) allele has a natural occurrence in cultivars like Bowman and Morex and can be traced through their pedigrees to Manchurian-type cultivars [61]. **B.** Anthocyanin-less auricle of *ant1.2*. **C.** Anthocyanin containing auricle of cultivar Bonus, which is the mother cultivar of *ant1.2*. **D.** Culm basis of *ant1.2*. **E.** Culm basis of Bonus

Anthocyanins and proanthocyanidins are examples of flavonoids, which are the most numerous secondary metabolites in plants. They are based on a common C_15_ carbon skeleton, which is derived from phenylalanine in a complex biosynthetic pathway [247, 248]. Both anthocyanin and proanthocyanidin are present in barley and share the biosynthetic pathway up to leucocyanidin. Anthocyanin is less visible than chlorophyll but is often seen as a purple color at the leaf blade, leaf sheath, auricle, kernel, awn, node and at the culm base of the barley plant [249]. Cold temperature and strong light are often needed to induce the synthesis of anthocyanin. Inability to synthesize anthocyanin results in absence of the purple color (Fig. 38). Proanthocyanidin is colorless but has attracted much interest among barley breeders because colloidal haze in beer is caused by proanthocyanidins precipitating malt proteins. Mutants blocked in biosynthesis of proanthocyanidin show excellent haze stability [250]. Physical mutagenesis with alpha-particles or neutrons led to the isolation of anthocyanin-free barley mutants already in 1952 [42]. The first proanthocyanidin-free barley mutant was isolated in 1974 [251]. More than 900 *ant* mutants have been isolated. Of these, 566 have been assigned to 31 loci through allelic crosses [45].

#### Necrotic spot and blotch mutants

**Fig. 39:**
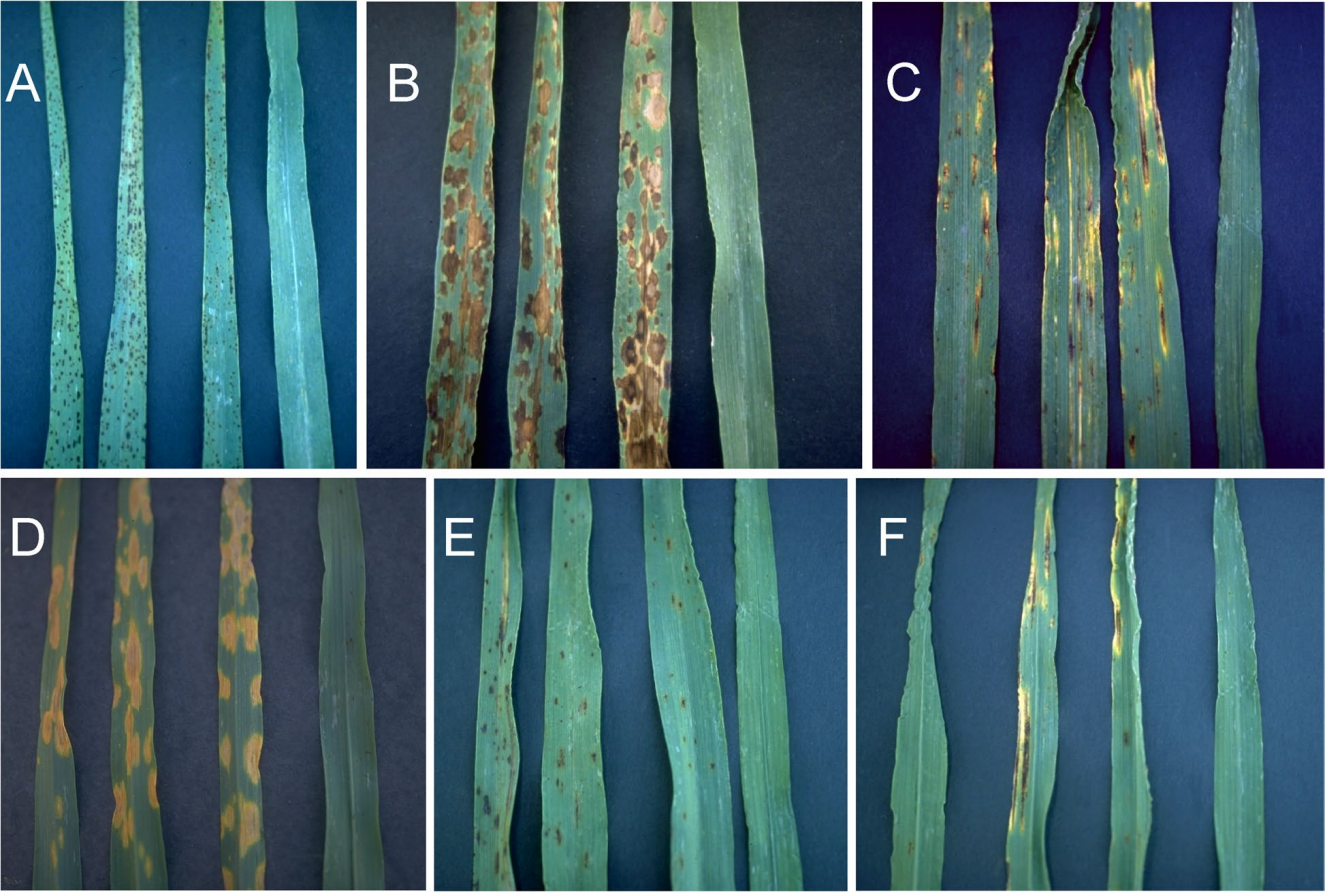
Barley mutants with necrotic spots. Each photo shows three mutant leaves to the left and one wild-type leaf to the right. **A.** Necrotic leaf spot 1 (*nec1.a*). **B.** Mutation *Nec6.h* is a dominant mutation. **C.** *nec7.45*. **D.** *nec3.e*. **E.** *nec.60*. **F.** *nec.39*

Necrotic spot mutants have attracted attention because they sometimes show resistance to various diseases without having pleiotropic negative impacts on agronomic performance and vigor. For example, allelic mutants of Necroticans 10 (*nec10*) show enhanced resistance to *Puccinia graminis* f. sp. *tritici* races MCC and QCC, and *P. graminis* f. sp. *secalis* isolate 92-MN-90, but not to stripe rust (*Puccinia striiformis* f. sp. *hordei*) [252]. The spots are often oval with the longest dimension parallel to the leaf veins. The Necroticans mutants are more severe than the Maculosus mutants, i.e. Necroticans display more dead tissues in the spots. The color of the spots is black/dark brown to light brown/yellowish. The size of the spots varies from less than one millimeter in diameter to cover most of the leaf blade width. The margins of the spots can be very sharp and distinct, but sometimes they display a dark center and a broad yellow margin (Fig. 39). The spots are not always restricted to the leaf blades, but they can appear also on leaf sheathes, culm internodes and awns [253, 254]. Plants with necrotic mutations commonly have a wild-type phenotype at the seedling stage and start to develop spots at later growth stages. In mutants carrying the dominant allele *Nec6.h*, the spots appear on the first leaf when seedlings are at a three to four-leaf stage and on succeeding leaves when the leaf blades have partially expanded. In the recessive mutant *nec7.45*, dark brown blotches become visible on the leaf blades and sheaths as each tiller of plants homozygous for the allele starts to head. They gradually enlarge and partially coalesce.

#### Changed pigmentation in spikes

**Fig. 40:**
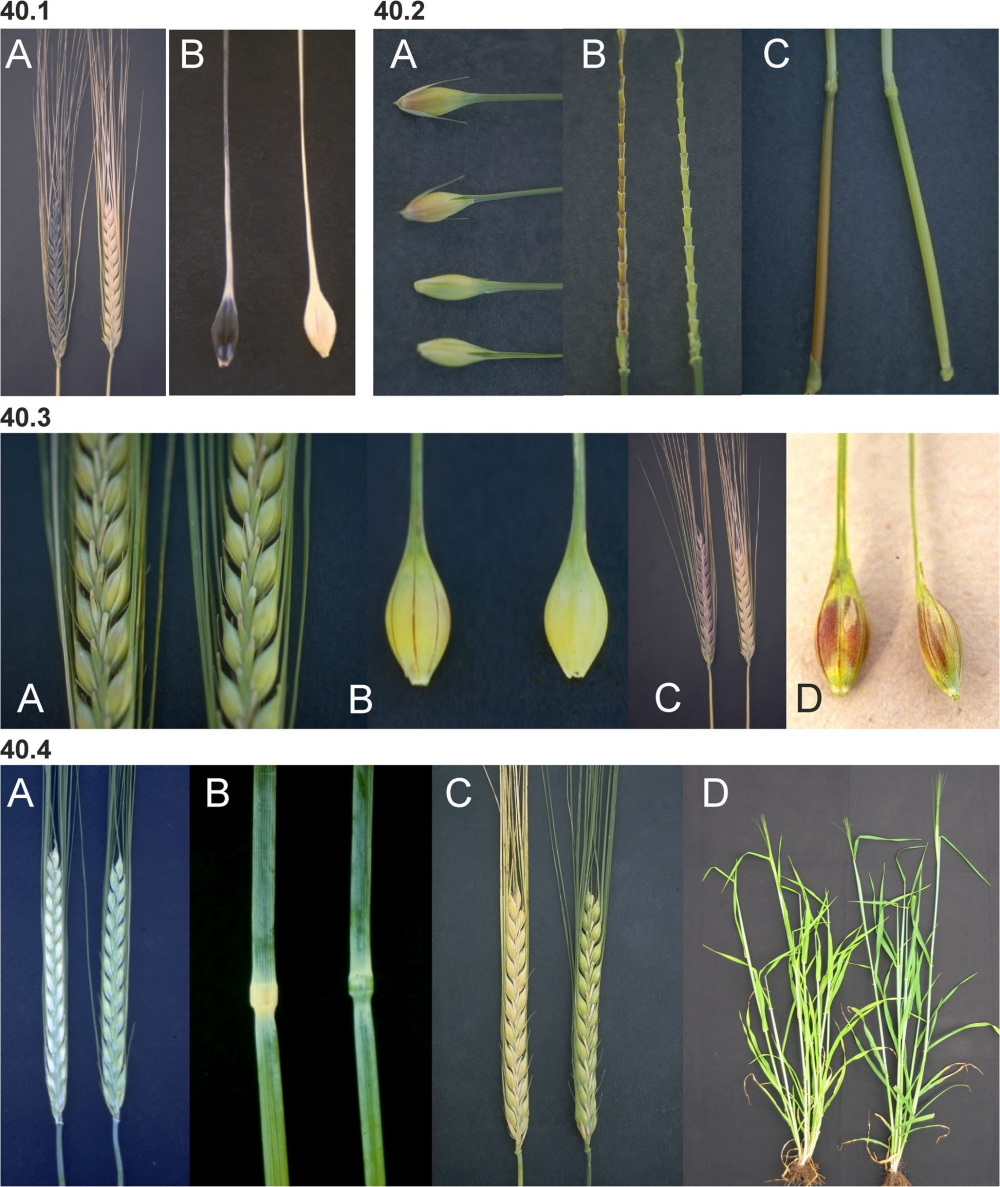
**40.1** Black pigmentation of barley mutant Black lemma and pericarp 1 (*Blp1.b*) (left) compared to cultivar Bowman (right). **A.** Spikes. **B.** Seeds. **40.2** Barley mutant Orange lemma 1 (*rob1.a*). **A.** An orange-colored pigmentation can be seen on kernels. Two mutant seeds at the top, two Bowman seeds at the bottom. **B.** Rachis of *rob1.a *left, Bowman right. **C.** Culm internodes of *rob1.a *left, Bowman right. **40.3** Barley mutants with changes in spike color due to increased levels of anthocyanin. **A and B.** Purple veined lemma 1 (*Pvc1.a*) to the left and Bowman to the right. **C.** Red lemma and pericarp 2 (*Pre2.b*) to the left and Bowman to the right. **D.** Two kernels of *Pre2.b*. **40.4** An example of barley mutants with a changed pigmentation in spikes due to a low amount of chlorophyll. **A and B.** Albino lemma 1 (*alm1.a*) to the left compared to Bowman. **C.** Spike of Yellow head 1(*yhd1.a*, left). **D.** The yellow-green phenotype of Yellow head 2 (*yhd2.b*, left) is possible to distinguish also at heading

Mutants with altered pigmentation of spikes form a diverse group. A striking pigmentation pattern is found in Black lemma and pericarp 1 (*blp1*) mutants. As spikes mature, seeds are colored black by a melanin-like pigment [94], which starts to develop in the lemma and pericarp slightly before maturation of the spike (Fig. 40 (40.1)). The pigmented organs may include all parts of the spike, awns, upper portions of the stem, and upper leaves. The intensity of the pigmentation varies between the different dominant alleles of the *blp1* locus – the *Blp1.b* allele confers extreme black pigmentation, the *Blp1.mb* allele is associated with medium black and a reduced distribution pattern, and the *Blp1.g* allele is associated with light black or gray coloration [255, 256].

A weak orange pigmentation of the lemma, palea and rachis is found in Orange lemma 1 (*rob1*) [256, 257]. The orange pigmentation is also visible at the base of the sheath of seedlings and in exposed nodes after jointing. Internodes have a layer of orange tissue and stems have an orange color as the straw dries (Fig. 40 (40.2)). The Orange lemma mutants are low in lignin due to mutations in the gene encoding cinnamyl alcohol dehydrogenase, which is the last enzyme in the lignin biosynthetic pathway [258].

Anthocyanins can also alter coloration of the spikes. In mutant Purple veined lemma 1 (*pvc1*), the purple pigment is confined mainly to the lemma veins, whereas the pigment is more widely spread to the lemma, palea and pericarp in Red lemma and pericarp 1 and 2 (*pre1* and *pre2*) [94] (Fig. 40 (40.3)). The common alleles in the *pvc1*, *pre1* and *pre2* loci are dominant, and the pigments are formed late during grain filling when the kernels are exposed to sunlight. The pigments tend to fade as the spike matures and therefore the mutants cannot be identified as lacking anthocyanidins and reddish coloration.

A blue color, due to anthocyanin pigments, is seen in aleurones of barley plants carrying recessive mutations of Non-blue aleurone xenia 1 (*blx1*) [259]. The anthocyanin pigments occur as lumps inside many aleurone granules in some or all aleurone cells [260]. Variation in blue color expression from dark blue to off-white is caused by environmental factors and modifying genes such as Intense blue aleurone 1 (*ibl1*). Aleurone color is best observed in well-filled grain that is magnified to show individual aleurone cells, after more external tissues have been peeled off [261].

Absence of chlorophyll is another cause of changed pigmentation in the spikes. In the Albino lemma 1 (*alm1*) mutant, the lemma and palea are white in color and mostly devoid of chlorophyll, but they terminate into green tips with green awns. The basal part of lower leaf sheaths and stem nodes are devoid of chlorophyll (Fig. 40 (40.4)). The immature spikes of the Yellow head 1 (*yhd1*) mutant appear ivory to pale yellow in color. The lemma is ivory-colored but terminates into green tip with green awns. The plant has a whitish lower leaf sheath and ivory-colored culm nodes and rachis internodes. The pattern of reduced chlorophyll development is similar to the *alm1* mutants (Fig. 40 (40.4)). The yellow-green phenotype of the Yellow head 2 (*yhd2*) mutant is very similar to that expressed by some of the Chlorina mutants (Fig. 40 (40.4)). It is possible that the *yhd2* mutant could be grouped with the Chlorina mutants. Anthers and plants of the Dusky 1 (*dsk1*) mutant remain green at maturity because gradual loss of chlorophyll during maturation does not occur.

## How to use the bgs database

The present review is tightly linked to the International Database for Barley Genes and Barley Genetic Stocks (bgs.nordgen.org) where further details and images of each mutant described in this review can be found. In the search tool at the main page, any word (i.e. gene, locus or phenotype) given at the start of each subchapter in this review can be written, which further links to a page with more extensive information of a mutant group. It is also possible to search for a given BGS number. Further, the top menu also has links to a “BGS table” and a “Locus table”. The BGS table provides a list of all current BGS descriptions from BGS1 to BGS831. The Locus table gives an alphabetic list of all loci for which there is a BGS description. A link to “Background data” is also available at the top menu. This is a database of more than 10,000 Swedish barley mutants concerning their year of isolation, used mutagen, mother cultivar, allelic identity (when known) and inheritance pattern.

## Data Availability

Not applicable.

## References

[1] Mascher M, Gundlach H, Himmelbach A, Beier S, Twardziok SO, Wicker T (2017). A chromosome conformation capture ordered sequence of the barley genome. Nature..

[2] Hansson M, Komatsuda T, Stein N, Muehlbauer GJ, Stein N, Muehlbauer GJ (2018). Molecular mapping and cloning of genes and QTLs. The barley genome.

[3] Gustafsson Å (1938). Studies on the genetic basis of chlorophyll formulation and the machanism of induced mutatingy. Hereditas..

[4] Gustafsson Å (1940). The mutation system of the chlorophyll apparatus. Lunds Universitets Årsskrift N F Avd.

[5] Hallqvist C (1924). Chlorophyllmutanten bei Gerste. Hereditas..

[6] Nilsson-Ehle H (1913). Einige Beobachtungen über erbliche Variationen der Chlorophylleigenschaft bei den Getreidearten. Zeitschr f indukt Abst und Vererbungslehre..

[7] Nilsson-Ehle H (1922). Über freie kombinationen und koppelung verschiedener chlorophyllerbeinheiten bei Gerste. Hereditas..

[8] Miyake K, Imai Y (1922). Genetic studies in studies in barley I. Bot Mag Tokyo..

[9] Saisho D, Tanno K, Chono M, Honda I, Kitano H, Takeda K (2004). Spontaneous brassinolide-insensitive barley mutants "uzu" adapted to East Asia. Breed Sci..

[10] Muller HJ (1927). Artificial transmutation of the gene. Science..

[11] Gustafsson Å (1941). Mutation experiments in barley. Hereditas..

[12] Stadler LJ (1928). Mutations in barley induced by x-rays and radium. Science..

[13] Gustafsson Å, Mac Key J (1948). The genetical effects of mustard gas substances and neutrons. Hereditas..

[14] Gustafsson Å, Nybom N (1949). Colchicine, X-rays and the mutation process. Hereditas..

[15] Stadler LJ (1930). Some genetic effects of X-rays in plants. J Hered..

[16] Gustafsson Å, Ekman G. Yield efficiency of the X-ray mutant Svalöf’s “Pallas barley.” Züchter/Genet Breed Res. 1967;37:42–6.

[17] Dormling I, Gustafsson Å, Jung HR, von Wettstein D (1966). Phytotron cultivation of Svalöf’s Bonus barley and its mutant Svalöf’s Mari. Hereditas..

[18] van Hintum T, Menting F, von Bothmer R, van Hintum T, Knüpffer H, Sato K (2003). Diversity in ex situ genebank collections of barley. Diversity in Barley (Hordeum vulgare).

[19] Jiang C, Lei M, Guo Y, Gao G, Shi L, Jin Y (2022). A reference-guided TILLING by amplicon-sequencing platform supports forward and reverse genetics in barley. Plant Commun..

[20] Knudsen S, Wendt T, Dockter C, Thomsen HC, Rasmussen M, Egevang Jørgensen M (2022). FIND-IT: Accelerated trait development for a green evolution. Sci Adv..

[21] Huang L, Gao G, Jiang C, Guo G, He Q, Zong Y (2023). Generating homozygous mutant populations of barley microspores by ethyl methanesulfonate treatment. aBIOTECH..

[22] Hisano H, Abe F, Hoffie RE, Kumlehn J (2021). Targeted genome modifications in cereal crops. Breed Sci..

[23] Druka A, Franckowiak J, Lundqvist U, Bonar N, Alexander J, Houston K (2011). Genetic dissection of barley morphology and development. Plant Physiol..

[24] Chmielewska B, Janiak A, Karcz J, Guzy-Wrobelska J, Forster BP, Nawrot M (2014). Morphological, genetic and molecular characteristics of barley root hair mutants. J Appl Genet..

[25] Franckowiak JD, Lundqvist U (2019). Rules for nomenclature and gene symbolization in barley. Barley Gen Newsl..

[26] Chono M, Honda I, Zeniya H, Yoneyama K, Saisho D, Takeda K (2003). A semidwarf phenotype of barley uzu results from a nucleotide substitution in the gene encoding a putative brassinosteroid receptor. Plant Physiol..

[27] Dockter C, Gruszka D, Braumann I, Druka A, Druka I, Franckowiak J (2014). Induced variations in brassinosteroid genes define barley height and sturdiness, and expand the green revolution genetic toolkit. Plant Physiol..

[28] Takahashi R (1955). The origin and evolution of cultivated barley. Adv Genet..

[29] Kucera J, Lundqvist U, Gustafsson A (1975). Induction of breviaristatum mutants in barley. Hereditas..

[30] Komatsuda T, Pourkheirandish M, He C, Azhaguvel P, Kanamori H, Perovic D (2007). Six-rowed barley originated from a mutation in a homeodomain-leucine zipper I-class homeobox gene. Proc Natl Acad Sci U S A..

[31] Jia Z, Liu Y, Gruber BD, Neumann K, Kilian B, Graner A (2019). Genetic dissection of root system architectural traits in spring barley. Front Plant Sci..

[32] Rogers ED, Benfey PN (2015). Regulation of plant root system architecture: implications for crop advancement. Curr Opin Biotechnol..

[33] Hochholdinger F, Yu P, Marcon C (2018). Genetic Control of Root System Development in Maize. Trends Plant Sci..

[34] Åberg E, Wiebe GA (1946). Classification of barley varieties grown in the United States and Canada. USDA Technical Bulletins..

[35] Takahashi R, Hayashi J, Moriya I, Yasuda S (1982). Studies on classification and inheritance of barley varieties having awnless or short-awned lateral spikelets (Bozu barley). I. Variation of awn types and classification. Nogaku Kenyu..

[36] Woodward RW (1949). The inheritance of fertility in the lateral florets of the four barley groups. Agron J..

[37] Harlan HV (1918). The identification of varieties of barley. Bull U S Dep Agric..

[38] Mansfeld R (1950). Das morphologische System der Saatgerste, Hordeum vulgare L. s. l. Der Züchter..

[39] Mansfeld R (1959). Hordeum. Die. Kulturpflanze..

[40] Hoffmann W (1959). Gerste (Hordeum vulgare L.).

[41] Stubbe H, Bandlow G. Mutationsveruche an Kulturpflanzen, Röntgenbestrahlungen von Winter- und Sommergersten. Der Züchter. 1946/1947;17/18:365–74.

[42] Franckowiak JD, Lundqvist U (2012). Descriptions of barley genetic stocks for 2012. Barley Gen Newsl..

[43] Harlan HV, Hayes HK (1920). Occurrence of the fixed intermediate, Hordeum intermedium haxtoni, in crosses between H. vulgare pallidium and H. distichum palmella. J Agric Res..

[44] Sakuma S, Lundqvist U, Kakei Y, Thirulogachandar V, Suzuki T, Hori K (2017). Extreme suppression of lateral floret development by a single amino acid change in the VRS1 transcription factor. Plant Physiol..

[45] Lundqvist U (1992). Mutation research in barley: Swedich University of Agricultural Sciences.

[46] Gustafsson Å, Lundqvist U (1980). Hexastichon and intermedium mutants in barley. Hereditas..

[47] Lundqvist U, Lundqvist A (1988). Induced intermedium mutants in barley: origin, morphology and inheritance. Hereditas..

[48] Bull H, Casao MC, Zwirek M, Flavell AJ, Thomas WTB, Guo W (2017). Barley SIX-ROWED SPIKE3 encodes a putative Jumonji C-type H3K9me2/me3 demethylase that represses lateral spikelet fertility. Nat Commun..

[49] van Esse GW, Walla A, Finke A, Koornneef M, Pecinka A, von Korff M (2017). Six-rowed spike3 (VRS3) is a histone demethylase that controls lateral spikelet development in barley. Plant Physiol..

[50] Franckowiak JD, Lundqvist U (2014). Descriptions. Barley Gen Newsl..

[51] Franckowiak JD, Lundqvist U (2017). Descriptions of barley genetic stocks. Barley Gen Newsl..

[52] Youssef HM, Eggert K, Koppolu R, Alqudah AM, Poursarebani N, Fazeli A (2017). VRS2 regulates hormone-mediated inflorescence patterning in barley. Nat Genet..

[53] Ramsay L, Comadran J, Druka A, Marshall DF, Thomas WT, Macaulay M (2011). INTERMEDIUM-C, a modifier of lateral spikelet fertility in barley, is an ortholog of the maize domestication gene TEOSINTE BRANCHED 1. Nat Genet..

[54] Lundqvist U, Lundqvist A (1988). Gene interaction of induced intermedium mutations of two-row barley I. Double mutant recombinations. Hereditas..

[55] Franckowiak JD, Lundqvist U (2013). Full descriptions of barley genetic stocks. Barley Gen Newsl..

[56] Franckowiak JD, Lundqvist U (2018). Detailed descriptions of new and revised Barley Genetic Stocks in Table 1. Barley Gen Newsl..

[57] Åberg E, Wiebe GA (1945). Irregular barley, Hordeum irregulare, sp. J Wash Acad Sci..

[58] Youssef HM, Koppolu R, Rutten T, Korzun V, Schweizer P, Schnurbusch T (2014). Genetic mapping of the labile (lab) gene: a recessive locus causing irregular spikelet fertility in labile-barley (Hordeum vulgare convar. labile). Theor Appl Genet..

[59] Lundqvist U, Abebe B, Lundqvist A (1989). Gene interaction of induced intermedium mutations of two-row barley. V. Tripple gene constellations of the hex-v gene and recessive int genes. Hereditas..

[60] Zakhrabekova S, Chauhan P, Dockter C, Ealumalai P, Ivanova A, Egevang Jørgensen M (2023). Identification of a candidate dwarfing gene in Pallas, the first commercial barley cultivar generated through mutational breeding. Front Genet..

[61] Zakhrabekova S, Dockter C, Ahmann K, Braumann I, Gough SP, Wendt T (2015). Genetic linkage facilitates cloning of Ert-m regulating plant architecture in barley and identified a strong candidate of Ant1 involved in anthocyanin biosynthesis. Plant Mol Biol..

[62] Hagberg A, Gustafsson Å, Ehrenberg L (1958). Sparsely contra densely ionizing radiations and the origin of erectoid mutants in barley. Hereditas..

[63] Houston K, McKim SM, Comadran J, Bonar N, Druka I, Uzrek N (2013). Variation in the interaction between alleles of HvAPETALA2 and microRNA172 determines the density of grains on the barley inflorescence. Proc Natl Acad Sci U S A..

[64] Nair SK, Wang N, Turuspekov Y, Pourkheirandish M, Sinsuwongwat S, Chen G (2010). Cleistogamous flowering in barley arises from the suppression of microRNA-guided HvAP2 mRNA cleavage. Proc Natl Acad Sci U S A..

[65] Persson G, Hagberg A (1969). Induced variation in a quantitative character in barley. Morphology and cytogenetics of erectoides mutants. Hereditas..

[66] Scholz F, Lehmann O (1958). Die Gaterslebener Mutanten der Saatgerste in Beziehung zur Formenmannigfaltigkeit der Art Hordeum vulgare L.s.l. I. Kulturpflanze..

[67] Ullrich SE, Aydin A, editors. Mutation breeding for semi-dwarfism in barley. In: Semi-dwarf Cereal Mutants and Their Use in Cross-breeding III. IAEA-TECDOC-455; 1988; IAEA, Vienna, Austria.

[68] Swenson SP, Wells DG (1944). The linkage relation of four genes in chromosome 1 of barley. J Am Soc Agron..

[69] Tavcar A (1938). Vererbungsart der Spindelstufenzahl bei Bastardierungen einiger distichum x vulgare Wintergersten. Z Indukt Abstammungs Vererbungsl..

[70] Jost M, Taketa S, Mascher M, Himmelbach A, Yuo T, Shahinnia F (2016). A homolog of Blade-On-Petiole 1 and 2 (BOP1/2) controls internode length and homeotic changes of the barley inflorescence. Plant Physiol..

[71] Kasha KJ, Walker GWR (1960). Several recent barley mutants and their linkages. Can J Genet Cytol..

[72] Franckowiak JD (1992). Allelism tests among selected semidwarf barleys. Barley Gen Newsl..

[73] Franckowiak JD, Lundqvist U (2019). Detailed descriptions of new and revised Barley Genetic Stocks in Table 1. Barley Gen Newsl..

[74] Larsson HEB (1981). Branching spike mutants from two loci in two-row barley. Barley Gen Newsl..

[75] Poursarebani N, Seidensticker T, Koppolu R, Trautewig C, Gawronski P, Bini F, et al. The genetic basis of composite spike form in barley and “Miracle-Wheat.” Genetics. 2015;201:155–65. 10.1534/genetics.115.176628 PMC4566260 26156223

[76] Dabbert T, Okagaki RJ, Cho S, Boddu J, Muehlbauer GJ (2009). The genetics of barley low-tillering mutants: absent lower laterals (als). Theor Appl Genet..

[77] Walker GWR, Dietrich J, Miller R, Kasha KJ (1963). Recent barley mutants and their linkages II. Genetic data for further mutants. Can J Genet Cytol..

[78] Walker GWR, Kasha K, Miller RA (1958). Recombination studies in barley. Proc Genet Soc Can..

[79] Bossinger G, Lundqvist U, Rohde W, Salamini F, Munck L, Kirkegaard K, Jensen B (1992). Genetics of plant development in barley. Sixth Int Barley Genet Symp.

[80] Robertson DW (1967). Linkage studies of various barley mutations (Hordeum species). Crop Sci..

[81] Takahashi R, Hayashi J, Moriya I. New find of an allele Rt’ semi-dominant over rt for rattail spike. Barley Gen Newsl. 1976;6:74.

[82] Pourkheirandish M, Hensel G, Kilian B, Senthil N, Chen G, Sameri M (2015). Evolution of the Grain Dispersal System in Barley. Cell..

[83] Takahashi R, Hayashi J (1959). Linkage study of the complementary genes for brittle rachis in barley. Preliminary Note Nokaku Kenkyu..

[84] Tavcar A (1944). The inheritance of the firmness of the spikelets on naked barley, H. sativum nudum. Rev Sci Agr, Zagreb..

[85] Nybom N (1954). Mutation types in barley. Acta Agric Scand..

[86] Tsuchiya T (1974). Further results of allelism testing in barley. Barley Gen Newsl..

[87] Tsuchiya T (1974). Preliminary results of allelism testing in barley. Barley Gen Newsl..

[88] Hor KS (1924). Interrelations of genetic factors in barley. Genetics..

[89] Neatby KW (1926). Inheritance of quantitative and other characters in a barley cross. Sci Agric..

[90] Neatby KW (1929). An analysis of the inheritances of quantitative characters and linkage in barley. Sci Agric..

[91] Pozzi C, di Pietro D, Halas G, Roig C, Salamini F (2003). Integration of a barley (Hordeum vulgare) molecular linkage map with the position of genetic loci hosting 29 developmental mutants. Heredity (Edinb)..

[92] Franckowiak JD, Lundqvist U (2010). Descriptions of barley genetic stocks for 2010. Barley Gen Newsl..

[93] Ziegler A (1920). Variationen in der Form der Basalborste. Missbildungen und Abänderungen am Typus und deren Vererbung. Ill Landw Z..

[94] Buckley GFH (1930). Inheritance in barley with special reference to the color of the caryopsis and lemma. Sci Agric..

[95] Engledow FL (1920). Inheritance in barley. I. The lateral florets and rachilla. J Genet..

[96] Wiebe GA (1972). “Stubble” gene. Barley Gen Newsl..

[97] Pozzi C, Faccioli P, Terzi V, Stanca AM, Cerioli S, Castiglioni P (2000). Genetics of mutations affecting the development of a barley floral bract. Genetics..

[98] Martini ML, Harlan HV (1942). Barley freaks. J Hered..

[99] von Ubisch G (1919). Beitrag zu einer Faktorenanalyse von Gerste. II. Z Indukt Abstammungs Vererbungsl..

[100] Wexelsen H (1934). Quantitative inheritance and linkage in barley. Hereditas..

[101] Takahashi R, Hayashi J (1966). Inheritance and linkage studies in barley. II. Assignment of several new mutant genes to their respective linkage groups by the trisomic method of analysis. Ber Ohara Inst landw Biol, Okayama Univ..

[102] Ahokas H (1977). A mutant of barley: Awned palea. Barley Gen Newsl..

[103] Forster BP, Franckowiak JD, Lundqvist U, Lyon J, Pitkethly I, Thomas WTB (2007). The barley phytomer. Ann Bot..

[104] Yoshikawa T, Hisano H, Hibara KI, Nie J, Tanaka Y, Itoh JI (2022). A bifurcated palea mutant infers functional differentiation of WOX3 genes in flower and leaf morphogenesis of barley. AoB Plants..

[105] Litzenberg SC, Green JM (1951). Inheritance of awns in barley. Agron J..

[106] Walpole PR, Morgan DG (1972). Physiology of grain filling in barley. Nature..

[107] Yuo T, Yamashita Y, Kanamori H, Matsumoto T, Lundqvist U, Sato K (2012). A SHORT INTERNODES (SHI) family transcription factor gene regulates awn elongation and pistil morphology in barley. J Exp Bot..

[108] Braumann I, Dockter C, Beier S, Himmelbach A, Lok F, Lundqvist U (2017). Mutations in the gene of the Ga subunit of the heterotrimeric G protein are the cause for brachytic1 semi-dwarf phenotype in barley and applicable for practical breeding. Hereditas..

[109] Wendt T, Holme I, Dockter C, Preuss A, Thomas W, Druka A (2016). HvDep1 is a positive regulator of culm elongation and grain size in barley and impacts yield in an environment-dependent manner. PLoS One..

[110] Ellis RP, Forster BP, Gordon DC, Handley LL, Keith RP, Lawrence P (2002). Phenotype/genotype associations for yield and salt tolerance in a barley mapping population segregating for two dwarfing genes. J Exp Bot..

[111] Forster BP, Pakniyat H, Macaulay M, Matheson W, Phillips MS, Thomas WTB (1994). Variation in the leaf sodium content of the Hordeum vulgare (barley) cultivar Maythorpe and its derived mutant c.v. Heredity..

[112] Pakniyat H, Baird E, Thomas WTB, Caligari PDS, Powell W, Forster BP, Slinkard AE, Scoles GJ, Rossnagel BG (1996). Effect of semi-dwarf mutants on salt tolerance in barley. Fifth Int Oat Conf & Seventh Int Barley Genet Symp.

[113] Liller CB, Walla A, Boer MP, Hedley P, Macaulay M, Effgen S (2017). Fine mapping of a major QTL for awn length in barley using a multiparent mapping population. Theor Appl Genet..

[114] Harlan HV (1920). Smooth-awned barleys. J Am Soc Agron..

[115] Franckowiak J (1995). Notes on linkage drag in Bowman backcross derived lines of spring barley. Barley Gen Newsl..

[116] Konishi T (1971). A new smooth awn gene on chromosome 6. Barley Gen Newsl..

[117] Milner SG, Jost M, Taketa S, Mazon ER, Himmelbach A, Oppermann M (2019). Genebank genomics highlights the diversity of a global barley collection. Nat Genet..

[118] Woodward RW (1949). Sterility in Velvon barley and its relationship to yield, kernel weight, and date and rate of seeding. Agron J..

[119] Austenson HM (1948). Linkage relations of the male sterile gene ms2 in barley.

[120] Engledow FL. Inheritance in barley. III. The awn and the lateral floret (cont’d): fluctuation: a linkage: multiple allelomorphs. J Genet. 1924;14:49–87.

[121] Tsuchiya T, Singh RJ (1972). Another case of paracentric inversion in a genetic stock, Engleawnless, for Lk of barley. Barley Gen Newsl..

[122] Wiebe GA (1972). Tight linkage of the awnless gene in Engleawnless with the Vv locus. Barley Gen Newsl..

[123] Qualset CO, Schaller CW, Williams JC (1965). Performance of isogenic lines of barley as influenced by awn length, linkage blocks, and environment. Crop Sci..

[124] Schaller CW, Qualset CO (1975). Isogenic analysis of productivity in barley: Interaction of genes affecting awn length and leaf-spotting. Crop Sci..

[125] Schaller CW, Qualset CO, Ruther NJ (1972). Isogenic analysis of the effects of the awn on productivity of barley. Crop Sci..

[126] Konishi T, Hayashi J, Moriya I, Takahashi R (1984). Inheritance and linkage studies in barley VII. Location of six new mutant genes on chromosome 3. Ber Ohara Inst landw Biol, Okayama Univ..

[127] Moriya I, Takahashi R (1980). Linkage studies of three barley mutants. Barley Gen Newsl..

[128] Stebbins GL, Yagil E (1966). The morphogenic effects of the hooded gene in barley. I. The course of development in hooded and awned genotypes. Genetics..

[129] Takahashi R, Hayashi J, Hirao C (1967). Linkage studies. Barley Gen Newsl..

[130] Yagil E, Stebbins GL (1969). The morphogenetic effects of the hooded gene in barley. II. Cytological and environmental factors affecting gene expression. Genetics..

[131] Müller KJ, Romano N, Gerstner O, Garcia-Maroto F, Pozzi C, Salamini F (1995). The barley Hooded mutation caused by a duplication in a homeobox gene intron. Nature..

[132] Franckowiak J, Forster BP, Lundqvist U, Lyon J, Pitkethly I, Thomas WTB. Developmental mutants as a guide to the barley phytomer. In: Cerrarelli S, Grando S, editors. 10th International Barley Genetic Symposium; Alexandria, Egypt. : ICARDA, PO Box 5466, Aleppo, Syria; 2010. 46–60.

[133] Hartwig T, Corvalan C, Best NB, Budka JS, Zhu JY, Choe S (2012). Propiconazole is a specific and accessible brassinosteroid (BR) biosynthesis inhibitor for Arabidopsis and maize. PLoS One..

[134] Gruszka D, Szarejko I, Maluszynski M (2011). New allele of HvBRI1 gene encoding brassinosteroid receptor in barley. J Appl Genet..

[135] Dockter C, Hansson M (2015). Improving barley culm robustness for secured crop yield in a changing climate. J Exp Bot..

[136] Hellewell KB, Rasmusson DC, Gallo-Meagher M (2000). Enhancing yield of semidwarf barley. Crop Science..

[137] Xu Y, Jia Q, Zhou G, Zhang XQ, Angessa T, Broughton S (2017). Characterization of the sdw1 semi-dwarf gene in barley. BMC Plant Biol..

[138] Tsuchiya T (1974). Root character of curly mutants in barley. Barley Gen Newsl..

[139] Takahashi R, Mochizuki A, Hayashi J (1959). Heritable mixoploidy in barley II. On the semi-minute. Nokaku Kenkyu..

[140] Takahashi R, Mochizuki A, Hayashi J (1955). Heritable mixoploidy in barley. Nokaku Kenkyu..

[141] Hibara KI, Miya M, Benvenuto SA, Hibara-Matsuo N, Mimura M, Yoshikawa T (2021). Regulation of the plastochron by three many-noded dwarf genes in barley. PLoS Genet..

[142] Mascher M, Jost M, Kuon JE, Himmelbach A, Assfalg A, Beier S (2014). Mapping-by-sequencing accelerates forward genetics in barley. Genome Biol..

[143] Gaul H (1964). Mutations in plant breeding. Rad Bot..

[144] Schmalz H (1962). Makromutationen bei Sommergerste und Sommerweizen. Züchter..

[145] Takahashi R, Moriya I (1973). Two new mutant genes on chromosome 4. Barley Gen Newsl..

[146] Shands RG (1963). Inheritance and linkage of orange lemma and uniculm characters. Barley Newsl..

[147] Babb S, Muehlbauer GJ (2003). Genetic and morphological characterization of the barley uniculm2 (cul2) mutant. Theor Appl Genet..

[148] Kasha KG, Falk DE, Ho-Tsai A (1978). Linkage data with genes on chromosome 6. Barley Gen Newsl..

[149] Dofing SM, Slinkard AE, Scoles GJ, Rossnagel BG (1996). Near-isogenic analysis of uniculm and conventional-tillering barley lines. Seventh Int Barley Genet Symp.

[150] Okagaki RJ, Cho S, Kruger WM, Xu WW, Heinen S, Muehlbauer GJ (2013). The barley UNICULM2 gene resides in a centromeric region and may be associated with signaling and stress responses. Funct Integr Genomics..

[151] Franckowiak JD, Lundqvist U, Kleinhofs A (2016). Descriptions. Barley Gen Newsl..

[152] Rossini L, Vecchietti A, Nicoloso L, Stein N, Franzago S, Salamini F (2006). Candidate genes for barley mutants involved in plant architecture: an in silico approach. Theor Appl Genet..

[153] Tavakol E, Okagaki R, Verderio G, Shariati JV, Hussien A, Bilgic H (2015). The barley Uniculme4 gene encodes a BLADE-ON-PETIOLE-like protein that controls tillering and leaf patterning. Plant Physiol..

[154] Nonaka S (1973). A new type of cultivar, Mitake, with very few in number, but thick and stiff culms. Barley Gen Newsl..

[155] Dabbert T, Okagaki RJ, Cho S, Heinen S, Boddu J, Muehlbauer GJ (2010). The genetics of barley low-tillering mutants: low number of tillers-1 (lnt1). Theor Appl Genet..

[156] Burton RA, Ma G, Baumann U, Harvey AJ, Shirley NJ, Taylor J (2010). A customized gene expression microarray reveals that the brittle stem phenotype fs2 of barley is attributable to a retroelement in the HvCesA4 cellulose synthase gene. Plant Physiol..

[157] Kokubo A, Kuraishi S, Sakurai N (1989). Culm strength of barley: correlation among maximum bending stress, cell wall dimensions, and cellulose content. Plant Physiol..

[158] Kokubo A, Sakurai N, Kuraishi S, Takeda K (1991). Culm brittleness of barley (Hordeum vulgare L.) mutants is caused by smaller number of cellulose molecules in cell wall. Plant Physiol..

[159] Kimura S, Sakurai N, Itoh T (1999). Different distribution of cellulose synthesizing complexes in brittle and non-brittle strains of barley. Plant Cell Physiol..

[160] Toyota M, Gilroy S (2013). Gravitropism and mechanical signaling in plants. Am J Bot..

[161] Zhou Y, Zhou G, Broughton S, Westcott S, Zhang X, Xu Y (2018). Towards the identification of a gene for prostrate tillers in barley (Hordeum vulgare L .). PLoS One..

[162] Konishi T (1975). Characteristics and inheritance of EMS-induced mutants in barley. Nogaku Kenyu..

[163] Takahashi R, Hayashi J, Konishi T, Moriya I (1975). Linkage analysis of barley mutants. Barley Gen Newsl..

[164] Yoshikawa T, Tanaka SY, Masumoto Y, Nobori N, Ishii H, Hibara K (2016). Barley NARROW LEAFED DWARF1 encoding a WUSCHEL-RELATED HOMEOBOX 3 (WOX3) regulates the marginal development of lateral organs. Breed Sci..

[165] Jöst M, Hensel G, Kappel C, Druka A, Sicard A, Hohmann U (2016). The INDETERMINATE DOMAIN protein BROAD LEAF1 limits barley leaf width by restricting lateral proliferation. Curr Biol..

[166] Peng S, Khush GS, Virk P, Tang Q, Zou Y (2008). Progress in ideotype breeding to increase rice yield potential. Field Crops Res..

[167] Richards RA (2000). Selectable traits to increase crop photosynthesis and yield of grain crops. J Exp Bot..

[168] Cho SH, Yoo SC, Zhang H, Pandeya D, Koh HJ, Hwang JY (2013). The rice narrow leaf2 and narrow leaf3 loci encode WUSCHEL-related homeobox 3A (OsWOX3A) and function in leaf, spikelet, tiller and lateral root development. New Phytol..

[169] Fujino K, Matsuda Y, Ozawa K, Nishimura T, Koshiba T, Fraaije MW (2008). NARROW LEAF 7 controls leaf shape mediated by auxin in rice. Mol Genet Genomics..

[170] Hu J, Zhu L, Zeng D, Gao Z, Guo L, Fang Y (2010). Identification and characterization of NARROW AND ROLLED LEAF 1, a novel gene regulating leaf morphology and plant architecture in rice. Plant Mol Biol..

[171] Jiang F, Guo M, Yang F, Duncan K, Jackson D, Rafalski A (2012). Mutations in an AP2 transcription factor-like gene affect internode length and leaf shape in maize. PLoS One..

[172] Nardmann J, Ji J, Werr W, Scanlon MJ (2004). The maize duplicate genes narrow sheath1 and narrow sheath2 encode a conserved homeobox gene function in a lateral domain of shoot apical meristems. Development..

[173] Qi J, Qian Q, Bu Q, Li S, Chen Q, Sun J (2008). Mutation of the rice Narrow leaf1 gene, which encodes a novel protein, affects vein patterning and polar auxin transport. Plant Physiol..

[174] Gustafsson Å, Hagberg A, Lundqvist U, Persson G (1969). A proposed system of symbols for the collectionn of barley mutants at Svalöv. Hereditas..

[175] Fischbeck G, Haüser H (1976). Research notes. Barley Gen Newsl..

[176] Tsuchiya T, Haines RL (1975). Trisomic analysis of nine mutant genes in barley. Barley Gen Newsl..

[177] Ripley BS, Pammenter NW, Smith VR (1999). Function of leaf hairs revisited: The hair layer on leaves Arctotheca populifolia reduces photoinhibition, but leads to higher leaf temperatures caused by lower transpiration rates. J Plant Physiol..

[178] Stipanovic RD. Function and chemistry of plant trichomes and glands in insect resistance. In: Hedin PA, editor. Plant resistance to insects. 208: American Chemical Society; 1983. 69–100.

[179] Patterson FL, Shands RG (1957). Independant inheritance of four characters in barley. Agron J..

[180] Häuser J, Fischbeck G (1976). Untersuchungen zur Lokalisierung einiger Mutationen von Gerste (Hordeum sativum). Z Pflanzenzücht..

[181] Hauser J, Jahoor A, Fischbeck G (1988). Localization of induced mutants for globe shaped grains. Barley Gen Newsl..

[182] Franckowiak JD, Lundqvist U (2015). Descriptions. Barley Gen Newsl..

[183] Jarvi AJ (1970). Shrunken endosperm mutants in barley.

[184] Jarvi AJ, Eslick R (1975). Shrunken endosperm mutants in barley. Crop Science..

[185] Ramage RT, Crandall CL (1981). Shrunken endosperm mutant seg8. Barley Gen Newsl..

[186] Ramage RT, Eslick RF (1975). Shrunken endosperm, xenia, se6. Barley Gen Newsl..

[187] Jensen J (1979). Chromosomal location of one dominant and four recessive high-lysine genes in barley mutants. Seed Protein Improvement in Cereals and Grain Legumes; Neuherberg, Germany: Int.

[188] Eslick RF, Hockett EA (1976). Description of shrunken endosperm mutant sex 1. Barley Gen Newsl..

[189] Röder MS, Kaiser C, Weschke W (2006). Molecular mapping of the shrunken endosperm genes seg8 and sex1 in barley (Hordeum vulgare L.). Genome..

[190] Jende-Strid B. Coordinator’s report: Anthocyanin genes. Barley Gen Newsl. 1995;24:162–5.

[191] Felker FC, Peterson DM, Nelson OE (1985). Anatomy of immature grains of eight meternal effect shrunken endosperm barley mutants. Am J Bot..

[192] Jung W, Skadsen RW, Peterson DM (2001). Characterization of a novel barley β-amylase gene expressed only during early grain development. Seed Sci Res..

[193] Ramage RT (1971). Translocations and balanced tertiary trisomics. Barley Gen Newsl..

[194] Ramage RT (1983). Chromosome location of shrunken endosperm mutants seg6g and seg8k. Barley Gen Newsl..

[195] Ramage RT, Scheuring JF (1976). Shrunken endosperm mutants Seg6 and Seg7. Barley Gen Newsl..

[196] Biyashev RM, Netsvetaev VP, Sozinov AA (1986). Genetic control of some morphological markers for qualitative and biochemical characters and location of three genetic factors on chromosomes 1 and 5 of barley Hordeum vulgare L. Sov Genet..

[197] Eslick RF, Ries MN (1976). Positioning sex1 on chromosome 6. Barley Gen Newsl..

[198] Falk DE, Swartz MJ, Kasha KJ (1980). Linkage data with genes near the centromere of barley chromosome 6. Barley Gen Newsl..

[199] Netsvetaev VP (1992). Use of double ditelosomics for gene location in barley. Cytol Gen (Kiev).

[200] Scheuring JF, Ramage RT (1976). A tertiary trisomic balanced for both msg2 and sex2. Barley Gen Newsl..

[201] Helback H (1959). Domestication of food plants in the Old World. Science..

[202] Lister DL, Jones H, Jones MK, O’Sullivan DM, Cockram J. Analysis of DNA polymorphism in ancient barley herbarium material: validation of the KASP SNP genotyping platform. Taxon. 2013;62:779–89.

[203] Taketa S, Amano S, Tsujino Y, Sato T, Saisho D, Kakeda K (2008). Barley grain with adhering hulls is controlled by an ERF family transcription factor gene regulating a lipid biosynthesis pathway. Proc Natl Acad Sci U S A..

[204] Takahashi R, Yamamoto J, Yasuda S, Itano Y (1953). Inheritance and linkage studies in barley. Ber Ohara Inst landw Forsch..

[205] Wabila C, Neumann K, Kilian B, Radchuk V, Graner A (2019). A tiered approach to genome-wide association analysis for the adherence of hulls to the caryopsis of barley seeds reveals footprints of selection. BMC Plant Biol..

[206] Larsson HEB (1985). Morphological analysis of laxatum barley mutants. Hereditas..

[207] Roath WW, Hockett EA (1971). Genetic male sterility in barley. III. Pollen and anther characteristics. Crop Sci..

[208] Scholz F (1956). Mutationsversuche an Kulturpflanzen. V. Die Vererbung zweier sich variabel manifestierender Übergangsmerkmale von bespelzter zu nackter Gerste bei röntgeninduzierten Mutanten. Kulturpflanze..

[209] Colas I, Macaulay M, Higgins JD, Phillips D, Barakate A, Posch M (2016). A spontaneous mutation in MutL-Homolog 3 (HvMLH3) affects synapsis and crossover resolution in the barley desynaptic mutant des10. New Phytol..

[210] Hernandez-Soriano JM, Ramage RT. Coordinator’s report Desynaptic genes. Barley Gen Newsl. 1974;4:123–5.

[211] Soule J, Skodova I, Kudrna D, Kilian A, Kleinhofs A (1995). Molecular and genetic characterization of barley flower development mutants. Barley Gen Newsl..

[212] Comadran J, Kilian B, Russell J, Ramsay L, Stein N, Ganal M (2012). Natural variation in a homolog of Antirrhinum CENTRORADIALIS contributed to spring growth habit and environmental adaptation in cultivated barley. Nat Genet..

[213] Matyszczak I, Tominska M, Zakhrabekova S, Dockter C, Hansson M (2020). Analysis of early-flowering genes at barley chromosome 2H expands the repertoire of mutant alleles at the Mat-c locus. Plant Cell Rep..

[214] Zakhrabekova S, Gough SP, Braumann I, Muller AH, Lundqvist J, Ahmann K (2012). Induced mutations in circadian clock regulator Mat-a facilitated short-season adaptation and range extension in cultivated barley. Proc Natl Acad Sci U S A..

[215] Gustafsson Å (1951). Mutations, environment and evolution. Induction of changes in genes and chromosomes II; Cold Spring Herbor Symposia on Quantitative Biology.

[216] Takahashi R, Yasuda S, Nilan RA (1969). Genetics of earliness and growth habit in barley. Second International Barley Genetic Symposium: Washington State University Press.

[217] Yasuda S (1977). Linkage of earliness gene eak and its pleiotropic effects under different growth conditions. Ber Ohara Inst landw Biol, Okayama Univ..

[218] Sigurbjörnsson B, Gaul H (1975). Methods of mutation induction, including efficiency, and utilization of induced genetic variability. Barley Genetics II.

[219] von Wettstein-Knowles P. Plant waxes. Encyclopedia of Life Sciences. Chichester: Wiley 2016.

[220] Eigenbrode SD, Kerstiens G (1996). Plant surface waxes and insect behaviour. Plant cuticles - an integrated functional approach.

[221] Kerstiens G (1996). Signaling across the divide: a wider perspective of cuticular structure-function relationships. Trends Plant Sci..

[222] Mariani C, Wolters-Arts M (2000). Complex waxes. Plant Cell..

[223] Beattie GA, Marcell LM (2002). Effect of alterations in cuticular wax biosynthesis on the physiochemical properties and topography of maize leaf surfaces. Plant Cell Environ..

[224] Oliviera AFM, Meirelles ST, Salatino A (2003). Epicuticular waxes from caatinga and cerrado species and their efficiency against water loss. An Acad Bras Cienc..

[225] Riederer M, Schreiber L (2001). Protecting against water loss: analysis of the barrier properties of plant cuticles. J Exp Bot..

[226] Ristic Z, Jenks MA (2002). Leaf cuticle and water loss in maize lines differing in dehydration avoidance. J Plant Physiol..

[227] Lundqvist U, Lundqvist A (1988). Mutagen specificity in barley for 1580 eceriferum mutants localized to 79 loci. Hereditas..

[228] Lundqvist U, von Wettstein D (1962). Induction of eceriferum mutants in barley by ionizing radiations and chemical mutagens. Hereditas..

[229] von Wettstein-Knowles P (1976). Biosynthetic relationships between β-diketones and esterified alkan-2-ols deduced from epicuticular wax of barley mutants. Mol Gen Genet..

[230] Schneider LM, Adamski NM, Christensen CE, Stuart DB, Vautrin S, Hansson M (2016). The Cer-cqu gene cluster determines three key players in a β-diketone synthase polyketide pathway synthesizing aliphatics in epicuticular waxes. J Exp Bot..

[231] von Wettstein-Knowles P, Søgaard B (1980). The cer-cqu region in barley: gene cluster or mutifunctional gene. Carlsberg Res Commun..

[232] McProud WL, Eslick RF (1971). Allelism within a series of glossy sheath mutants. Barley Gen Newsl..

[233] Rasmusson DC, Lambert JW (1965). Inheritance of the glossy-sheath character in barley Hordeum vulgare L. Crop Sci..

[234] Konishi T (1973). Genetic analyses of EMS-induced mutants in barley. Barley Gen Newsl..

[235] Haus TE, Tsuchiya T (1972). Allelic relationships among glossy seedling mutants. Barley Gen Newsl..

[236] Takahashi R, Hayashi J, Moriya I (1971). Linkage studies in barley. Barley Gen Newsl..

[237] Henningsen KW, Boynton JE, von Wettstein D (1993). Mutants at xantha and albina loci in relation to chloroplast biogenesis in barley (Hordeum vulgare L.). The Royal Danish Academy of Sciences and Letters..

[238] von Wettstein D, Kahn A, Nielsen OF, Gough S (1974). Genetic regulation of chlorophyll synthesis analyzed with mutants in barley. Science..

[239] Baker NR, Hardwick K (1973). Biochemical and physiological aspects of leaf development in cocoa (Theobroma cacao): I. Development of chlorophyll and photosynthetic activity. New Phytol..

[240] Lee DW, Brammier S, Smith AP (1987). The selective advantage of anthocyanins in developing leaves of mango and cacao. Biotropica..

[241] Noodén LD, Hillsberg JW, Schneider MJ (1996). Induction of leaf senescence in Arabidopsis thaliana by long days through a light-dosage effect. Physiol Plant..

[242] von Wettstein D, Gustafsson Å, Ehrenberg L (1959). Mutationsforschung und Züchtung.

[243] Braumann I, Stein N, Hansson M (2014). Reduced chlorophyll biosynthesis in heterozygous barley magnesium chelatase mutants. Plant Physiol Biochem..

[244] Hansson M, Gough SP, Kannangara CG, von Wettstein D. Analysis of RNA and enzymes of potential importance for regulation of 5-aminolevulinic acid synthesis in the protochlorophyllide accumulating barley mutant tigrina-d^12^. Plant physiology and biochemistry : PPB. 1997;35:827–36.

[245] Nielsen OF (1974). Photoconversion and regeneration of active protochlorophyll(ide) in mutants defective in the regulation of chlorophyll synthesis. Arch Biochem Biophys..

[246] Nielsen OF (1974). Macromolecular physiology of plastids. XII. Tigrina mutants in barley: genetic, spectroscopic and structural characterization. Hereditas..

[247] Delgado-Vargas F, Jimenez AR, Paredes-Lopez O (2000). Natural pigments: carotenoids, anthocyanins, and betalains - characteristics, biosynthesis, processing, and stability. Crit Rev Food Sci Nutr..

[248] Jende-Strid B (1993). Genetic control of flavonoid biosynthesis in barley. Hereditas..

[249] von Bothmer R, van Hintum T, Knüpffer H, Sato K (2003). Diversity in Barley (Hordeum vulgare).

[250] von Wettstein D, Jende-Strid B, Ahrenst-Larsen B, Erdal K (1977). Biochemical mutant in barley renders chemical stabilization of beer superfluous. Carlsberg Res Commun..

[251] Jende-Strid B. Coordinator’s report: Anthocyanin genes. Barley Genet Newsl. 1984;14:76–9.

[252] Zhang L, Lavery L, Gill U, Gill K, Steffenson B, Yan G (2009). A cation/proton-exchanging protein is a candidate for the barley NecS1 gene controlling necrosis and enhanced defense response to stem rust. Theor Appl Genet..

[253] Jensen J, Nilan RA (1971). Mapping of 10 mutant genes for necrotic spotting in barley by means of translocation. Barley Genetics II Proc Second Int Barley Genet Symp, Pullman, WA 1969.

[254] Jensen J, Jørgensen JH (1973). Location of some genes on barley chromosome 5. Barley Genet Newsl..

[255] Woodward RW (1941). Inheritance of melanin-like pigment in the glumes and caryopses of barley. J Agric Res..

[256] Woodward RW (1942). Linkage relationships between the allelomorphic series, B, B^mb^, B^g^, and A_t_a_t_ factors in barley. J Am Soc Agron..

[257] Myler JL, Stanford EH (1942). Color inheritance in barley. J Am Soc Agron..

[258] Bennett AE, Grussu D, Kam J, Caul S, Halpin C (2015). Plant lignin content altered by soil microbial community. New Phytol..

[259] Mullick DB, Fairs DG, Brink VC, Acheson RM (1958). Anthocyanins and anthocyanidins of the barley pericarp and aleurone tissues. Can J Plant Sci..

[260] Finch RA, Simpson E (1978). New colours and complementary colour genes in barley. Z Pflanzenzücht..

[261] Mullick DB, Brink VC (1970). A method for exposing aleurone tissue of barley for color classification. Can J Plant Sci..

